# Merging Photoexcited Nitroarenes with Lewis Acid Catalysis for the Anti-Markovnikov Oxidation of Alkenes

**DOI:** 10.1021/acs.orglett.5c00389

**Published:** 2025-02-20

**Authors:** Joshua M. Paolillo, Mahmoud R. Saleh, Ethan W. Junk, Marvin Parasram

**Affiliations:** Department of Chemistry, New York University, 24 Waverly Place, 3rd Floor, New York, New York 10003, United States

## Abstract

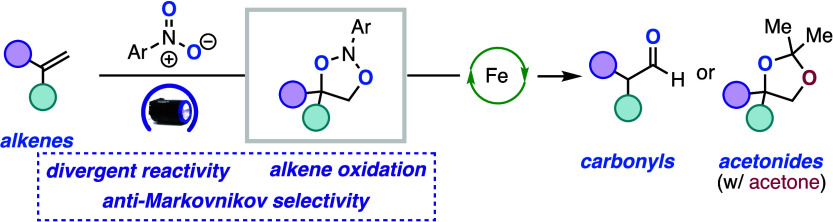

Herein we describe the oxidation of alkenes to carbonyls and acetonides via the interplay of photoexcited nitroarenes and Lewis acid catalysis. A wide range of alkenes were oxidized to aldehyde and ketone products with anti-Markovnikov selectivity and to acetonides when acetone was employed as a co-solvent. Mechanistic studies support that Lewis acid coordination to the 1,3,2-dioxazolidine intermediate results in a 1,2-shift to generate carbonyl derivatives and a nucleophilic substitution pathway for the formation of acetonides.

Oxygen-containing functional groups are ubiquitous in natural products and pharmaceuticals and serve as versatile functional handles in organic synthesis.^1^ The direct conversion of alkenes to carbonyls and alcohols through oxidation reactions using transition metal catalysts represents a powerful strategy to generate oxygen-containing building blocks from abundant feedstock chemicals.^2−4^ The Tsuji–Wacker oxidation is a widely used method for converting alkenes into carbonyls using a palladium catalyst and an oxidant, generally oxidizing the more substituted carbon to generate ketones (Scheme 1A).^5^ Methods for oxidizing the less substituted carbon, commonly termed anti-Markovnikov (AM) Wacker oxidations, are highly desired as they provide access to aldehydes from abundant alkene starting materials.^6^ Under standard Tsuji–Wacker conditions, AM selectivity can be achieved (Scheme 1B). However, high selectivity is typically limited to substrates with functionalities that can act as directing groups for the catalyst, such as protected amines^7^ or cyclic carbonates.^8^ AM selectivity has also been achieved without the use of directing groups under specific catalytic systems. In 1994, Feringa reported that a 2.34:1 aldehyde:ketone ratio could be achieved for the oxidation of terminal alkenes using a palladium nitro complex as the catalyst.^9^ More recently, reports have shown that this palladium nitro catalyst system can be utilized to achieve high aldehyde selectivity for the oxidation of monosubstituted terminal alkenes.^10−13^ It is proposed that the high AM selectivity of this system results from a radical-type addition of nitrite to the alkene. Despite the high selectivity without directing groups, the scope of these methods has been limited to monosubstituted terminal alkenes. Another approach that leads to the formal AM oxidation of alkenes is the Lewis acid-catalyzed Meinwald rearrangement of epoxides (Scheme 1C).^14^ Protocols for the ring opening of mono-, di-, and trisubstituted epoxides that operate under mild reaction conditions and utilize the identity of the Lewis acid to achieve high selectivity of either aldehyde or ketone products have been developed. Additionally, control over the selectivity of an alkyl or hydride shift by the choice of catalyst enables access to α-quaternary aldehydes.^15^ While the Lewis acid-catalyzed rearrangement of the epoxide is typically conducted under mild conditions, the need to first convert alkenes to epoxides makes this a less direct approach. While high AM selectivity has been achieved by various methods, the scope of alkenes amenable to these transformations remains limited. Thus, a general method that can directly oxidize a wide range of alkenes to carbonyls with a high AM selectivity is highly warranted.

**Scheme 1:**
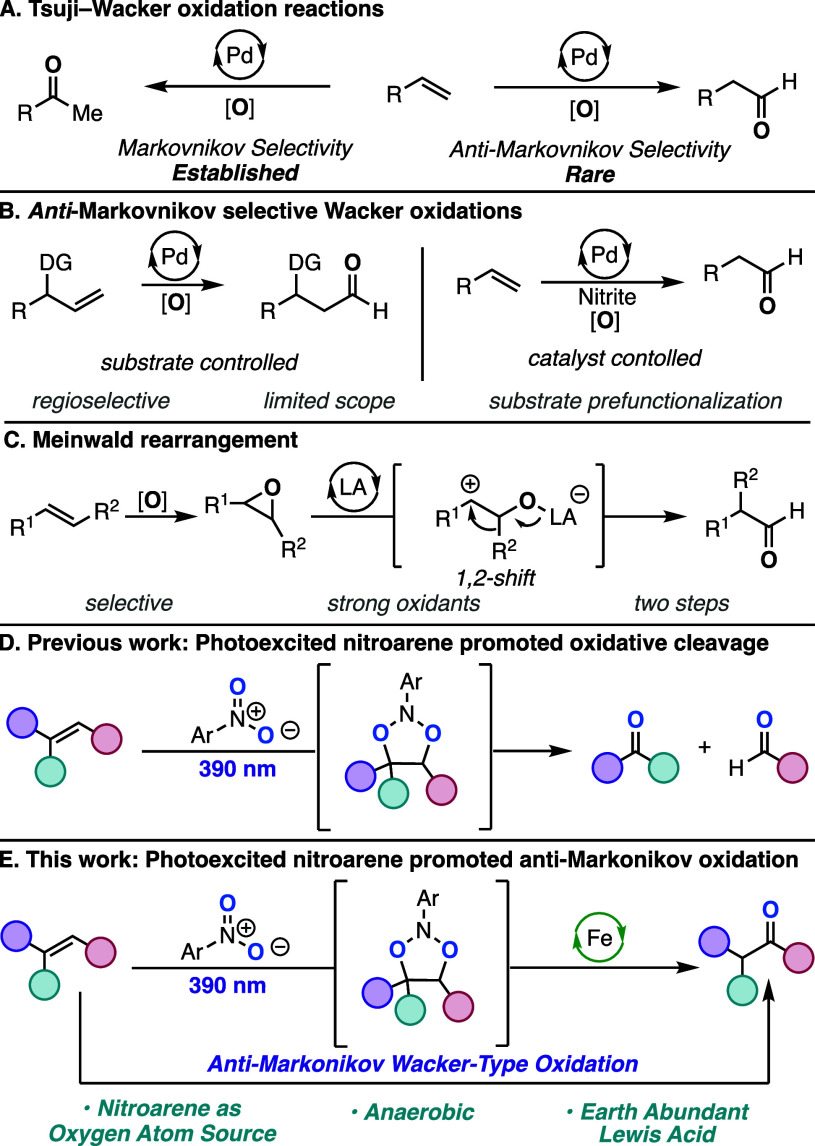
Alkene Oxidations and Hypothesis

Work from our laboratory,^16^ the Leonori group,^17^ the inspiring seminal contributions by Büchi, Hamilton, and De Mayo,^18^ and work by others^19^ have demonstrated the ability of photoexcited nitroarenes to act as mild oxygen transfer reagents. Irradiation with visible light results in the excitation of electron-deficient nitroarenes, followed by intersystem crossing (ISC), to a triplet, diradical excited state, which undergoes a radical [3+2] cycloaddition with alkenes to generate a 1,3,2-dioxazolidine intermediate (Scheme 1D). Fragmentation of this intermediate by retrocycloaddition results in the formal oxidative cleavage of alkenes, generating the respective carbonyl compounds. Additionally, it was demonstrated that this dioxazolidine intermediate could be reduced at low temperatures to the corresponding diols instead of fragmenting to the cleavage products.^16a,17b^ We envisioned that we could leverage the oxygen atom transfer chemistry of photoexcited nitroarenes for the Wacker-type oxidation of alkenes by biasing the fragmentation of the dioxazolidine intermediate. Additionally, with the precedent for high AM selectivity with palladium nitro complexes, we hypothesized that the carbonyl products could potentially be generated with high AM selectivity with photoexcited nitroarenes.^10−13^ Herein, we report a method for the oxidation of alkenes to carbonyls and acetonides using photoexcited nitroarenes and an iron Lewis acid catalyst (Scheme 1E). The system features anti-Markovnikov selectivity, resulting in the oxidation of the less substituted carbon for access to aldehyde and ketone products. Additionally, it was determined that both terminal and internal alkenes could be oxidized to acetonides when acetone was used as a co-solvent, generating protected 1,2-diols in a single step (vide infra).

After an exhaustive optimization campaign (see the Supporting Information), we found that the irradiation at 390 nm of a solution comprised of electron-deficient nitroarene **3** and **1a** in 1,2-dichloroethane at −30 °C for 24 h, followed by the addition of 10 mol % Fe(OTf)_2_ as a Lewis acid catalyst in the dark, generated a 65% yield of AM Wacker oxidation product **2a** after facile acetal protection due to the volatility of the formed aldehyde product. Investigations uncovered that the addition of a Lewis acid to the dioxazolidine intermediate of alkene **1a** at low temperatures was successful in suppressing the retrocycloaddition that results in oxidative cleavage and instead promoted fragmentation to the alkene oxidation product with exclusive AM selectivity.

With the optimized conditions in hand, we next investigated the scope of the transformation (Table 1). Simple, monosubstituted alkenes were oxidized to the corresponding acetal-protected aldehyde products (**2b**–**f**). Notably, a substrate containing an oxidatively sensitive boronic acid ester substituent, which would not be compatible with the oxidants employed under Tsuji–Wacker or epoxidation conditions, was converted into the protected aldehyde product in 23% yield (**2e**). 1,1-Disubstituted alkenes were also successfully oxidized to their corresponding aldehyde products in moderate to good yields (**2g**–**n**). Substrates with substituents containing Lewis basic sites such as esters (**2g** and **2h**), protected amines (**2j** and **2k**), and a nitrile (**2i**) were all tolerated under the reaction conditions, requiring only a slightly higher loading of the Lewis acid catalyst in some cases to maintain the desired reactivity. However, more strongly Lewis basic groups such as alcohols and free amines proved to be incompatible with the reaction conditions, and none of the desired AM Wacker oxidation products were detected, instead producing only the products of oxidative cleavage even with excess Lewis acid.^16a,17a^ This method also proved to be effective for generating ketone products with AM selectivity. The 1,2-disubstituted alkene cyclohexene was oxidized to protected ketone **2o** in moderate yield. When trisubstituted alkene **1p** was subjected to the reaction conditions, the product resulting from a hydride shift was found to be the major product, generating ketone **2p** in 54% yield. This preference for a hydride shift over an alkyl shift is in accordance with the results of the Meinwald rearrangement of the corresponding epoxide of **1p** (vide infra). Terpenes such as the protected citronellol (**1q**) and (*R*)-carvone (**1r**) were amenable to the reaction, generating the desired oxidation products **2q** and **2r**, respectively. Finally, we investigated the synthetic utility of the reaction on more complex natural products. Under the reaction conditions, β-caryophyllene was oxidized selectively at the trisubstituted double bond to aldehyde **2s** in 41% yield as a 4:1 mixture of diastereomers through a ring contraction via an alkyl migration. Additionally, betulin derivative **1t** was oxidized to aldehyde **2t** in a 38% yield, demonstrating the applicability of the reaction for late-stage functionalization of more complex alkenes.

**Table 1:**
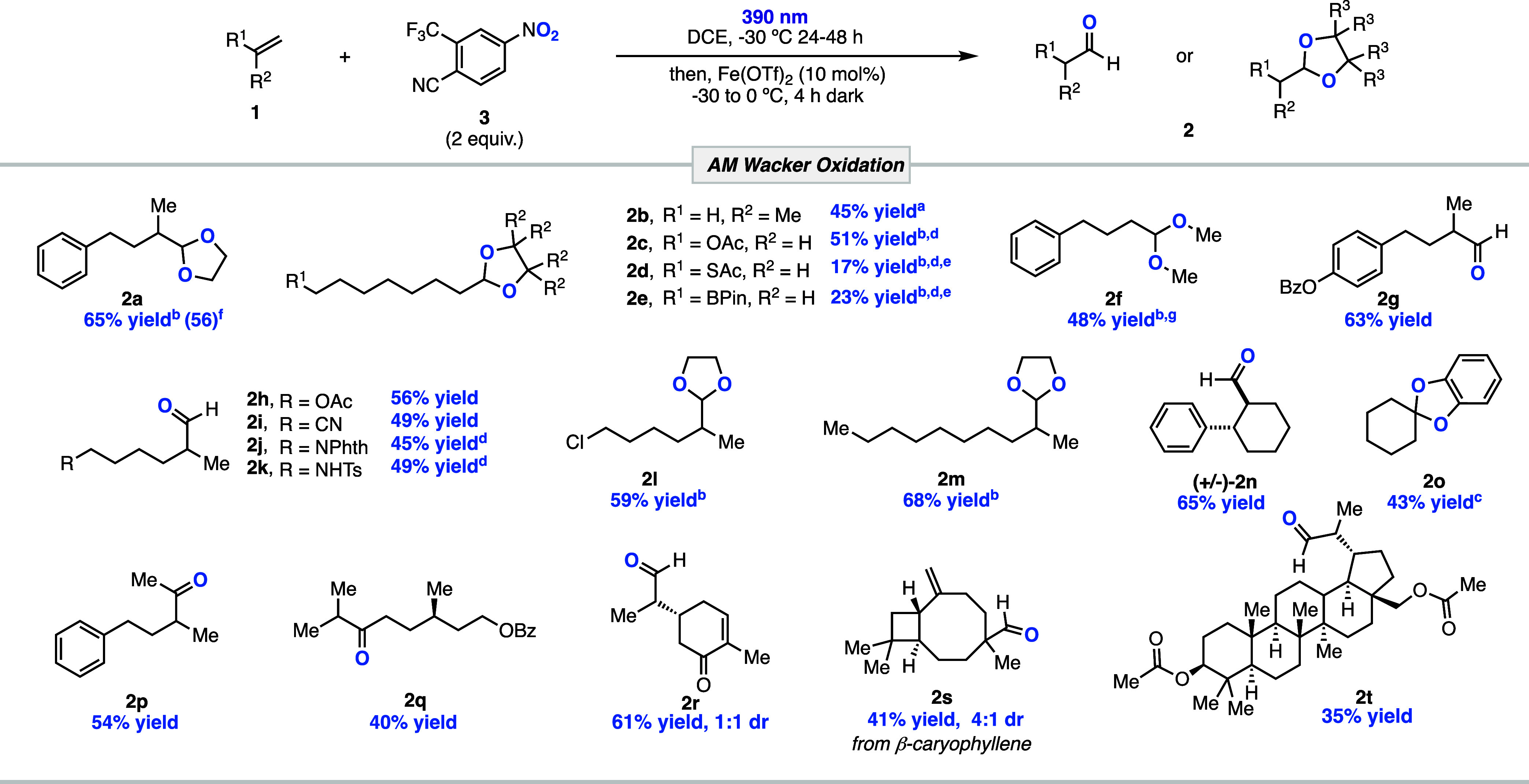
Scope of the Anti-Markovnikov Wacker Oxidation of Alkenes Using Photoexcited Nitroarenes^a^

a Isolated yields. With 5 equiv of pinacol at −30 °C.

b With 5 equiv of ethylene glycol at −30 °C.

c With 5 equiv of catechol at −30 °C.

d With 30 mol % Fe(OTf)_2_.

e ^1^H NMR yield.

f Reaction conducted on a 1 mmol scale.

g A 2.34:1 mixture with the oxidative cleavage product.

After examining the scope of the transformation, we next investigated the mechanism. Previous studies have established that visible light irradiation of a solution of nitroarenes and an alkene results in the formation of the 1,3,2-dioxazolidine intermediate, which can accumulate at cold temperatures.^17,18^ We hypothesize that the Lewis acid coordinates to the dioxazolidine intermediate to generate a Lewis acid–base complex, which fragments to generate the desired products via a 1,2-shift event. To support the latter step, deuterium labeling studies were performed with substrate **1a-***d*_2_ (Scheme 2A). Exposure of **1a-***d*_2_ to the reaction conditions resulted in 1,2-deuterium shift product **2a-***d*_2_ without a significant loss of deuterium incorporation. The presence of both 1,2-hydride shifts as well as 1,2-alkyl shifts is indicative of the potential formation of a carbocation intermediate. When comparing the ratio of hydride shift to alkyl shift between the Meinwald rearrangement of epoxide **4** with the ratio of alkene **1p** under the standard reaction conditions, we found them to be similar, thus suggesting the potential of a transient epoxide intermediate (Scheme 2B). Hence, we attempted to detect the formation of an epoxide by subjecting diadamantyl alkene **5** to the standard reaction conditions. We expected that if the mechanism goes through an epoxide intermediate, we should be able to detect epoxide **6** because the carbocation formation of **5** is not prone to rearrangements and **6** should not ring open under the standard reaction conditions.^20^ However, we did not detect or isolate any epoxide during the course of the reaction; only the product of oxidative cleavage was obtained (Scheme 2C). This suggests that the complex formed by the coordination of the Lewis acid to the dioxazolidine is the key intermediate en route to the desired products and that the ionization step is potentially reversible. Additionally, previous reports have demonstrated that intermediates similar to the dioxazolidine, such as cyclic phosphorane and cyclic sulfites, can undergo semipinacol rearrangements to generate carbonyls through both alkyl and hydride shifts.^20^ On the basis of our mechanistic studies and previous reports,^16a,17a^ we propose the following mechanism for the AM Wacker oxidation (Scheme 2D). Visible light irradiation of the nitroarene and alkene results in the formation of dioxazolidine **8**, followed by coordination of the Lewis acid to generate complex **9**. Ionization of the latter results in carbocation intermediate **10**, which rapidly undergoes a 1,2-hydride shift to generate the desired oxidized product **2**. Alternatively, intermediate **9** could directly generate **2** without going through intermediate **10** if rearrangement occurs in a concerted fashion similar to the rearrangements of cyclic sulfites and phosphoranes.^21^

**Scheme 2:**
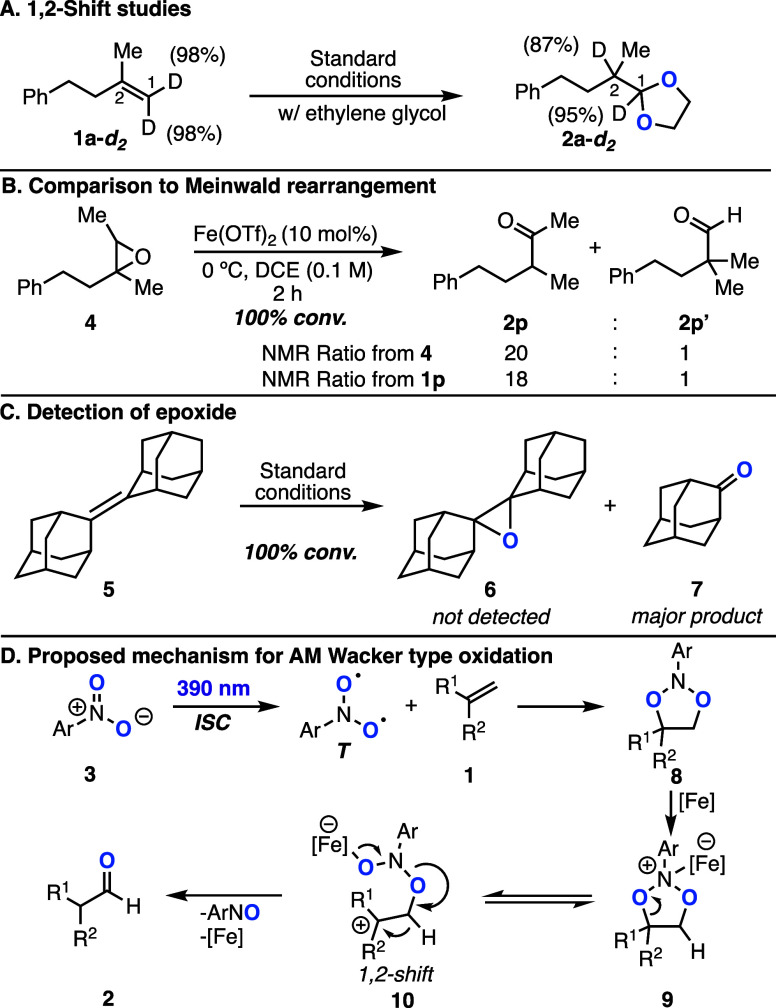
Mechanistic Studies and Proposed Mechanism

During the optimization of the AM Wacker oxidation (see the Supporting Information), we found that when acetone was employed as the solvent, one of the products of the reaction was an acetonide, equating to the protected 1,2-diol product of the alkene. Further investigations revealed that simply adding acetone along with the Lewis acid after the formation of the dioxazolidine intermediate can lead to the acetonide product selectively (Scheme 3A). Under these conditions, monosubstituted alkenes containing various functionalities were oxidized to their respective acetonide products in moderate to good yields (**11a**–**f**). Additionally, internal disubstituted alkenes, such as *trans*-5-decene and oleic acid methyl ester, were oxidized in good yields to acetonides **11g** and **11h**, respectively. For acetonide formation, we found that when protected allyl amino acid **12** was subjected to the standard reaction conditions, the expected acetonide product was not detected. Instead, seven-membered ring product **13** was isolated in 29% yield from the reaction mixture (Scheme 3B). The formation of this product supports an S_N_2-type mechanism, in which acetone adds to the less substituted carbon of the formed Lewis acid–base dioxazolidine complex. On the basis of this result, we propose the following mechanism for the generation of acetonides (Scheme 3C). Irradiation of the nitroarene and alkene generates dioxazolidine **14**, followed by coordination of a Lewis acid to **14** to generate complex **15**. Nucleophilic attack of acetone on the less substituted carbon of complex **15** generates intermediate **16**. Loss of nitrosoarene and ring closure of the oxocarbenium ion generate the desired acetonide **11**.

**Scheme 3:**
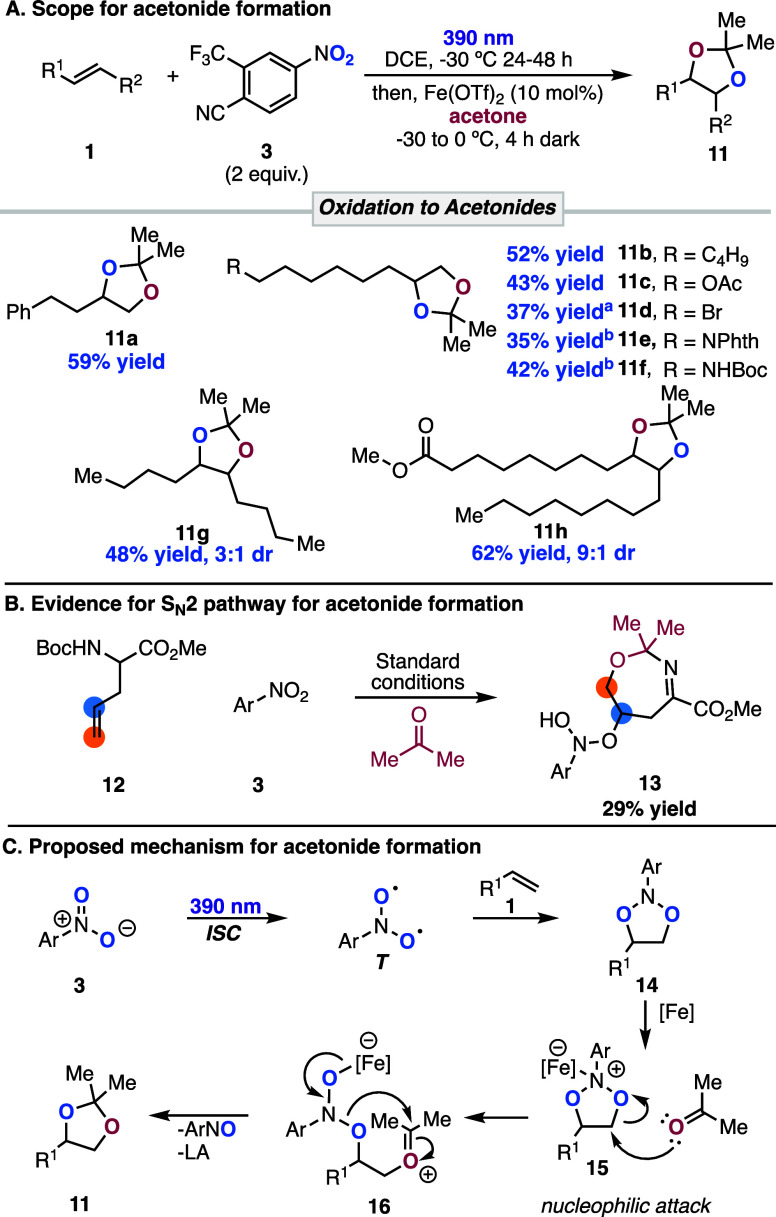
Acetonide Scope and Mechanism (A) Scope of acetonide formation promoted by photoexcited nitroarenes. See the Supporting Information for full experimental conditions. ^a^Isolated as a diol. ^b^With 30 mol % Fe(OTf)_2_. (B) Generation and isolation of a seven-membered ring intermediate formed by nucleophilic attack of acetone on the dioxazolidine intermediate. (C) Proposed mechanism for the oxidation of alkenes to acetonides by photoexcited nitroarenes.

In summary, we have reported a method for the oxidation of alkenes to carbonyls and acetonides using photoexcited nitroarenes and Lewis acid catalysis.^22^ The mild conditions of the transformations allow for the oxidation of alkenes with a wide range of substituents. Notably, coordination of a Lewis acid to the dioxazolidine generates a Lewis acid–base complex, which can undergo a semipinacol-type rearrangement to deliver carbonyl products with anti-Markovnikov selectivity. Additionally, this Lewis acid–base complex is susceptible to nucleophilic attack by acetone to generate acetonides as an alternative product. This work demonstrates that the dioxazolidine intermediate formed by the reaction of photoexcited nitroarenes and alkenes has the capability to act as a versatile intermediate for the oxidative functionalization of alkenes.

## Data Availability

The data underlying this study are available in the published article and its Supporting Information.

## Supporting Information Available

Experimental details, optimization studies, characterization data, and NMR spectra (PDF)
