# Supplementary material for: Merging Photoexcited Nitroarenes with Lewis Acid Catalysis for the Anti-Markovnikov Oxidation of Alkenes
Source: Org Lett. 2025 Feb 20;27(8):2011–5. doi: 10.1021/acs.orglett.5c00389 (PMC11877499; doi:10.1021/acs.orglett.5c00389)
Supplement: Supplementary file 1 — ol5c00389_si_001.pdf [file ol5c00389_si_001.pdf]

Merging Photoexcited Nitroarenes with Lewis Acid Catalysis for the Anti-Markovnikov Oxidation of Alkenes

Joshua M. Paolillo, Mahmoud R. Saleh, Ethan W. Junk and Marvin Parasram*

Department of Chemistry, New York University, 24 Waverly Place, 3rd floor, New York, NY 10003

Supporting Information

General information	1
Optimization of reaction conditions	2
General procedures	5
Substrate synthesis	7
Characterization of oxidized products	10
Mechanism studies	20
Comparison to Meinwald Rearrangement	20
Detection of potential reaction intermediates	22
Isolation of S_N2 product	24
NMR spectra	31
References	64

General information

All reactions were carried out in oven-dried glassware under a nitrogen atmosphere, unless otherwise stated. Photochemical reactions were cooled in an ethanol bath that was chilled using a Julabo FT902 immersion cooler. Solvents were dried and deoxygenated by passing through alumina in a solvent purification system. Dry DCE was acquired from Sigma Aldrich. $CDCl_3$ was purchased from Cambridge Isotope Laboratories. All alkene substrates were purchased from commercial sources, unless otherwise noted, and used without further purification.

All NMR spectra (^1H, ^{13}C, and ^{19}F) were recorded on Bruker 400 MHz; 500 MHz Avance spectrometers. The chemical shifts (δ) are given in parts per million and referenced to residual solvent peaks. Coupling constants (J) are reported in Hertz (Hz) to the nearest 0.1 Hz. The following multiplicity abbreviations are used: s singlet, d doublet, t triplet, m multiplet. Structural assignments were made with additional information from gCOSY, gHSQC, and gHMBC experiments. GC chromatograms were taken on an Agilent 8890 GC with 5977B MSD, and helium as the carrier gas. High-resolution mass spectra (HRMS) were obtained on a Waters ACQUITY/Xevo G3 LC-QToF MS which was acquired through the support of New York University. We utilized 34 W Kessil Lamps for our photochemical setups.

Optimization of reaction conditions

Table S1: Lewis acid, and Lewis acid loading screen

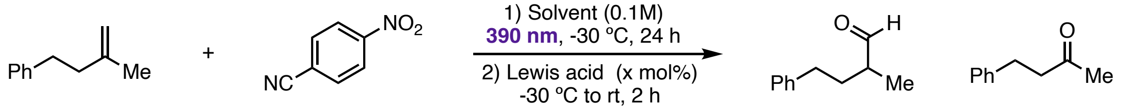

Entry	Solvent	Lewis acid (LA)	LA Mol %	^{1}H NMR Yield **2a** (%)	^{1}H NMR Yield **2a'** (%)
2	MeCN	$Fe(OTf)_2$	200	44	15
3	MeCN	$Fe(OTf)_2$	100	41	18
4	MeCN	$Fe(OTf)_2$	50	43	12
5	MeCN	$Fe(OTf)_2$	20	42	13
**6**	**MeCN**	**$Fe(OTf)_2$**	**10**	**43**	**19**
7	MeCN	$BF_3 \cdot Et_2O$	10	0	68
8	MeCN	$Cu(OTf)_2$	10	0	80
9	MeCN	$Mg(OTf)_2$	10	20	38
10	MeCN	$Fe(OTf)_3$	10	38	16
11	MeCN	$Sc(OTf)_3$	10	40	18
12	MeCN	$BiCl_3$	10	0	69
13	MeCN	$Eu(OTf)_3$	10	16	43
14	MeCN	$Ga(OTf)_3$	10	22	46
15	MeCN	$La(OH)_3$	10	0	71
16	MeCN	$SnCl_4$	200	23	25

Reactions were performed on a 0.2 mmol scale. ^{1}H NMR yields calculated using CH_2Br_2 as an external standard.

A selection of nitroarenes that have previously been shown to form high quantities of dioxazolidine intermediates at cold temperatures with alkenes while under irradiation were explored to increase the selectivity for the AM oxidation products through suppression of the formation of the product of oxidative cleavage.[2]

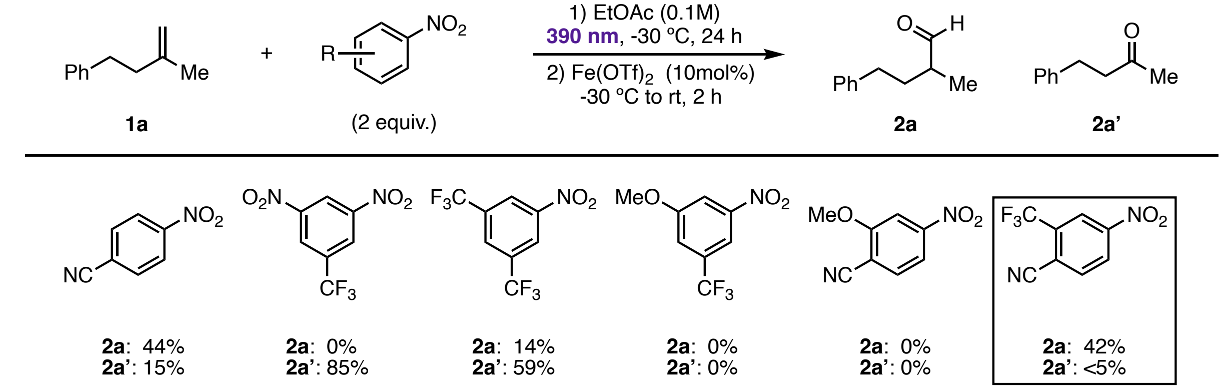

Figure S1: Nitroarene screen for suppression of oxidative cleave product. Reactions were performed on a 0.2 mmol scale and the yields of **2a** and **2a'** were determined by ^{1}H NMR using CH_2Br_2 as an external standard.

Table S2: Solvent Screen

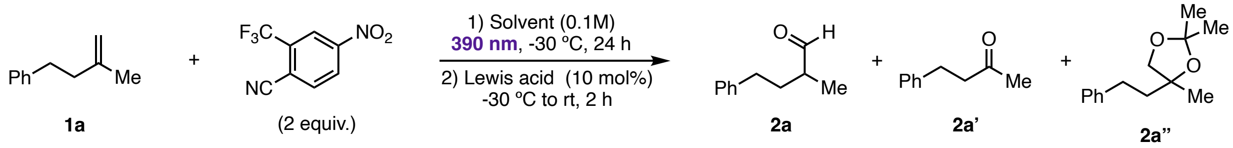

Entry	Solvent	^{1}H NMR Yield **2a** (%)	^{1}H NMR Yield **2a'** (%)	^{1}H NMR Yield **2a"** (%)
1	Acetone	44	15	26
2	$MeNO_2$	0	65	0
3	HFIP	0	50	0
4	EtOAc	48	5	0
**5**	**DCE**	**68**	**2**	**0**

Reactions were performed on a 0.2 mmol scale. ^{1}H NMR yields calculated using CH_2Br_2 as an external standard.

Table S3: Quench temperature screening

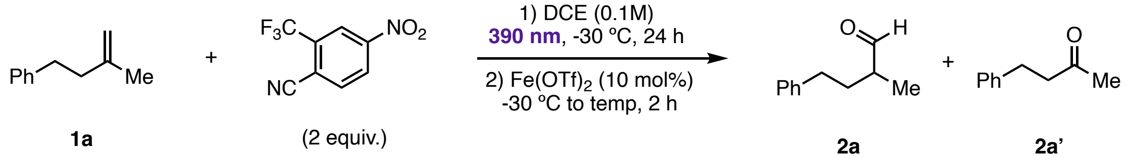

Entry	Quench Temperature (°C)	^{1}H NMR Yield **2a** (%)	^{1}H NMR Yield **2a'** (%)
1	-30	0	75
2	-20	0	72
3	-10	15	34
4	0	42	<5

Reactions were performed on a 0.2 mmol scale. ^{1}H NMR yields calculated using CH_2Br_2 as an external standard.

Table S4: Acetone screening for the formation of acetonides

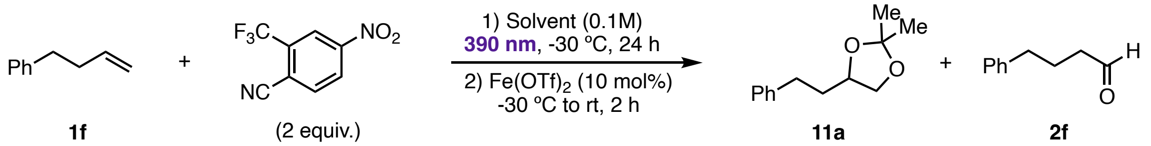

Entry	Solvent	^{1}H NMR Yield **3a** (%)	^{1}H NMR Yield **2f** (%)
1	Acetone	45	0
2	1:1 DCE/Acetone	48	0
3	3:1 DCE/Acetone	47	0
4	5:1 DCE/acetone	50	0

Reactions were performed on a 0.2 mmol scale. ^{1}H NMR yields calculated using CH_2Br_2 as an external standard.

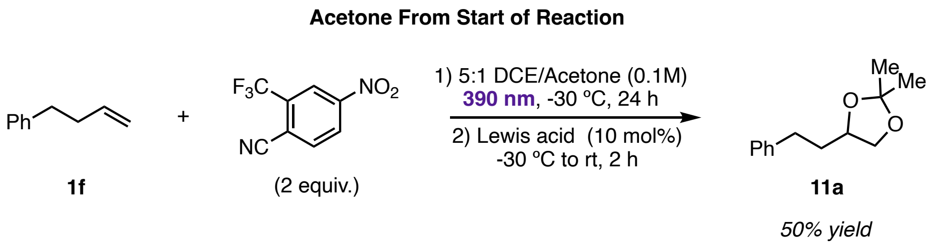

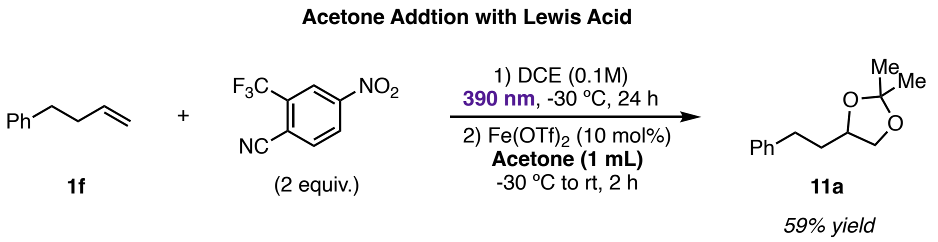

Figure S2: Addition of acetone from the start of the reaction compared to the addition of acetone during addition of Lewis acid. Reactions were performed on a 0.2 mmol scale and the yields of **3a** were determined by ^{1}H NMR using CH_2Br_2 as an external standard.

General procedures

General Procedure A: Standard Procedure for AM Wacker Oxidation of Alkenes to Aldehydes and Ketones

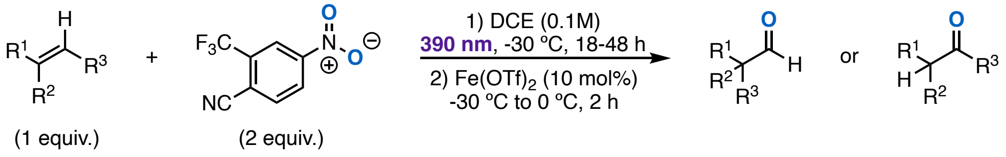

Alkene (0.5 mmol, 1 equiv.) and 4-nitro-2-(trifluoromethyl)benzonitrile (216 mg, 1 mmol, 2 equiv.) were added to a 4 dram vial (flame or oven dried) equipped with a stir bar. The reaction vial was then purged with N_2 flow for 15 min followed by the addition of dichloroethane (5 mL, 0.1 M). The vial was cooled to -30 ºC using an immersion cooler and was irradiated with 390 nm LED lamp under constant stirring (stir rate 700 rpm) for 18 hours or until complete consumption of the alkene as determined by GCMS analysis. Upon complete consumption of the alkene the lamp was turned off and the cap of the vial was removed.

For aldehyde and ketone products, while open to air, $Fe(OTf)_2$ (17.7 mg, 0.05 mmol, 10 mol%) was added at – 30 ºC. The cap was replaced, and the mixture was allowed to stir while slowly warming to 0 ºC over 2 hours. DI H_2O (45.1 µL, 2.5 mmol, 5 equiv.) was then added to quench the Lewis acid and the reaction mixture was transferred to a heating block, where it was heated to 60 ºC for 2 hours. The mixture was cooled to room temperature and then filtered through a plug of silica using dichloromethane or ethyl acetate as an eluent. The solvent was removed *in vacuo* and the crude product was isolated by column chromatography to afford the desired product.

For acetals and ketal products, while open to air $Fe(OTf)_2$ (17.7 mg, 0.05 mmol, 10 mol%) and the corresponding diol (5 equiv.) were added at -30 ºC. The cap was replaced, and the mixture was allowed to stir while slowly warming to 0 ºC over 2 hours. DI H_2O (45.1 µL, 2.5 mmol, 5 equiv.) was then added to quench the Lewis acid and the reaction mixture was transferred to a heating block, where it was heated to 60 ºC for 2 hours. The mixture was cooled to room temperature and then filtered through a plug of silica using dichloromethane or ethyl acetate as an eluent. The solvent was removed *in vacuo* and the crude product was isolated by column chromatography to afford the desired product.

General Procedure B: Standard Procedure for Oxidation of Alkene to Acetonides

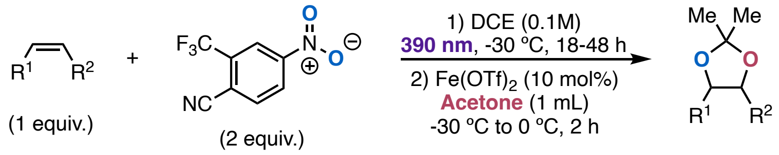

Alkene (0.5 mmol, 1 equiv.) and 4-nitro-2-(trifluoromethyl)benzonitrile (216 mg, 1 mmol, 2 equiv.) were added to a 4 dram vial (flame or oven dried) equipped with a stir bar. The reaction vial was then purged with N_2 flow for 15 min followed by the addition of dichloroethane (5 mL, 0.1 M). The vial was cooled to -30 ºC using an immersion cooler and was irradiated with 390 nm LED lamp under constant stirring (stir rate 700 rpm) for 18 hours or until complete consumption of the alkene as determined by GCMS analysis. Upon complete consumption of the alkene the lamp was turned off, the cap of the vial was removed and, while open to air, 1 mL of cold acetone (-30 ºC) and $Fe(OTf)_2$ (17.7 mg, 0.05 mmol, 10 mol%) were added at -30 ºC. The cap was replaced, and the mixture was allowed to stir while slowly warming to 0 ºC over 2 hours. The reaction mixture was then transferred to a heating block, where it was heated to 60 ºC for 2 hours. The mixture was cooled to room temperature and then filtered through a plug of silica using dichloromethane or ethyl acetate as an eluent. The solvent was removed *in vacuo* and the crude product was isolated by column chromatography to afford the desired product.

Scale up procedure

(3-methylbut-3-en-1-yl)benzene (146.2 mg, 1.0 mmol, 1 equiv.) and 4-nitro-2-(trifluoromethyl)benzonitrile (432.2 mg, 1 mmol, 2 equiv.) were added to a flame dried Schlenk tube equipped with a stir bar. The Schlenk tube was sealed with a septum and evacuated and refilled with nitrogen, followed by the addition of dichloroethane (10 mL, 0.1 M). The mixture was cooled to -30 ºC using an immersion cooler and was irradiated with 390 nm LED lamp under constant stirring (stir rate 700 rpm) for 18 hours. The lamp was turned off and the septum was removed. $Fe(OTf)_2$ (35.40 mg, 0.10 mmol, 0.1 equiv.) and ethylene glycol (278.8 µL, 5.0 mmol, 5 equiv.) under air were added and the Schlenk tube was resealed. The mixture was warmed to room temperature over 2 hours, after which it was placed in an oil bath and heated to 60 ºC for 2 hours. The mixture was cooled to room temperature and passed through a plug of silica, eluting with DCM. The solvent was removed under reduced pressure and the crude product was isolated via flash chromatography (0 – 5% EtOAc in hexanes) to afford the pure product as a colorless oil (115 mg, 56% yield).

Substrate synthesis

Literature Reported

(3-methylbut-3-en-1-yl)benzene (**1a**)[1], oct-7-en-1-yl acetate (**1c**)[2], *S*-(oct-7-en-1-yl) ethanethioate (**1d**)[2], 4,4,5,5-tetramethyl-2-(oct-7-en-1-yl)-1,3,2-dioxaborolane (**1e**)[2], 6-methylhept-6-enenitrile (**1i**)[3], 2-methyldec-1-ene (**1m**)[4], (2-methylenecyclohexyl)benzene (**1n**)[5], (3-methylpent-3-en-1-yl)benzene (**1p**)[6], betulin diacetate (**1t**)[7], 2-(oct-7-en-1-yl)isoindoline-1,3-dione (**1e'**)[2], *tert*-butyl oct-7-en-1-ylcarbamate (**1f'**)[2], and (3-methylbut-3-en-1-yl-4,4-d_2)benzene (**1a-d$_2$**)[23] were synthesized according to literature procedure.

Substrate Synthesis

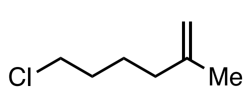

6-chloro-2-methylhex-1-ene (1l)

To a flame dried flask equipped with a stir bar was added methyltriphenylphosphonium bromide (17.1g, 1.2 equiv., 48 mmol) and THF (150 mL). The mixture was cooled to 0 ºC in an ice bath and then potassium *tert*-butoxide (8.98 g, 2 equiv., 80 mmol) was added in portions. The mixture was stirred for 15 min at 0 ºC, followed by the dropwise addition of 6-chloro-2-hexanone (5.38g, 5.28 mL, 1 equiv., 40.0 mmol). The mixture was stirred at 0 ºC for 1 hour and then warmed to room temperature and progress of the reaction was monitored by GCMS or TLC. Upon completion of the reaction, the mixture was quenched by the addition of water (100 mL). The mixture was transferred to a separatory funnel and diluted with an additional 100 mL of water. The mixture was extracted with DCM (3 x 100 mL). The combined organic layers were washed with water (3 x 75 mL), then brine (150 mL), and then dried over sodium sulfate. The solvent was removed under reduced pressure and to afford the crude product. The crude product was filtered through a plug of silica, eluting with hexanes, to give the pure product as a colorless oil (2.65 g, 50% yield). All spectra were in accordance with literature reported spectra.[8]

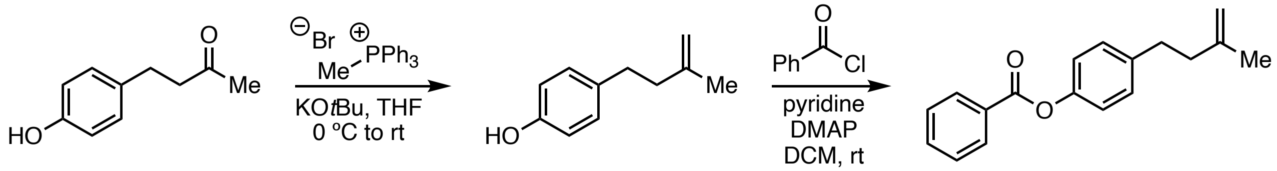

4-(3-methylbut-3-en-1-yl)phenyl benzoate (1g)

To a flame dried round bottom flask equipped with a stir bar was added methyltriphenylphosphonium bromide (8.57 g, 1.2 equiv., 24 mmol) and THF (50 mL). The mixture was cooled to 0 ºC in an ice bath and then potassium *tert*-butoxide (5.61 g, 2.5 equiv., 50 mmol) was added in portions. The mixture was stirred for 15 min at 0 ºC, followed by the dropwise addition 4-(4-hydroxyphenyl)butan-2-one (3.28 g, 1 equiv., 20.0 mmol) dissolved in 25 mL of THF. The mixture was stirred at 0 ºC for 1 hour and then was slowly warmed to room temperature. The mixture was quenched by the addition of water (100 mL) and transferred to a separatory funnel. The mixture was extracted with Et_2O (3 x 75 mL). The combined organic layers were washed with water (3 x 75 mL). The organic layer was then washed with 2 M NaOH solution (3 x 30 mL). The pH of the aqueous layer was adjusted to 1 using 6 M HCl solution and extracted with Et_2O (3 x 50 mL). The organic layer was washed with water (50 mL), then once with brine (75

mL), and then dried over sodium sulfate. The solvent was removed *in vacuo* to afford crude 4-(3-methylbut-3-en-1-yl)phenol as a viscous light-yellow oil (1.75 g, 54% yield), which was used directly in the next step without further purification.

To a flame dried flask equipped with a stir bar was added crude 4-(3-methylbut-3-en-1-yl)phenol (985 mg, 1 equiv. 6.00 mmol), pyridine (1.04 g, 1.07 mL, 13.2 mmol), DMAP (88.0 mg, 0.12 equiv., 0.720 mmol) and DCM (30 mL). Then benzoyl chloride (928 mg, 764 µL, 1.1 equiv., 6.60 mmol) was added dropwise. The solution was stirred at room temperature and monitored by TLC. Upon completion of the reaction, the mixture was quenched by the addition of water (75 mL). The mixture was transferred to a separatory funnel and diluted with DCM (30 mL). The layers were separated, and the organic layer was washed with saturated sodium bicarbonate (2 x 75 mL) and then with brine (75 mL). The organic layer was dried over sodium sulfate and the solvent was removed *in vacuo* to afford the crude product, which was purified by recrystallization out of EtOAc to afford **1g** as a white crystalline solid (0.74 g, 46% yield)

^{1}H NMR (500 MHz, $CDCl_3$) (δ, ppm): 8.24 – 8.19 (m, 2H), 7.68 – 7.60 (m, 1H), 7.52 (t, *J* = 7.8 Hz, 2H), 7.29 – 7.23 (m, 2H), 7.17 – 7.11 (m, 2H), 4.79 – 4.72 (m, 2H), 2.83 – 2.76 (m, 2H), 2.35 (dd, *J* = 9.0, 7.3 Hz, 2H), 1.82 – 1.78 (m, 3H).
^{13}C{H} NMR (126 MHz, $CDCl_3$) (δ, ppm): 165.4, 149.1, 145.3, 139.9, 133.6, 130.3, 129.8, 129.4, 128.7, 121.5, 110.5, 39.7, 33.8, 22.7.
HRMS (ESI TOF): calcd. for $C_{18}H_{19}O_2$ $[M+H]^+$ 267.1385, found 267.1384

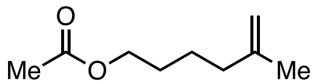

5-methylhex-5-en-1-yl acetate (1h)
To a flame dried round bottom flask equipped with a stir bar was added potassium acetate (740 mg, 2.00 equiv., 7.54 mmol) and DMF (20 mL). Then 6-chloro-2-methylhex-1-ene (**1l**) (500 mg, 1.00 equiv., 3.77 mmol) was added and the mixture was heated to 80 ºC for 18 hours. The mixture was cooled to room temperature and diluted with 50 mL of water. The mixture was transferred to a separatory funnel and extracted with diethyl ether (3 x 20 mL). The combined organic layers were washed with water (25 mL), then once with brine (25 mL), and then dried over sodium sulfate. The solvent was removed under reduced pressure to afford the crude product which was purified by column chromatography (97:3 hexanes/EtOAc) to give the pure product **1g** as a colorless oil (368 mg, 63% yield).

^{1}H NMR (400 MHz, $CDCl_3$) (δ, ppm): 4.73-4.59 (m, 2H), 4.04 (t, *J* = 6.6 Hz, 2H), 2.05 – 1.97 (m, 5H), 1.68 (t, *J* = 1.1 Hz, 3H), 1.66 – 1.54 (m, 2H), 1.55 – 1.32 (m, 2H).
^{13}C{H} NMR (101 MHz, $CDCl_3$) (δ, ppm): 171.2, 145.4, 110.2, 64.5, 37.3, 28.2, 23.9, 22.3, 21.0.
HRMS (ESI TOF): calcd. for $C_9H_{17}O_2$ $[M+H]^+$ 157.1229, found 157.1226

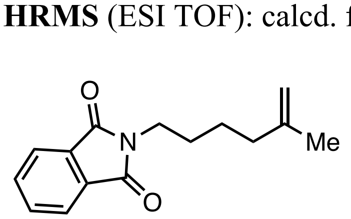

2-(5-methylhex-5-en-1-yl)isoindoline-1,3-dione (1j)

To a round bottom flask equipped with a stir bar was added potassium phthalimide (2.09 g, 1.5 equiv., 11.3 mmol) and DMF (15 mL). 6-chloro-2-methylhex-1-ene (**1l**) was then added to the reaction mixture. The mixture was heated to 110 ºC and allowed to stir overnight. Upon completion of the reaction as determined by GCMS or TLC, the reaction was cooled to room temperature. The mixture was the poured into 75 mL of ice water slurry and stirred for 30 min. The mixture was transferred to a separatory funnel and extracted with EtOAc (3 x 50 mL). The combined organic layers were washed with water (3 x 75 mL), then with 1M NaOH solution (2 x 75 mL), then once with brine, and then were dried over sodium sulfate. The solvent was removed *in vacuo* to afford the product **1j** as a white solid (1.2 g, 69% yield) which was used without further purification.

^{1}H NMR (400 MHz, $CDCl_3$) (δ, ppm): 7.89 – 7.79 (m, 2H), 7.75 – 7.67 (m, 2H), 4.73 – 4.64 (m, 2H), 3.69 (t, *J* = 7.3 Hz, 2H), 2.05 (td, *J* = 7.6, 1.2 Hz, 2H), 1.74 – 1.62 (m, 5H), 1.55 – 1.42 (m, 2H).
^{13}C{H} NMR (101 MHz, $CDCl_3$) (δ, ppm): 168.6, 145.5, 134.0, 132.3, 123.3, 110.4, 38.0, 37.4, 28.3, 24.9, 22.4.
HRMS (ESI TOF): calcd. for $C_{15}H_{18}NO_2$ $[M+H]^+$ 244.1338, found 244.1337

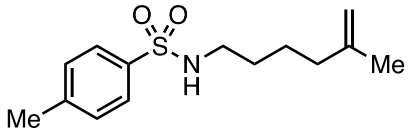

4-methyl-*N*-(5-methylhex-5-en-1-yl)benzenesulfonamide (1k)
To a flame dried flask equipped with a stir bar was added 6-chloro-2-methylhex-1-ene (**1l**) (500 mg, 1 equiv., 3.77 mmol), potassium carbonate (1.04 g, 2 equiv., 7.54 mmol), potassium bromide (897 mg, 2 equiv., 7.54 mmol), toluene sulfonamide (1.29 g, 2 equiv., 7.54 mmol) and MeCN (15 mL). The mixture was heated to reflux and monitored by TLC and GCMS. Upon completion of the reaction (24 h), the mixture was cooled to room temperature and diluted with Et_2O (75 mL). The mixture was passed through a filter of Celite® and transferred to a separatory funnel. The mixture was washed with saturated sodium thiosulfate solution (50 mL) and the aqueous layer was extracted with Et_2O (3 x 50 mL). The combined organic layers were washed with brine then dried over sodium sulfate. The solvent was removed *in vacuo* to afford the crude product, which was isolated by column chromatography (85:15 hexanes/EtOAc) to give the pure product was a light tan solid (415 mg, 41% yield).

^{1}H NMR (400 MHz, $CDCl_3$) (δ, ppm): 7.75 (d, *J* = 8.3 Hz, 2H), 7.29 (d, *J* = 8.1 Hz, 2H), 4.91 (t, *J* = 6.2 Hz, 1H), 4.70 – 4.46 (m, 2H), 2.92 (q, *J* = 6.5 Hz, 2H), 2.41 (s, 3H), 1.91 (t, *J* = 7.1 Hz, 2H), 1.63 (t, *J* = 1.1 Hz, 3H), 1.50 – 1.31 (m, 4H).
^{13}C{H} NMR (101 MHz, $CDCl_3$) δ 145.3, 143.4, 137.1, 129.8, 127.2, 110.2, 43.2, 37.2, 29.2, 24.5, 22.3, 21.6.
HRMS (ESI TOF): calcd. for $C_{14}H_{22}NO_2S$ $[M+H]^+$ 268.1371, found 268.1375

Characterization of oxidized products

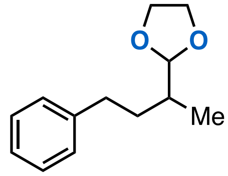

2-(4-phenylbutan-2-yl)-1,3-dioxolane (2a)
Prepared according to general procedure A using ethylene glycol. The title compound was isolated via flash chromatography (gradient 0 – 5% EtOAc/hexanes) as a colorless oil (67 mg, 65% yield).

TLC (SiO_2) R_f = 0.45 in 95:5 hexanes/EtOAc
^{1}H NMR (500 MHz, $CDCl_3$) (δ, ppm): 7.28 – 7.23 (m, 2H), 7.20 – 7.11 (m, 3H), 4.69 (d, J = 4.3 Hz, 1H), 3.94 – 3.88 (m, 2H), 3.84 – 3.81 (m, 2H), 2.72 (ddd, J = 13.5, 10.6, 5.3 Hz, 1H), 2.57 (ddd, J = 13.7, 10.4, 6.4 Hz, 1H), 1.86 (dddd, J = 13.6, 10.7, 6.3, 4.5 Hz, 1H), 1.75 (ddt, J = 8.7, 6.6, 4.3 Hz, 1H), 1.47 (dddd, J = 13.6, 10.3, 8.9, 5.3 Hz, 1H), 0.98 (d, J = 6.9 Hz, 3H).
^{13}C{H} NMR (126 MHz, $CDCl_3$) (δ, ppm): 142.7, 128.5, 128.4, 125.8, 107.7, 65.2, 65.1, 36.6, 33.5, 33.3, 14.0.
HRMS (ESI TOF): calcd. for $C_{13}H_{19}O_2$ $[M+H]^+$ 207.1385, found 207.1378

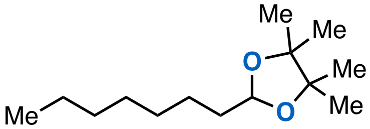

2-heptyl-4,4,5,5-tetramethyl-1,3-dioxolane (2b)
Prepared according to general procedure A using pinacol. The title compound was isolated via flash chromatography (gradient 0 – 5% EtOAc/hexanes) as a colorless oil (51 mg, 45% yield). All analytical data for **2b** was in accordance with literature data.[9]

TLC (SiO_2) R_f = 0.49 in 95:5 hexanes/EtOAc
^{1}H NMR (400 MHz, $CDCl_3$) (δ, ppm): 5.02 (t, J = 5.1 Hz, 1H), 1.61 – 1.53 (m, 2H), 1.46 – 1.24 (m, 10H), 1.19 (s, 12H), 0.94 – 0.83 (m, 3H).
^{13}C{H} NMR (101 MHz, $CDCl_3$) (δ, ppm): 101.1, 81.7, 36.6, 31.9, 29.8, 29.5, 24.6, 24.4, 22.7, 22.2, 14.2.

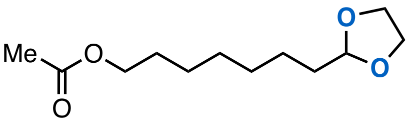

7-(1,3-dioxolan-2-yl)heptyl acetate (2c)
Prepared according to general procedure A using ethylene glycol. The title compound was isolated via flash chromatography (gradient 5 – 20% EtOAc/hexanes) as a light-yellow oil (59 mg, 51% yield).

TLC (SiO_2) R_f = 0.24 in 9:1 hexanes/EtOAc
^{1}H NMR (500 MHz, $CDCl_3$) (δ, ppm): 4.84 (s, 1H), 4.04 (t, J = 6.8 Hz, 2H), 3.98 – 3.91 (m, 2H), 3.91 – 3.79 (m, 2H), 2.04 (s, 3H), 1.69 – 1.56 (m, 5H), 1.43 – 1.29 (m, 7H).

^{13}C{H} NMR (126 MHz, $CDCl_3$) (δ, ppm): 171.4, 104.7, 65.0, 64.7, 34.0, 29.5, 29.3, 28.7, 25.9, 24.1, 21.2.
HRMS (ESI TOF): calcd. for $C_{12}H_{22}O_4Na$ $[M+Na]^+$ 253.1416, found 253.1417

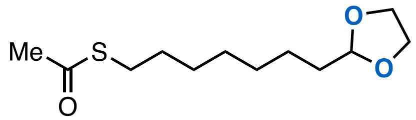

***S*-(7-(1,3-dioxolan-2-yl)heptyl) ethanethioate (2d)**
Prepared according to general procedure A using ethylene glycol. ^{1}H NMR yield determined using CH_2Br_2 as an external standard (yield = 17%).

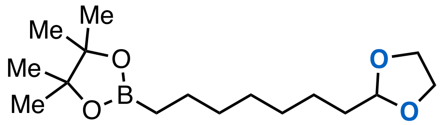

2-(7-(1,3-dioxolan-2-yl)heptyl)-4,4,5,5-tetramethyl-1,3,2-dioxaborolane (2e)
Prepared according to general procedure A using ethylene glycol. ^{1}H NMR yield determined using CH_2Br_2 as an external standard (yield = 23%).

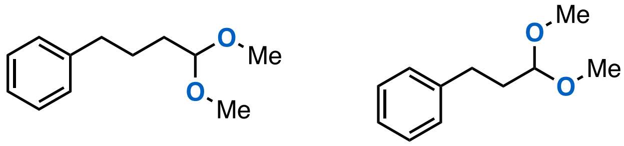

(4,4-dimethoxybutyl)benzene (2f) and (3,3-dimethoxypropyl)benzene (2f')
Prepared according to general procedure A using methanol. The title compounds were isolated as a 7:3 mixture of **2f** and **2f'**via flash chromatography (0 – 10% EtOAc/hexanes) as a colorless oil (47 mg, 48% yield). All analytical data for **2f** and **2f'** was in accordance with literature data.[10,24]

2f
TLC (SiO_2) R_f = 0.18 in 97:3 hexanes/EtOAc
^{1}H NMR (500 MHz, $CDCl_3$) δ 7.35 – 7.25 (m, 2H), 7.19 (t, J = 7.9 Hz, 3H), 4.37 (t, J = 5.5 Hz, 1H), 3.30 (s, 6H), 2.64 (t, J = 7.3 Hz, 2H), 1.72 – 1.60 (m, 4H).
^{13}C{H} NMR (126 MHz, $CDCl_3$) (δ, ppm): δ 142.3, 128.4, 126.1, 125.9, 104.58, 52.8, 35.9, 32.2, 26.5.

2f'
TLC (SiO_2) R_f = 0.18 in 97:3 hexanes/EtOAc
^{1}H NMR (500 MHz, $CDCl_3$) δ 7.35 – 7.25 (m, 2H), 7.19 (t, J = 7.9 Hz, 3H), 4.37 (t, J = 5.5 Hz, 1H), 3.34 (s, 6H), 2.71 – 2.66 (m, 2H), 1.97 – 1.90 (m, 2H)
^{13}C{H} NMR (126 MHz, $CDCl_3$) (δ, ppm): δ 141.7, 128.6, 128.4, 126.0, 103.9, 52.9, 35.8, 31.0.

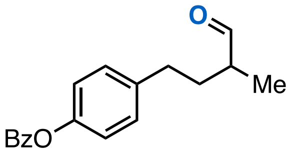

4-(3-methyl-4-oxobutyl)phenyl benzoate (2g)
Prepared according to general procedure A. The title compound was isolated via flash chromatography (gradient 0 – 20% EtOAc/hexanes) as a colorless solid (89 mg, 63% yield). (contains 8% carboxylic acid from oxidation by air after isolation)

TLC (SiO_2) R_f = 0.21 in 9:1 hexane/EtOAc
^{1}H NMR (500 MHz, $CDCl_3$) (δ, ppm): 9.65 (d, J = 1.9 Hz, 1H), 8.24 – 8.17 (m, 2H), 7.71 – 7.60 (m, 1H), 7.51 (t, J = 7.8 Hz, 2H), 7.27 – 7.22 (m, 2H), 7.17 – 7.12 (m, 2H), 2.69 (dt, J = 8.9, 6.1 Hz, 2H), 2.40 (pd, J = 6.9, 1.8 Hz, 1H), 2.15 – 2.05 (m, 1H), 1.69 (ddt, J = 13.6, 9.1, 6.8 Hz, 1H), 1.17 (d, J = 7.1 Hz, 3H).
^{13}C{H} NMR (126 MHz, $CDCl_3$) (δ, ppm): 204.8, 165.4, 149.4, 139.1, 133.7, 130.3, 129.7, 129.5, 128.7, 121.8, 45.7, 32.6, 32.2, 13.5.
HRMS (ESI TOF): calcd. for $C_{18}H_{18}O_3Na$ $[M+Na]^+$ 305.1154, found 305.1147

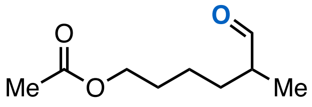

5-methyl-6-oxohexyl acetate (2h)
Prepared according to general procedure A. The title compound was isolated via flash chromatography (gradient 0 –20% EtOAc/hexanes) as a light-yellow oil (48 mg, 56% yield). All analytical data for **2h** was in accordance with literature data.[11]

TLC (SiO_2) R_f = 0.12 in 88:12 hexane/EtOAc
^{1}H NMR (400 MHz, $CDCl_3$) (δ, ppm): 9.61 (d, J = 1.9 Hz, 1H), 4.06 (t, J = 6.6 Hz, 2H), 2.41 – 2.23 (m, 1H), 2.04 (s, 3H), 1.80 – 1.69 (m, 1H), 1.68 – 1.52 (m, 2H), 1.48 – 1.32 (m, 3H), 1.10 (d, J = 7.0 Hz, 3H).
^{13}C{H} NMR (101 MHz, $CDCl_3$) (δ, ppm): 205.0, 171.3, 64.3, 46.3, 30.2, 28.7, 23.5, 21.1, 13.5.

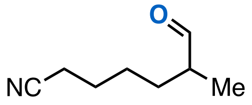

6-methyl-7-oxoheptanenitrile (2i)
Prepared according to general procedure A. The title compound was isolated via flash chromatography (gradient 5 – 30 % EtOAc/hexanes) as a colorless oil (34 mg, 49% yield).

TLC (SiO_2) R_f = 0.11 in 85:15 hexanes/EtOAc
^{1}H NMR (400 MHz, $CDCl_3$) (δ, ppm): 9.62 (d, J = 1.8 Hz, 1H), 2.43 – 2.29 (m, 3H), 1.82 – 1.62 (m, 3H), 1.55 – 1.44 (m, 2H), 1.45 – 1.31 (m, 1H), 1.13 (d, J = 7.1 Hz, 3H).
^{13}C{H} NMR (101 MHz, $CDCl_3$) (δ, ppm): 204.6, 119.6, 46.1, 29.6, 26.2, 25.5, 17.2, 13.5.
HRMS (ESI TOF): calcd. for $C_8H_{14}NO$ $[M]^+$ 140.1075, found 140.1076

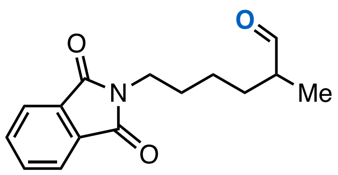

6-(1,3-dioxoisoindolin-2-yl)-2-methylhexanal (2j)
Prepared according to general procedure A using 30 mol% $Fe(OTf)_2$. The title compound was isolated via flash chromatography (gradient 10 – 20% EtOAc/hexanes) as a white solid (59 mg, 45% yield).

TLC (SiO_2) R_f = 0.14 in 9:1 hexane/EtOAc
^{1}H NMR (400 MHz, $CDCl_3$) (δ, ppm): 9.61 (d, J = 1.8 Hz, 1H), 7.84 (dd, J = 5.5, 3.1 Hz, 2H), 7.71 (dd, J = 5.5, 3.0 Hz, 2H), 3.69 (t, J = 7.2 Hz, 2H), 2.42 – 2.28 (m, 1H), 1.82 – 1.65 (m, 3H), 1.47 – 1.31 (m, 3H), 1.09 (d, J = 7.0 Hz, 3H).
^{13}C{H} NMR (101 MHz, $CDCl_3$) (δ, ppm): 205.0, 168.5, 134.0, 132.2, 123.3, 46.3, 37.7, 30.0, 28.7, 24.3, 13.4.
HRMS (ESI TOF): calcd. for $C_{15}H_{18}NO_3$ $[M+H]^+$ 260.1287, found 260.1285

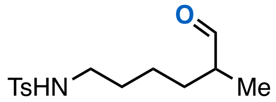

4-methyl-*N*-(5-methyl-6-oxohexyl)benzenesulfonamide (2k)
Prepared according to general procedure A using 30 mol% $Fe(OTf)_2$. The title compound was isolated via flash chromatography (20 – 30% EtOAc/hexanes) as a light yellow oil (69 mg, 49% yield).

TLC (SiO_2) R_f = 0.10 in 5:2 hexanes/EtOAc
^{1}H NMR (500 MHz, $CDCl_3$) (δ, ppm): 9.57 (d, J = 1.8 Hz, 1H), 7.74 (d, J = 8.3 Hz, 2H), 7.31 (d, J = 8.0 Hz, 2H), 4.45 (t, J = 6.3 Hz, 1H), 2.94 (q, J = 6.9 Hz, 2H), 2.43 (s, 3H), 2.28 (qd, J = 6.6, 1.7 Hz, 1H), 1.67 – 1.60 (m, 1H), 1.53 – 1.44 (m, 2H), 1.37 – 1.20 (m, 3H), 1.06 (d, J = 7.0 Hz, 3H).
^{13}C{H} NMR (126 MHz, $CDCl_3$) (δ, ppm): 204.9, 143.6, 137.1, 129.9, 127.2, 46.2, 43.0, 29.9, 29.7, 24.0, 21.6, 13.4.
HRMS (ESI TOF): calcd. for $C_{14}H_{22}NO_3S$ $[M+H]^+$ 284.1320, found 284.1320

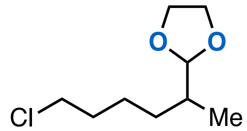

2-(6-chlorohexan-2-yl)-1,3-dioxolane (2l)
Prepared according to general procedure A using ethylene glycol. The title compound was isolated via flash chromatography (gradient 5 – 15% EtOAc/hexanes) as a colorless oil (57 mg, 59% yield).

TLC (SiO_2) R_f = 0.45 in 9:1 hexanes/EtOAc
^{1}H NMR (500 MHz, $CDCl_3$) (δ, ppm): 4.67 (d, J = 4.5 Hz, 1H), 4.17 – 3.90 (m, 2H), 3.94 – 3.79 (m, 2H), 3.53 (t, J = 6.7 Hz, 2H), 1.98 – 1.63 (m, 3H), 1.64 – 1.49 (m, 2H), 1.46 – 1.36 (m, 1H), 1.19 (m, 1H), 0.94 (d, J = 6.8 Hz, 3H).
^{13}C{H} NMR (126 MHz, $CDCl_3$) (δ, ppm): 107.6, 65.1, 65.1, 45.1, 36.9, 33.0, 30.8, 24.5, 13.9.
HRMS (ESI TOF): calcd. for $C_9H_{17}O_3NaCl$ $[M+Na]^+$ 231.0764, found 231.0765

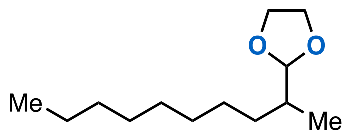

2-(decan-2-yl)-1,3-dioxolane (2m)
Prepared according to general procedure A using ethylene glycol. The title compound was isolated via flash chromatography (gradient 0 –5% EtOAc/hexanes) as a colorless oil (73 mg, 68% yield).

TLC (SiO_2) R_f = 0.16 in 98:2 hexanes/EtOAc
^{1}H NMR (400 MHz, $CDCl_3$) (δ, ppm): 4.67 (d, J = 4.4 Hz, 1H), 4.01 – 3.87 (m, 2H), 3.91 – 3.78 (m, 2H), 1.77 – 1.62 (m, 1H), 1.60 – 1.46 (m, 2H), 1.26 (m, 12H), 0.92 (d, J = 6.8 Hz, 3H), 0.91 – 0.67 (m, 3H).
^{13}C{H} NMR (101 MHz, $CDCl_3$) (δ, ppm): 107.9, 65.1, 37.0, 32.0, 31.7, 30.0, 29.7, 29.5, 27.2, 22.8, 14.2, 13.8.
HRMS(ESI TOF): calcd. for $C_{13}H_{27}O_2$ $[M+H]^+$ 215.2011, found 215.2012

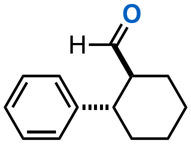

***trans*-2-phenylcyclohexane-1-carbaldehyde (2n)**
Prepared according to general procedure A on a 0.25 mmol scale. The title compound was isolated via flash chromatography (0 – 10% EtOAc/hexanes) as a colorless oil (30 mg, 65% yield). All analytical data for **2n** was in accordance with literature data.[12]

TLC (SiO_2) R_f = 0.35 in 9:1 hexanes/EtOAc
^{1}H NMR (500 MHz, $CDCl_3$) (δ, ppm): 9.41 (d, J = 3.0 Hz, 1H), 7.29 (dd, J = 8.3, 6.9 Hz, 2H), 7.22 – 7.16 (m, 3H), 2.74 (td, J = 11.7, 3.7 Hz, 1H), 2.60 (tt, J = 11.4, 3.4 Hz, 1H), 2.06 – 1.81 (m, 4H), 1.58 – 1.44 (m, 1H), 1.46 – 1.36 (m, 3H).
^{13}C{H} NMR (126 MHz, $CDCl_3$) δ 204.8, 144.2, 128.8, 127.5, 126.8, 55.5, 45.3, 35.0, 26.8, 26.3, 25.0.

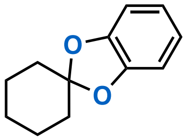

spiro[benzo[d][1,3]dioxole-2,1'-cyclohexane] (2o)
Prepared according to general procedure A using catechol. The title compound was isolated via flash chromatography (gradient 0–15% EtOAc/hexanes) as a colorless oil (47 mg, 43% yield). All analytical data for **2o** was in accordance with literature data.[13]

TLC (SiO_2) R_f = 0.29 in 9:1 hexanes/EtOAc
^{1}H NMR (400 MHz, $CDCl_3$) (δ, ppm): 7.29 – 7.24 (m, 4H), 1.62-1.45 (m, 4H), 1.35-1.13 (m, 6H)
^{13}C{H} NMR (101 MHz, $CDCl_3$) (δ, ppm): 147.3, 120.9, 118.2, 108.4, 35.2, 24.6, 23.2.

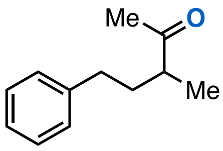

3-methyl-5-phenylpentan-2-one (2p)
Prepared according to general procedure A. The title compound was isolated via flash chromatography (gradient 5 –20% EtOAc/hexanes) as a colorless oil (48 mg, 54% yield). All analytical data for **2p** was in accordance with literature data.[14]

TLC (SiO_2) R_f = 0.18 in 9:1 hexanes/EtOAc
^{1}H NMR (500 MHz, $CDCl_3$) (δ, ppm): 7.40 – 7.34 (m, 3H), 7.31 – 7.20 (m, 2H), 2.68 (ddd, J = 9.3, 6.6, 2.9 Hz, 2H), 2.62 (q, J = 7.0 Hz, 1H), 2.21 (s, 3H), 2.10 (ddt, J = 13.8, 9.0, 6.9 Hz, 1H), 1.73 (ddt, J = 13.5, 9.1, 6.8 Hz, 1H), 1.22 (d, J = 7.0 Hz, 3H).
^{13}C{H} NMR (126 MHz, $CDCl_3$) (δ, ppm): 212.6, 141.8, 128.6, 128.5, 126.1, 46.6, 34.5, 33.5, 28.2, 16.4.

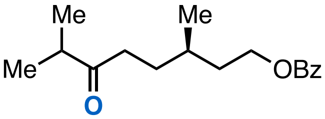

(R)-3,7-dimethyl-6-oxooctyl benzoate (2q)
Prepared according to general procedure A. The title compound was isolated via flash chromatography (gradient 5 –20% EtOAc/hexanes) as a colorless oil (55 mg, 40% yield).

TLC (SiO_2) R_f = 0.18 in 9:1 hexanes/EtOAc
^{1}H NMR (500 MHz, $CDCl_3$) (δ, ppm): 7.88 – 7.83 (m, 2H), 7.38 (dd, J = 8.3, 6.5 Hz, 1H), 7.26 (t, J = 7.7 Hz, 2H), 4.19 (tq, J = 11.0, 6.6, 5.6 Hz, 2H), 2.43 (hept, J = 7.0 Hz, 1H), 2.38 – 2.23 (m, 2H), 1.71 – 1.60 (m, 1H), 1.56 – 1.39 (m, 3H), 1.31 (ddd, J = 12.7, 9.1, 6.7 Hz, 1H), 0.91 (d, J = 7.0, 6H), 0.80 (d, J = 6.2 Hz, 3H).
^{13}C{H} NMR (126 MHz, $CDCl_3$) (δ, ppm): 214.9, 166.8, 133.0, 130.5, 129.7, 128.5, 63.4, 41.0, 38.0, 35.5, 30.8, 29.9, 19.4, 18.5, 18.4.
HRMS (ESI TOF): calcd. for $C_{17}H_{24}O_3Na$ $[M+Na]^+$ 299.1623, found 299.1626

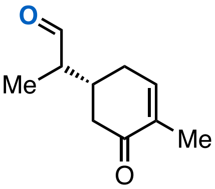

(R)-2-(4-methyl-5-oxocyclohex-3-en-1-yl)propanal (2r)
Prepared according to general procedure A. The title compound was isolated as a 1:1 mixture of diastereomers via flash chromatography (0 – 20% EtOAc/hexanes) as a colorless oil (51 mg, 61% yield). All analytical data for **2r** was in accordance with literature data.[15]

TLC (SiO_2) R_f = 0.19 in 85:15 hexanes/EtOAc

^{1}H NMR (400 MHz, $CDCl_3$) (δ, ppm): 9.66 (d, *J* = 1.8 Hz, 1H), 9.64 (d, *J* = 2.0 Hz, 1H), 6.72 (tdd, *J* = 5.8, 2.7, 1.4 Hz, 1H), 2.62 – 2.10 (m, 6H), 1.77 (dt, *J* = 2.7, 1.4 Hz, 3H), 1.13 (d, *J* = 2.4 Hz, 3H), 1.12 (d, *J* = 2.3 Hz, 3H).
^{13}C{H} NMR (101 MHz, $CDCl_3$) (δ, ppm): 203.5, 198.9, 198.9, 144.2, 144.2, 135.9, 135.9, 50.2, 50.2, 42.5, 41.0, 35.8, 35.7, 30.5, 28.7, 15.8, 15.8, 10.6, 10.2.

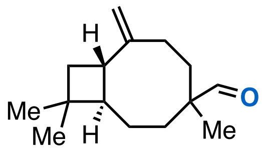

(1*R*,8*S*)-4,10,10-trimethyl-7-methylenebicyclo[6.2.0]decane-4-carbaldehyde (2s)
Prepared according to general procedure A. The title compound was isolated as a 4:1 mixture of stereoisomers via flash chromatography (9:1 hexanes/DCM) as a colorless oil (45.8 mg, 41% yield). All analytical data for **2s** was in accordance with literature data.[16]

TLC (SiO_2) R_f = 0.17 in 9:1 hexanes/DCM
^{1}H NMR (400 MHz, $CDCl_3$) (δ, ppm): 9.43 (s, 1H), 9.39 (s, 1H), 4.69 (t, *J* = 1.8 Hz, 1H), 4.49 (s, 1H), 2.65 (q, *J* = 9.4 Hz, 1H), 2.24 – 2.14 (m, 1H), 1.88 – 1.57 (m, 6H), 1.56 – 1.31 (m, 4H), 1.04 – 0.95 (m, 9H).
^{13}C{H} NMR (101 MHz, $CDCl_3$) (δ, ppm): 206.4, 153.2, 107.3, 52.4, 49.3, 40.2, 37.7, 34.4, 31.3, 30.2, 29.9, 29.8, 22.7, 22.5, 22.3.

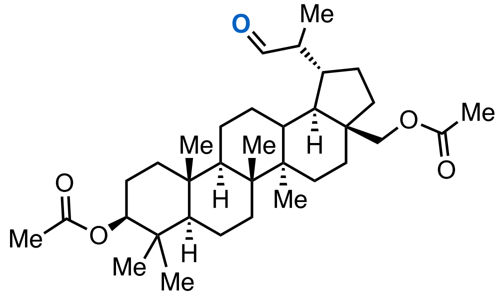

((1R,3aS,5aR,5bR,7aR,9S,11aR,11bR,13bR)-9-acetoxy-5a,5b,8,8,11a-pentamethyl-1-((S)-1-oxopropan-2-yl)icosahydro-3aH-cyclopenta[a]chrysen-3a-yl)methyl acetate (2t)
Prepared according to general procedure A on a 0.200 mmol scale. The title compound was isolated via preparative TLC (95:5 benzene/EtOAc) as a white solid (38 mg, 35% yield). All analytical data for **2t** was in accordance with literature data.[17]

TLC (SiO_2) R_f = 0.32 in 95:5 benzene/EtOAc
^{1}H NMR (500 MHz, $CDCl_3$) (δ, ppm): 9.85 (d, *J* = 1.9 Hz, 1H), 4.47 (dd, *J* = 11.0, 5.3 Hz, 1H), 4.21 (dd, *J* = 11.2, 1.8 Hz, 1H), 3.79 (dd, *J* = 11.1, 1.4 Hz, 1H), 2.64 – 2.54 (m, 1H), 2.06 (s, 3H), 2.04 (s, 3H), 1.96 – 1.15 (m, 23H), 1.10 (d, *J* = 7.0 Hz, 3H), 1.04 (s, 3H), 0.94 (s, 3H), 0.88 – 0.79 (m, 11H).
^{13}C{H} NMR (126 MHz, $CDCl_3$) (δ, ppm): 206.6, 171.7, 171.1, 81.0, 62.2, 55.4, 50.0, 49.3, 49.0, 46.5, 42.9, 42.8, 41.0, 38.5, 37.9, 37.3, 37.1, 34.4, 34.3, 29.8, 28.0, 27.7, 27.0, 24.8, 23.8, 21.4, 21.1, 20.9, 18.3, 16.6, 16.2, 16.1, 14.7, 14.6.

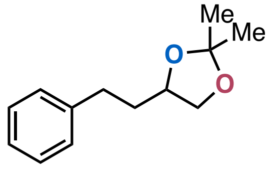

2,2-dimethyl-4-phenethyl-1,3-dioxolane (11a)

Prepared according to general procedure B. The title compound was isolated via flash chromatography (gradient 0 – 10% EtOAc/hexanes) as a colorless oil (62 mg, 59% yield). All analytical data for **3a** was in accordance with literature data.[18]

TLC (SiO_2) R_f = 0.46 in 9:1 hexane/EtOAc
^{1}H NMR (400 MHz, $CDCl_3$) (δ, ppm): 7.33 – 7.27 (m, 2H), 7.22 – 7.16 (m, 3H), 4.11 (tt, J = 7.1, 5.7 Hz, 1H), 4.01 (dd, J = 7.8, 5.9 Hz, 1H), 3.53 (t, J = 7.5 Hz, 1H), 2.77 (ddd, J = 13.8, 9.9, 5.6 Hz, 1H), 2.65 (ddd, J = 13.8, 9.7, 6.6 Hz, 1H), 1.96 (dddd, J = 13.0, 9.7, 7.1, 5.6 Hz, 1H), 1.82 (dddd, J = 13.5, 9.8, 6.6, 5.5 Hz, 1H), 1.44 (s,3H), 1.36 (s, 3H).
^{13}C{H} NMR (101 MHz, $CDCl_3$) (δ, ppm): 141.7, 128.5, 128.5, 126.1, 108.9, 75.5, 69.5, 35.5, 32.1, 27.1, 25.9.

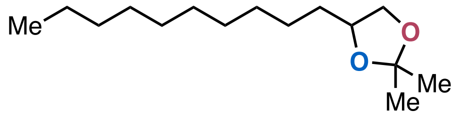

4-decyl-2,2-dimethyl-1,3-dioxolane (11b)

Prepared according to general procedure B. The title compound was isolated via flash chromatography (gradient 0 – 10% EtOAc/hexanes) as a colorless oil (64 mg, 52% yield). All analytical data for **3b** was in accordance with literature data.[19]

TLC (SiO_2) R_f = 0.66 in 9:1 hexanes/EtOAc
^{1}H NMR (500 MHz, CDCl3) (δ, ppm): 4.11 – 4.00 (m, 2H), 3.50 (t, J = 7.4 Hz, 1H), 1.69 – 1.59 (m, 1H), 1.51 – 1.44 (m, 1H), 1.41 (s, 3H), 1.35 (s, 3H), 1.30 – 1.24 (m, 16H), 0.88 (t, J = 6.8 Hz, 3H).
^{13}C{H} NMR (126 MHz, $CDCl_3$) δ 108.7, 76.3, 69.7, 33.8, 32.0, 29.8, 29.7, 29.7, 29.6, 29.45, 27.1, 25.9, 22.8, 14.2.

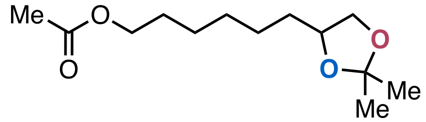

6-(2,2-dimethyl-1,3-dioxolan-4-yl)hexyl acetate (11c)

Prepared according to general procedure B. The title compound was isolated via flash chromatography (gradient 0 –20% EtOAc/hexanes) as a colorless oil (53 mg, 43% yield).

TLC (SiO_2) R_f = 0.30 in 9:1 hexanes/EtOAc
^{1}H NMR (500 MHz, $CDCl_3$) (δ, ppm): 4.05 (m, 4H), 3.49 (t, J = 7.3 Hz, 1H), 2.04 (s, 3H), 1.68 – 1.54 (m, 4H), 1.53 – 1.42 (m, 1H), 1.40 (s, 3H), 1.38 – 1.32 (m, 8H).
^{13}C{H} NMR (126 MHz, $CDCl_3$) (δ, ppm): 171.4, 108.8, 76.2, 69.6, 64.7, 33.6, 29.4, 28.6, 27.1, 25.9, 25.9, 25.8, 21.1.
HRMS (ESI TOF): calcd. for $C_{13}H_{24}O_4Na$ $[M+Na]^+$ 267.1572, found 267.1574

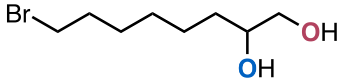

4-(6-bromohexyl)-2,2-dimethyl-1,3-dioxolane (11d)

Prepared according to a modified general procedure B. The acetonide was isolated via flash chromatography (gradient 0 –5% EtOAc/hexanes) to afford the crude acetonide. The crude acetonide was dissolved in 5 mL of IPA and 0.5 mL of 1M HCl (aq) was added. The mixture was heated to 65 ºC for 4 hours. Upon cooling to room temperature, the mixture was transferred to a separatory funnel and diluted with water (15 mL) and EtOAc (15 mL). The layers were separated and the organic layer was washed 2 times with 10 mL of water. The organic layer was dried over sodium sulfate and the solvent was removed to afford the crude product, which was isolated via flash chromatography (1:1 hexane/EtOAc) to afford the diol as a colorless oil (42 mg, 37% yield). All analytical data for **3b** was in accordance with literature data.[20]

TLC (SiO_2) R_f = 0.15 in 1:1 hexane/EtOAc
^{1}H NMR (500 MHz, $CDCl_3$) (δ, ppm): 3.70 (dtd, *J* = 9.8, 6.6, 6.1, 2.9 Hz, 1H), 3.65 (dd, *J* = 11.0, 3.0 Hz, 1H), 3.48 – 3.37 (m, 3H), 2.23 (s, 2H), 1.85 (p, *J* = 6.9 Hz, 2H), 1.50 – 1.40 (m, 5H), 1.38 – 1.25 (m, 3H).
^{13}C{H} NMR (126 MHz, $CDCl_3$) (δ, ppm): 72.3, 66.9, 34.0, 33.1, 32.8, 28.9, 28.2, 25.5.

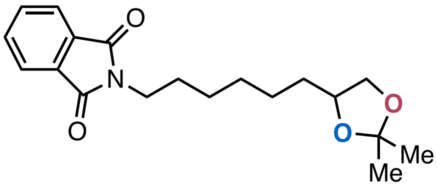

2-(6-(2,2-dimethyl-1,3-dioxolan-4-yl)hexyl)isoindoline-1,3-dione (11e)

Prepared according to general procedure B. The title compound was isolated via flash chromatography (gradient 10 – 30% EtOAc/hexanes) as a light yellow solid (58 mg, 35% yield).

TLC (SiO_2) R_f = 0.13 in 85:15 hexane/EtOAc
^{1}H NMR (500 MHz, $CDCl_3$) (δ, ppm): 7.84 (dd, *J* = 5.4, 3.1 Hz, 2H), 7.71 (dd, *J* = 5.4, 3.0 Hz, 2H), 4.09 – 3.98 (m, 2H), 3.67 (t, *J* = 7.3 Hz, 2H), 3.48 (t, *J* = 7.3 Hz, 1H), 1.72 – 1.57 (m, 3H), 1.52 – 1.41 (m, 1H), 1.45 – 1.26 (m, 11H).
^{13}C{H} NMR (126 MHz, $CDCl_3$) (δ, ppm) 168.6, 134.0, 133.9, 132.3, 123.3, 108.7, 76.2, 69.6, 38.1, 33.6, 29.3, 28.6, 27.1, 26.9, 25.9.
HRMS (ESI TOF): calcd. for $C_{19}H_{25}NO_4Na$ $[M+Na]^+$ 354.1681, found 354.1679

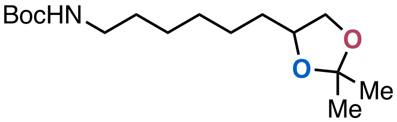

***tert*-butyl (6-(2,2-dimethyl-1,3-dioxolan-4-yl)hexyl)carbamate (11f)**

Prepared according to general procedure B. The title compound was isolated via flash chromatography (gradient 0 – 30% EtOAc/hexanes), followed by preparative TLC (9:1 hexane/EtOAc) as a colorless oil (63 mg, 42% yield).

TLC (SiO_2) R_f = 0.13 in 95:5 hexanes/EtOAc
^{1}H NMR (500 MHz, $CDCl_3$) (δ, ppm): 4.48 (s, 1H), 4.11 – 3.99 (m, 2H), 3.49 (t, *J* = 7.3 Hz, 1H), 3.10 (q, *J* = 6.8 Hz, 1H), 1.71 – 1.54 (m, 2H), 1.44 (s, 12H), 1.40 (s, 3H), 1.36 – 1.24 (m ,9H).
^{13}C{H} NMR (126 MHz, $CDCl_3$) (δ, ppm): 156.1, 108.8, 76.2, 69.7, 40.7, 33.7, 30.1, 29.4, 28.6, 28.5, 27.1, 26.8, 25.9, 25.9.
HRMS (ESI TOF): calcd. for $C_{16}H_{31}NO_4Na$ $[M+Na]^+$ 324.2151, found 324.2147

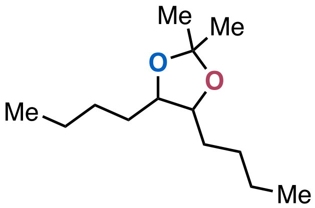

4,5-dibutyl-2,2-dimethyl-1,3-dioxolane (11g)
Prepared according to general procedure B. The title compound was isolated as a 3:1 mixture of stereoisomers via flash chromatography (gradient 0 – 40% DCM/hexanes) as a colorless oil (51 mg, 48% yield). All analytical data for **3g** was in accordance with literature data.[21]

TLC (SiO_2) R_f = 0.24 in 8:2 DCM/hexanes
^{1}H NMR (400 MHz, $CDCl_3$) (δ, ppm): 4.05 – 3.97 (m, 2H), 3.64 – 3.53 (m, 2H), 1.56 – 1.41 (m, 6H), 1.37 (s, 6H), 1.36 – 1.25 (m, 6H), 0.94 – 0.87 (m, 6H).
^{13}C{H} NMR (101 MHz, $CDCl_3$) (δ, ppm): 107.8, 107.3, 81.1, 78.2, 32.8, 29.5, 28.8, 28.5, 28.4, 27.4, 26.2, 23.0, 22.9, 14.1, 14.1.

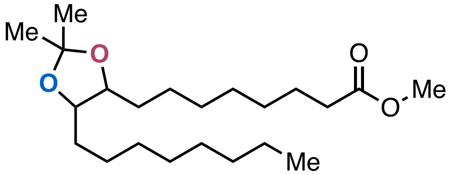

methyl 8-(2,2-dimethyl-5-octyl-1,3-dioxolan-4-yl)octanoate (11h)
Prepared according to general procedure B. The title compound was isolated via flash chromatography (gradient 0 – 10% EtOAc/hexanes) as a 9:1 mixture of diastereomers as a colorless oil (114 mg, 62% yield). 9:1 dr was determined by GCMS analysis.

TLC (SiO_2) R_f = 0.15 in 95:5 hexane/EtOAc
^{1}H NMR (400 MHz, $CDCl_3$) (δ, ppm): 3.66 (s, 3H), 3.63 – 3.50 (m, 2H), 2.30 (t, *J* = 7.5 Hz, 2H), 1.62 (q, *J* = 7.2 Hz, 2H), 1.49 (m, 6H), 1.37 (s, 6H), 1.35 – 1.10 (m, 18H), 0.92 – 0.85 (m, 3H).
^{13}C{H} NMR (101 MHz, $CDCl_3$) (δ, ppm): 174.4, 107.9, 81.2, 81.1, 51.6, 34.2, 33.2, 33.1, 32.0, 29.9, 29.7, 29.6, 29.4, 29.3, 29.2, 27.5, 26.3, 26.3, 25.0, 22.8, 14.2.
HRMS (ESI TOF): calcd. for $C_{22}H_{42}O_4Na$ $[M+Na]^+$ 393.2981, found 393.2976

Mechanism studies

1,2-Shift studies

Detection of 1,2-shift of deuterated alkene. The reaction was carried out following general procedure A on a 0.2 mmol scale using ethylene glycol. The product was isolated using flash chromatography (0 – 5% EtOAc in hexanes) to afford the product as a colorless oil (23 mg, 55% yield). The deuterium incorporation was determined by ^{1}H NMR analysis of the protected aldehyde product.

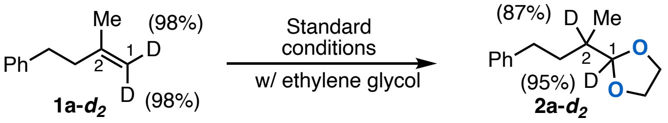

^{1}H NMR (500 MHz, CDCl$_3$) of 2-(4-phenylbutan-2-yl-2-*d*)-1,3-dioxolane-2-*d*

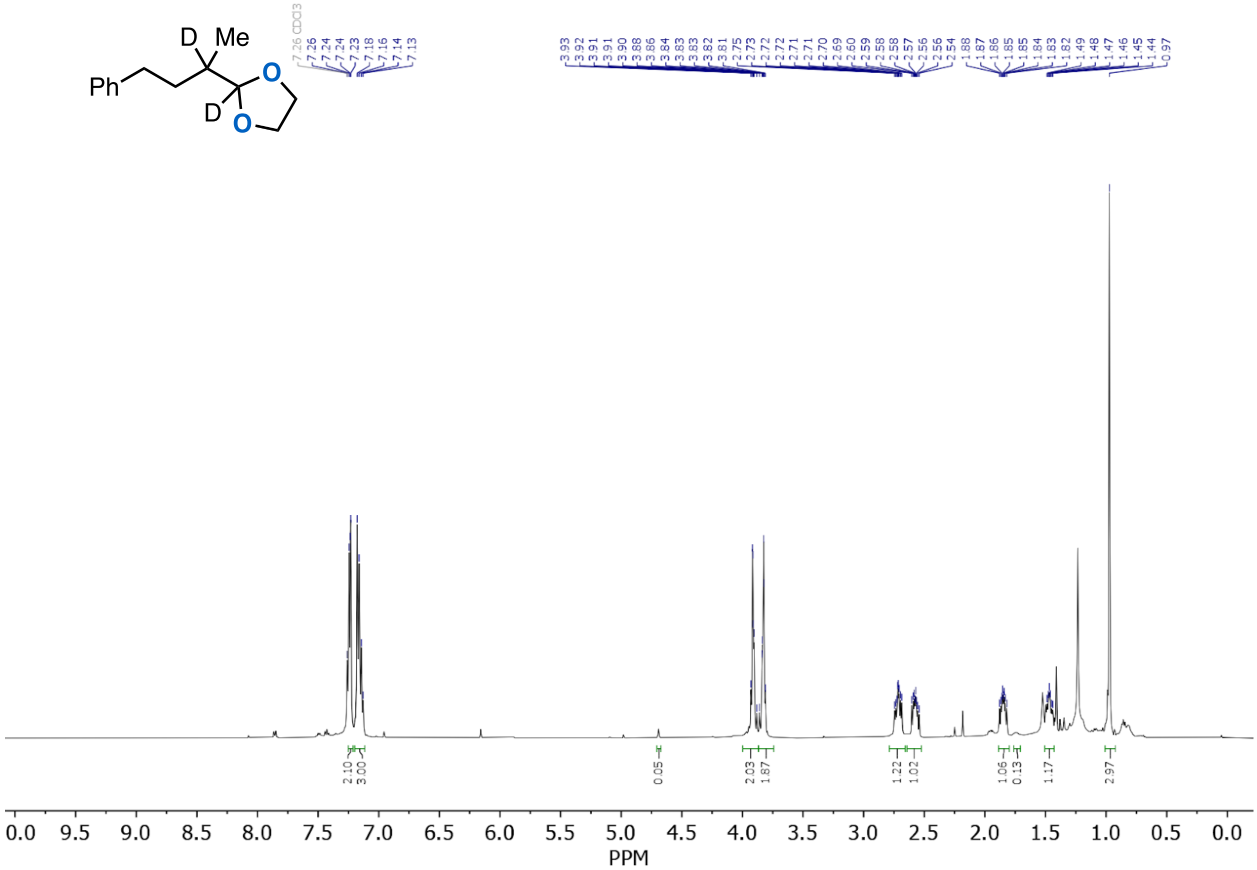

Comparison to Meinwald Rearrangement

Meinwald ring opening of trisubstituted epoxide. To a 4-dram oven-dried vial equipped with a stir bar was added 2,3-dimethyl-2-phenethyloxirane (88.1 mg, 1 equiv., 0.50 mmol) and DCE (5 mL). The mixture was cooled to 0 ºC and then $Fe(OTf)_2$ (17.7 mg, 0.05 mmol, 10 mol%) was added. The mixture was stirred for 2 hours, and the outcome of the reaction was analyzed by GCMS.

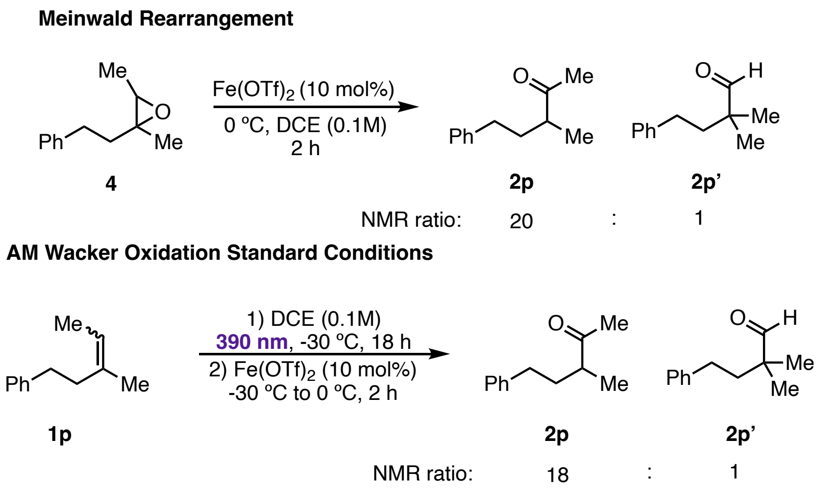

Figure S3: Meinwald rearrangement of epoxide **4** and AM Wacker oxidation of alkene **1p** to determine the ratio of hydride to alkyl shift. NMR ratio determined by ^{1}H NMR using CH_2Br_2 as an external standard.

Detection of potential reaction intermediates

We investigated the potential of going through a transient epoxide intermediate as a pathway for the formation of the AM Wacker oxidation products. We subjected diadamantyl alkene **5** to the standard reaction conditions on a 0.50 mmol scale and monitored for the formation of epoxide **6**, which is less prone to rearrangements under Lewis acid catalysis.[22] At the end of the reaction, epoxide **6** was not detected in the reaction mixture and only the product of oxidative cleavage was present at the end of the reaction. This suggests that it is unlikely that the mechanism proceeds through an epoxide intermediate and additionally, that Lewis acid coordination to the dioxazolidine intermediate is potentially reversible.

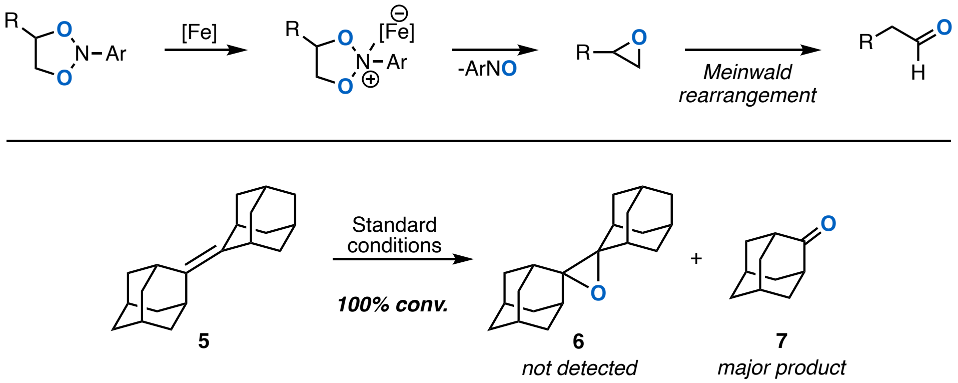

Figure S4: Potential mechanistic pathway with an epoxide intermediate. Reaction of alkene **5** under standard conditions for detection of epoxide intermediate **6**.

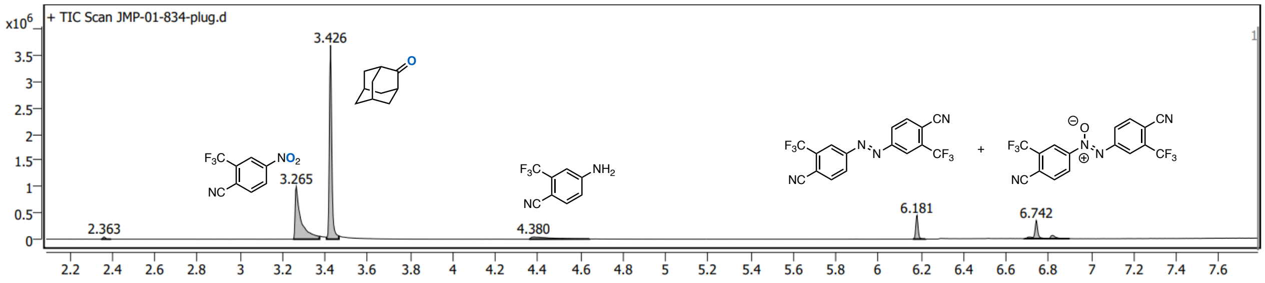

We examined how the addition of radical trapping agents and radical quenching agents would impact the reaction to investigate a potential SET-promoted radical fragmentation pathway to generate the AM Wacker oxidation products. Galvinoxyl radical did not significantly affect the reaction, and no TEMPO trapping products were detected, indicating that it is unlikely that radical intermediates are generated from the fragmentation of the dioxazolidine Lewis acid complex.

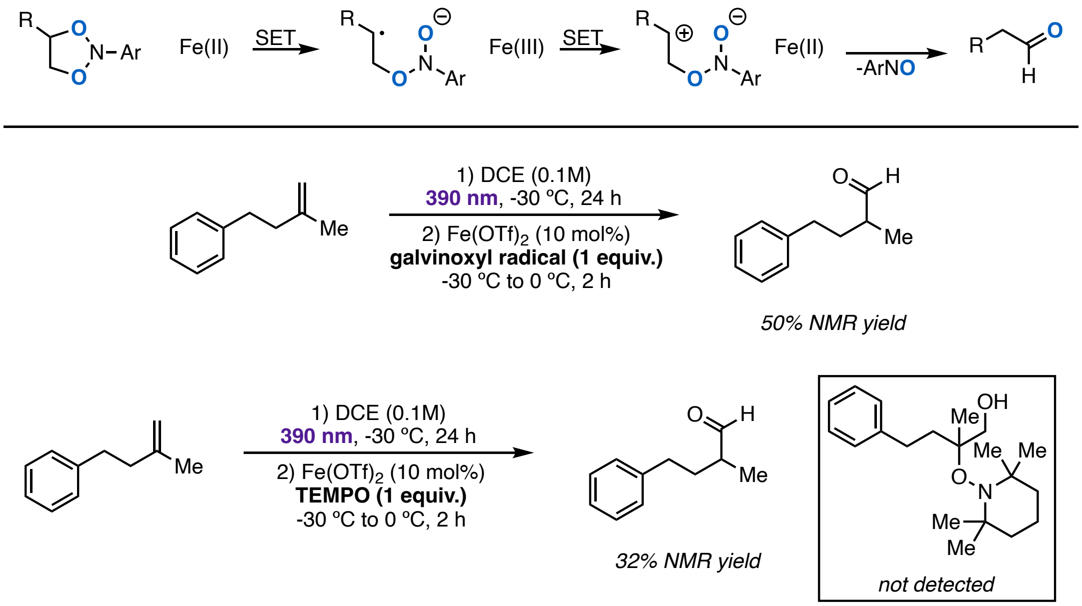

Figure S5: Potential radical fragmentation pathway via Fe(II)/Fe(III) catalytic cycle. Radical quenching and trapping studies with galvinoxyl radical and TEMPO.

Isolation of S_N2 product

Alkene **12** was subjected to standard reaction conditions using general procedure B. At the end of the reaction, the expected acetonide product was not detected, but instead compound **13** was isolated in a 29% yield. The addition of acetone to the less substituted carbon is indicative of an S_N2-type mechanism where acetone attacks preferentially at the more sterically accessible site.

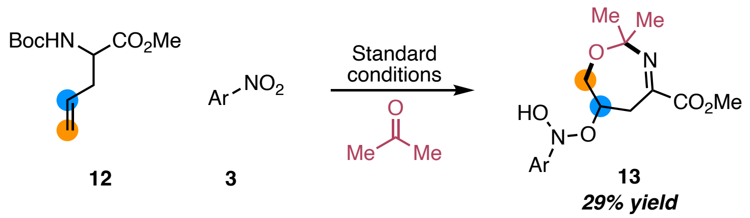

<u>Characterization of compound **13**</u>

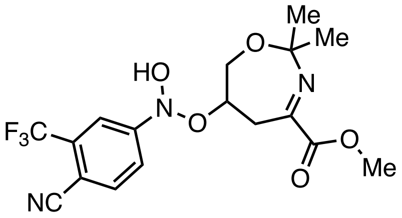

Methyl 6-(((4-cyano-3-(trifluoromethyl)phenyl)(hydroxy)amino)oxy)-2,2-dimethyl-2,5,6,7-tetrahydro-1,3-oxazepine-4-carboxylate (13)

^{1}H NMR (400 MHz, $CDCl_3$) (δ, ppm): 12.40 (s, 1H), 7.71 (d, J = 8.5 Hz, 1H), 7.52 (d, J = 2.2 Hz, 1H), 7.30 (dd, J = 8.5, 2.2 Hz, 1H), 4.49 (p, J = 6.2 Hz, 1H), 4.14 (dd, J = 8.3, 6.0 Hz, 1H), 3.87 (s, 3H), 3.77 – 3.69 (m, 1H), 2.95 (dd, J = 15.4, 6.3 Hz, 1H), 2.75 (dd, J = 15.4, 6.7 Hz, 1H), 1.44 (s, 3H), 1.37 (s, 3H).
^{13}C{H} NMR (101 MHz, $CDCl_3$) (δ, ppm): 172.9, 163.6, 146.8, 136.3, 134.8 (q, J = 32.9 Hz), 130.6, 122.4 (q, J = 274.0 Hz), 116.3, 115.9, 111.7, 111.7, 111.6, 109.3, 101.3, 101.3, 73.8, 69.2, 52.5, 37.5, 28.4, 27.1, 25.6.
^{19}F NMR (377 MHz, $CDCl_3$) (δ, ppm): -62.41.
HRMS (ESI TOF): calcd. for $C_{17}H_{19}N_3O_5F_3$ $[M+H]^+$ 402.1277, found 402.1271

^{1}H NMR (400 MHz, CDCl₃) of Methyl 6-(((4-cyano-3-(trifluoromethyl)phenyl)(hydroxy)amino)oxy)-2,2-dimethyl-2,5,6,7-tetrahydro-1,3-oxazepine-4-carboxylate (13)

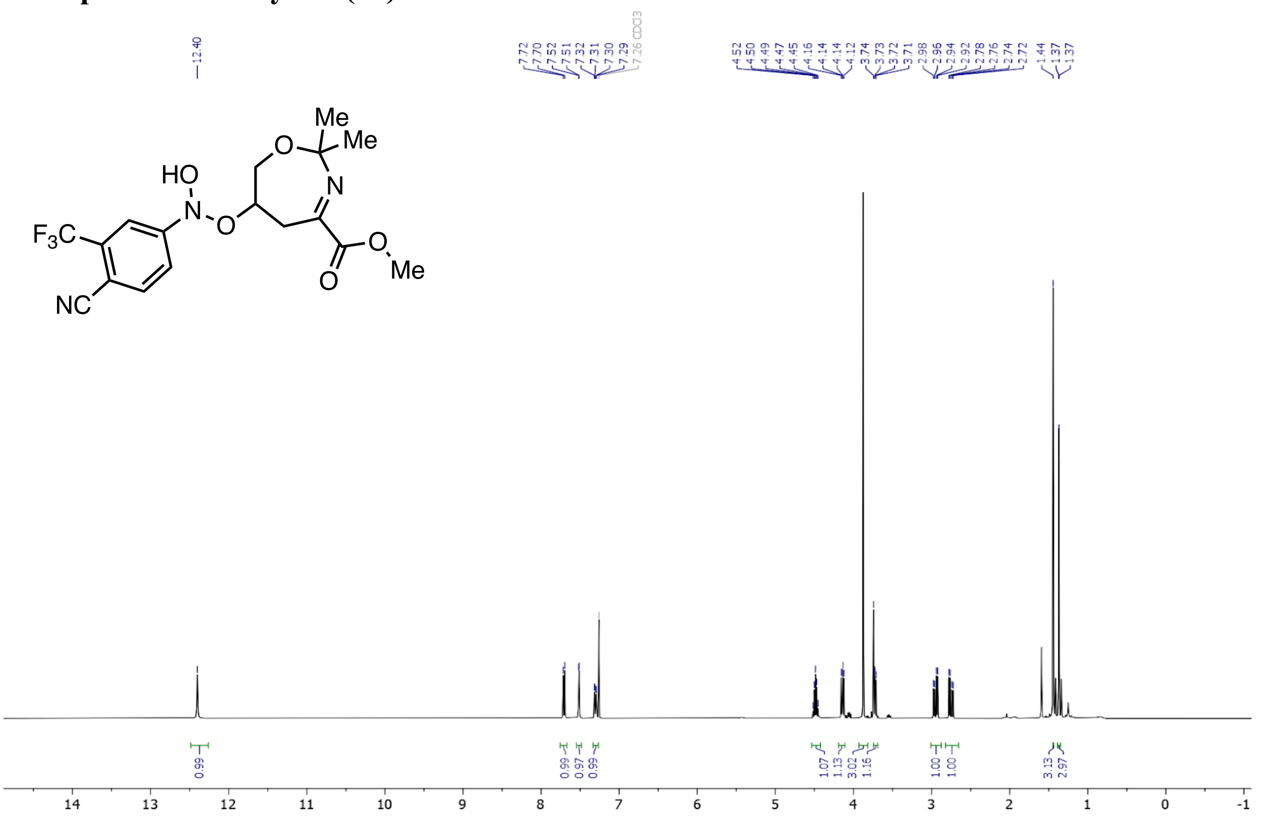

$^{13}C\{H\}$ NMR (101 MHz, $CDCl_3$) of Methyl 6-(((4-cyano-3-(trifluoromethyl)phenyl)(hydroxy)amino)oxy)-2,2-dimethyl-2,5,6,7-tetrahydro-1,3-oxazepine-4-carboxylate (13)

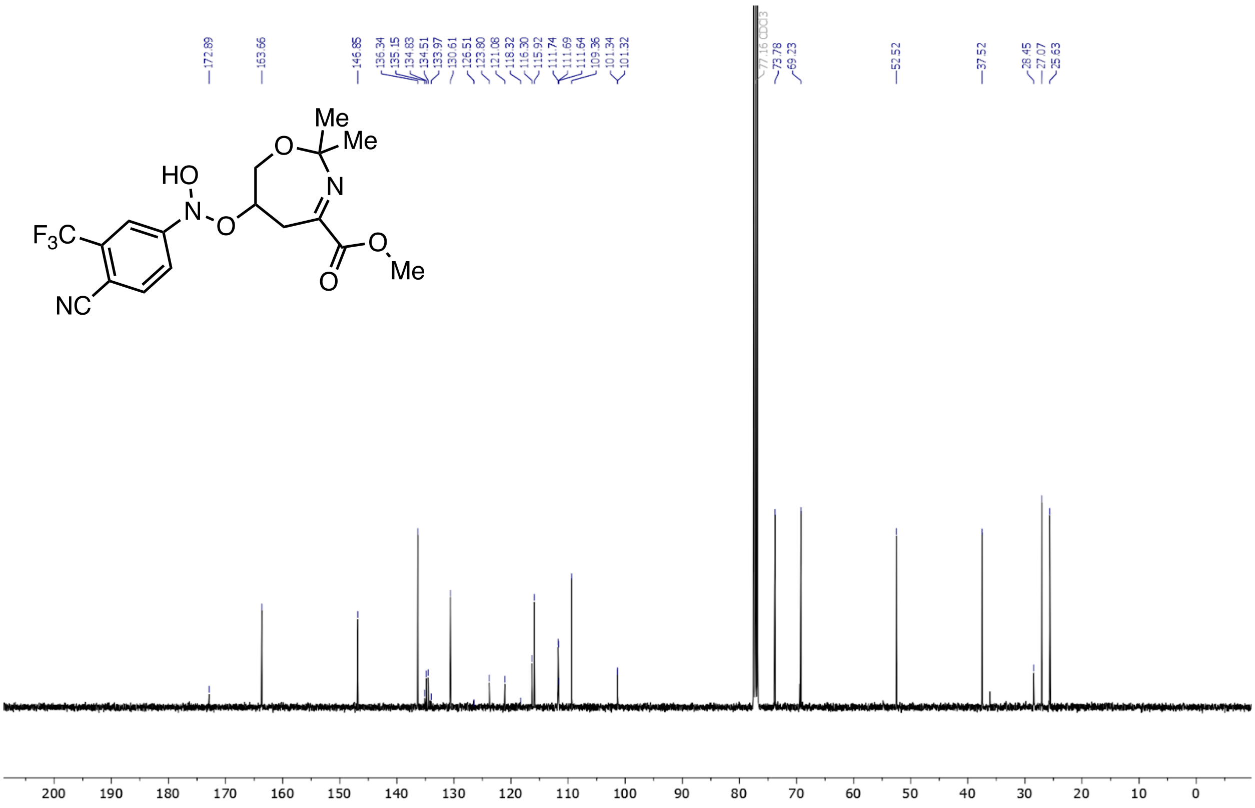

^{19}F NMR (377 MHz, $CDCl_3$) of Methyl 6-(((4-cyano-3-(trifluoromethyl)phenyl)(hydroxy)amino)oxy)-2,2-dimethyl-2,5,6,7-tetrahydro-1,3-oxazepine-4-carboxylate (13)

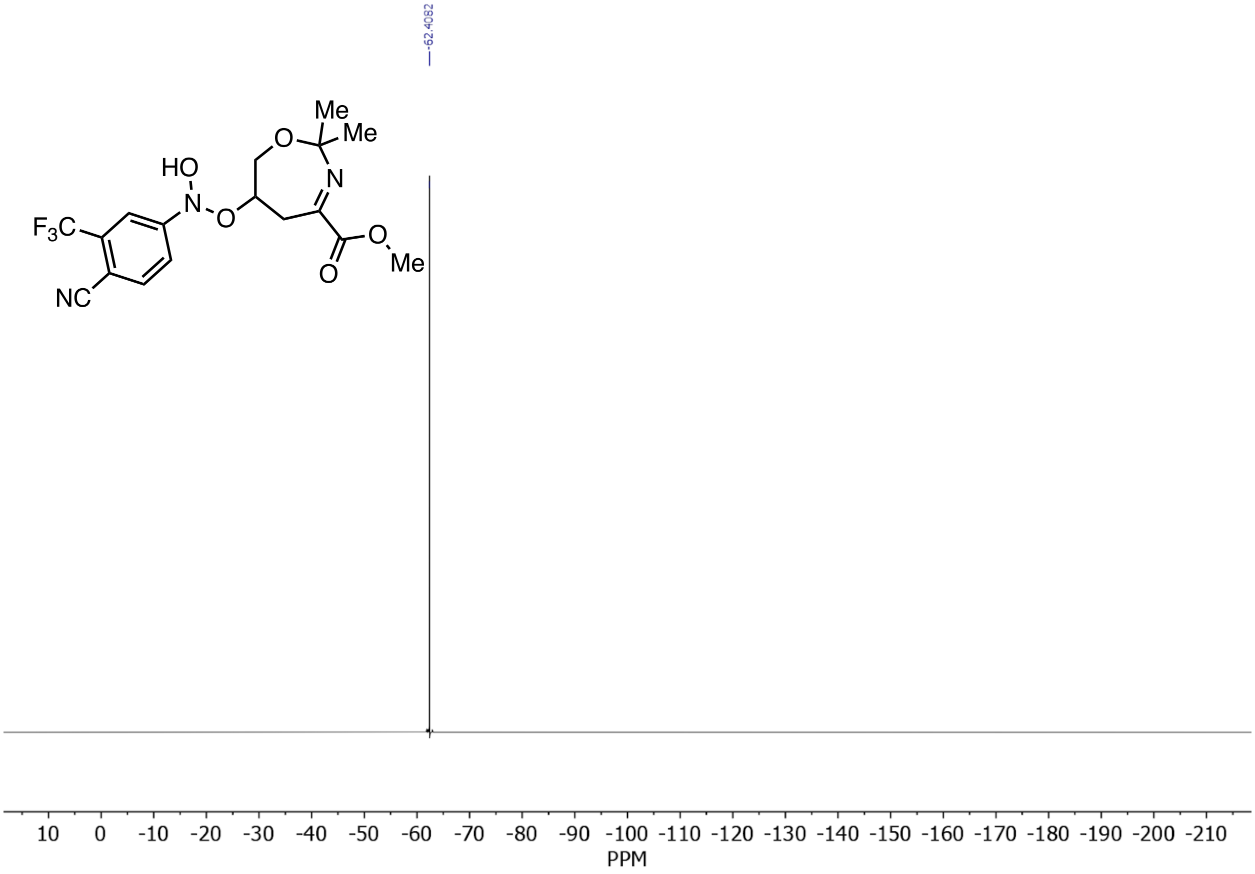

^{1}H–^{13}C DEPT-135 (400 MHz, $CDCl_3$) of Methyl 6-(((4-cyano-3-(trifluoromethyl)phenyl)(hydroxy)amino)oxy)-2,2-dimethyl-2,5,6,7-tetrahydro-1,3-oxazepine-4-carboxylate (13)

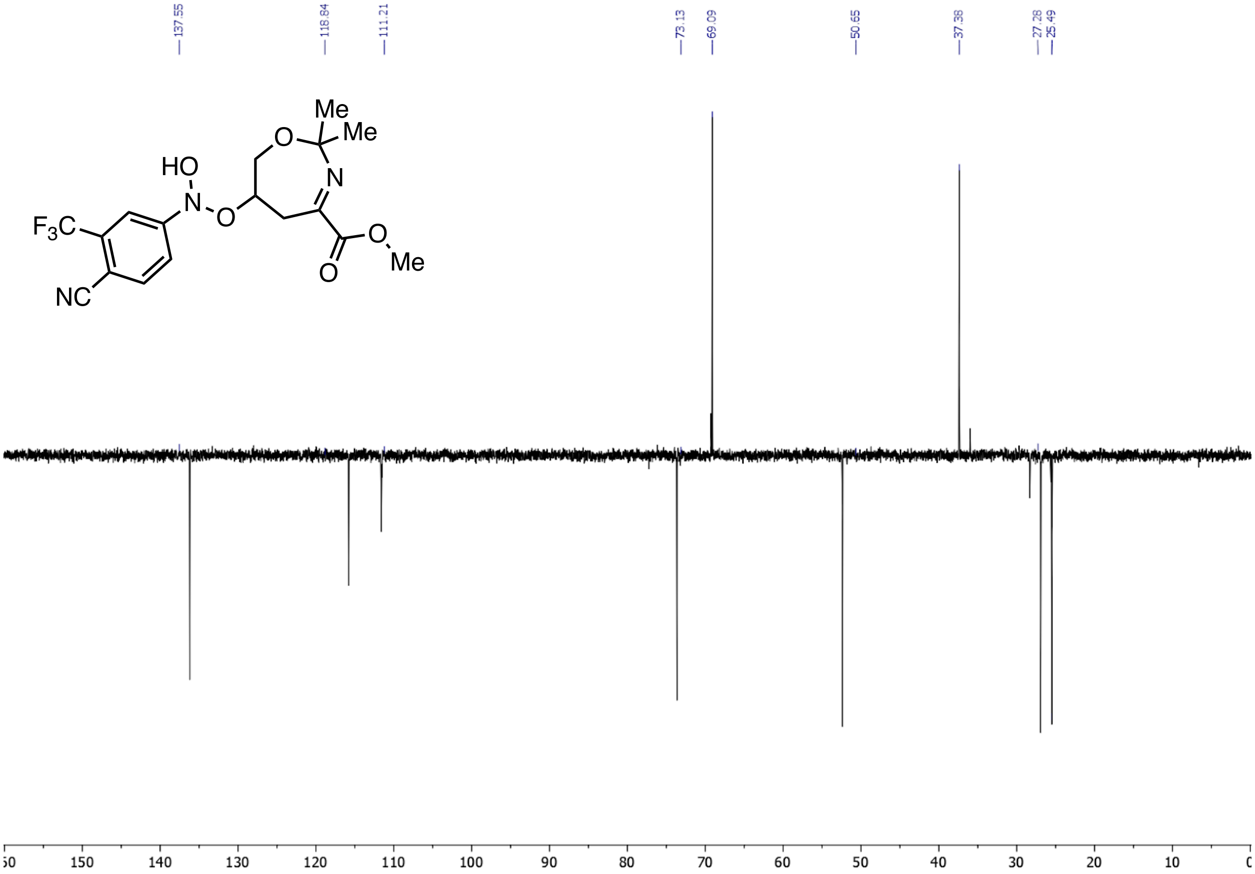

^{1}H–^{1}H COSY (400 MHz, $CDCl_3$) of Methyl 6-(((4-cyano-3-(trifluoromethyl)phenyl)(hydroxy)amino)oxy)-2,2-dimethyl-2,5,6,7-tetrahydro-1,3-oxazepine-4-carboxylate (13)

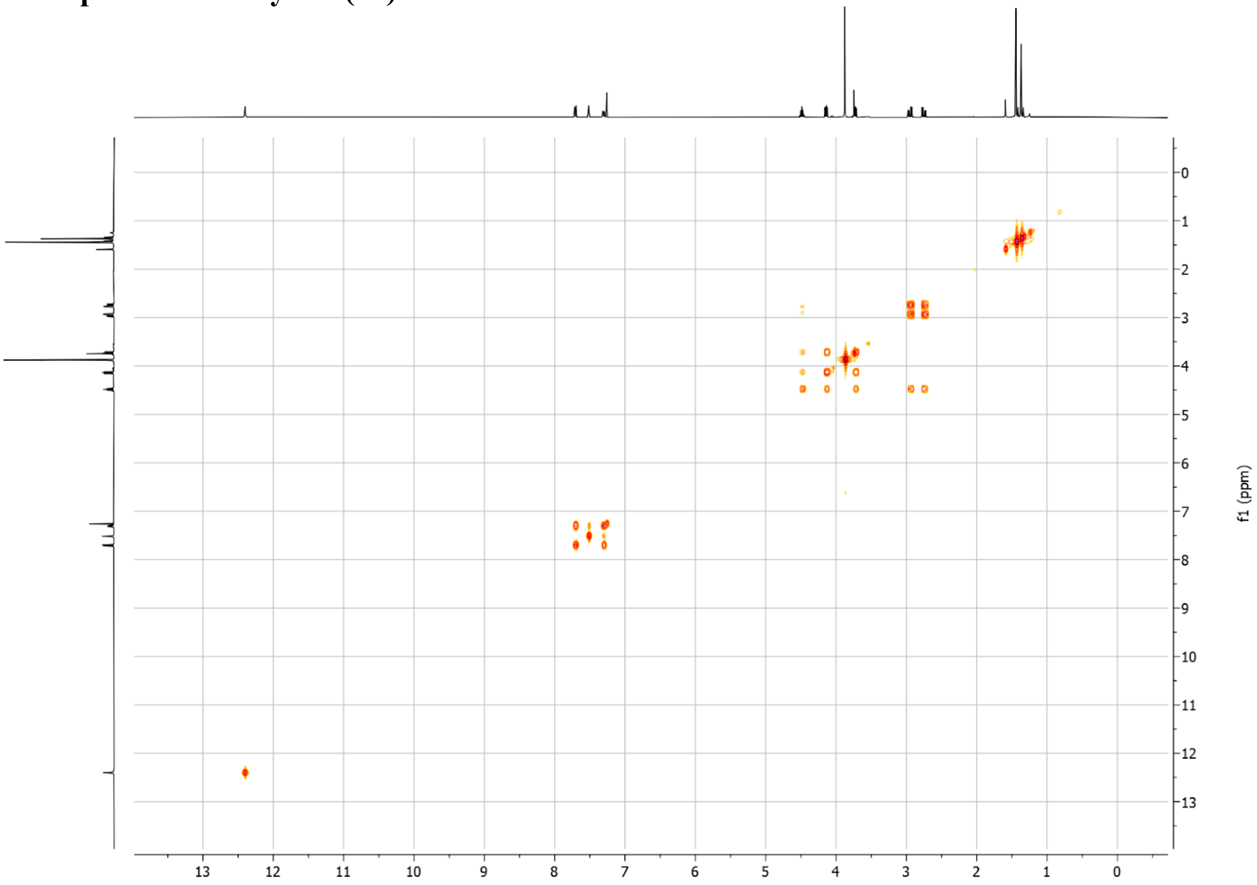

^{1}H–^{13}C HSQC (400 MHz, $CDCl_3$) of Methyl 6-(((4-cyano-3-(trifluoromethyl)phenyl)(hydroxy)amino)oxy)-2,2-dimethyl-2,5,6,7-tetrahydro-1,3-oxazepine-4-carboxylate (13)

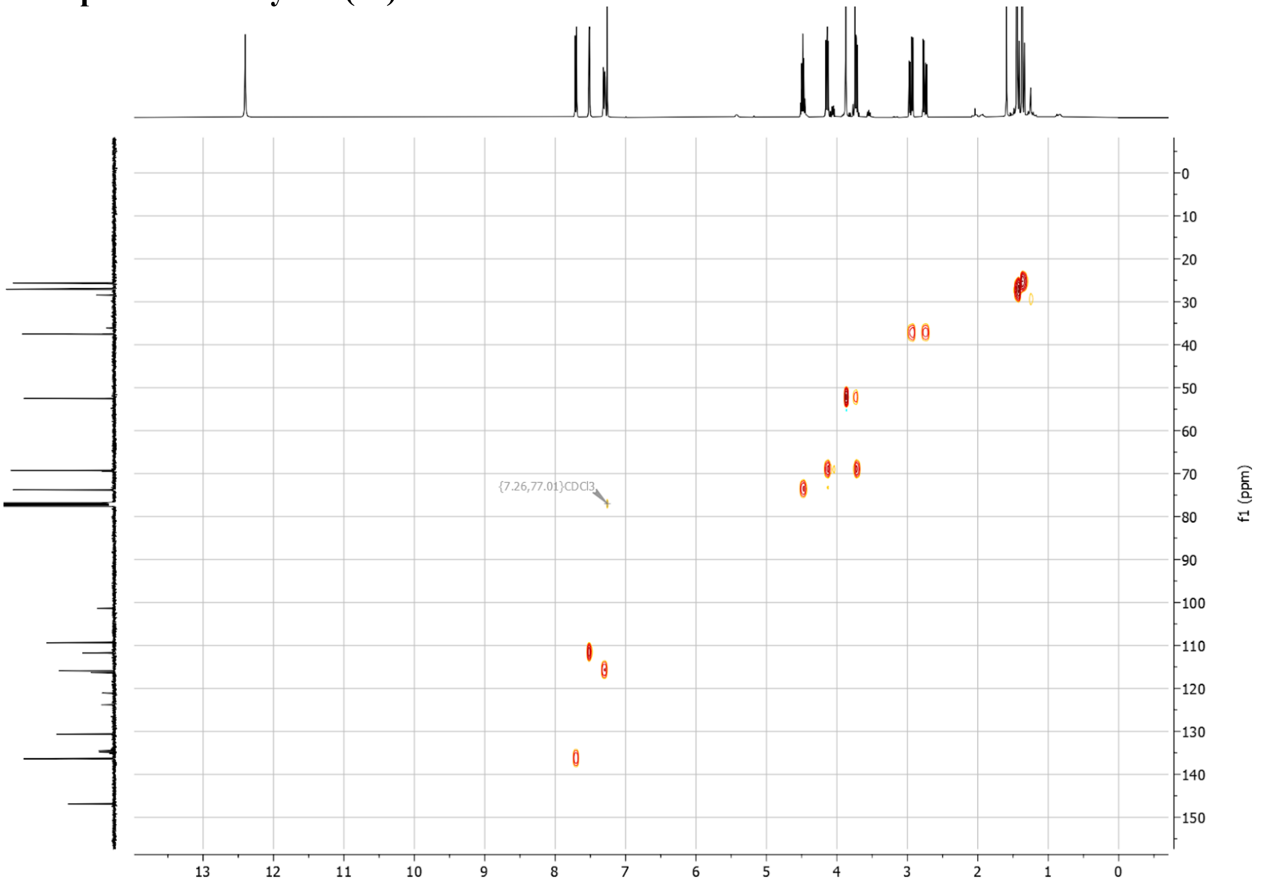

^{1}H–^{13}C HMBC (400 MHz, $CDCl_3$) of Methyl 6-(((4-cyano-3-(trifluoromethyl)phenyl)(hydroxy)amino)oxy)-2,2-dimethyl-2,5,6,7-tetrahydro-1,3-oxazepine-4-carboxylate (13)

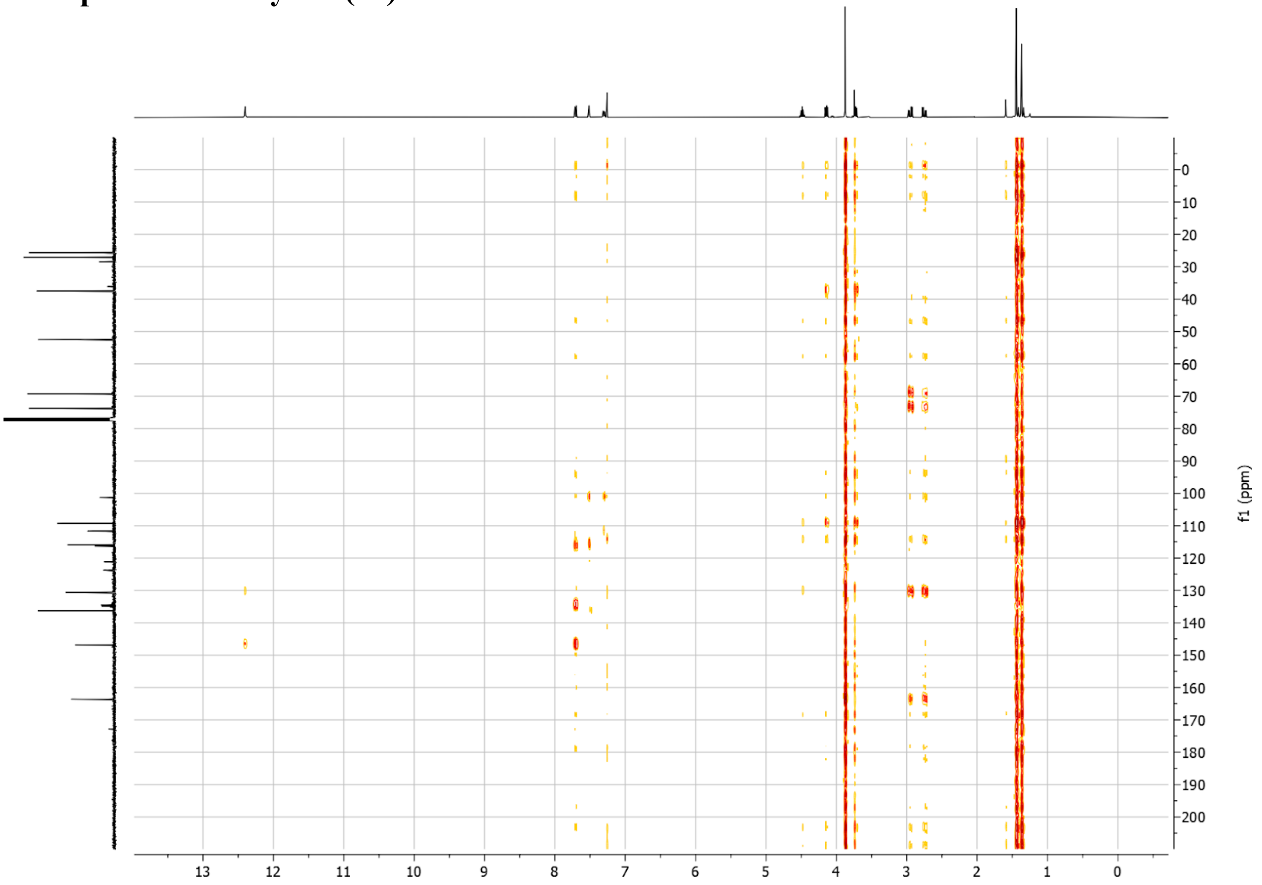

NMR spectra

^{1}H NMR (500 MHz, $CDCl_3$) of 4-(3-methylbut-3-en-1-yl)phenyl benzoate (1g)

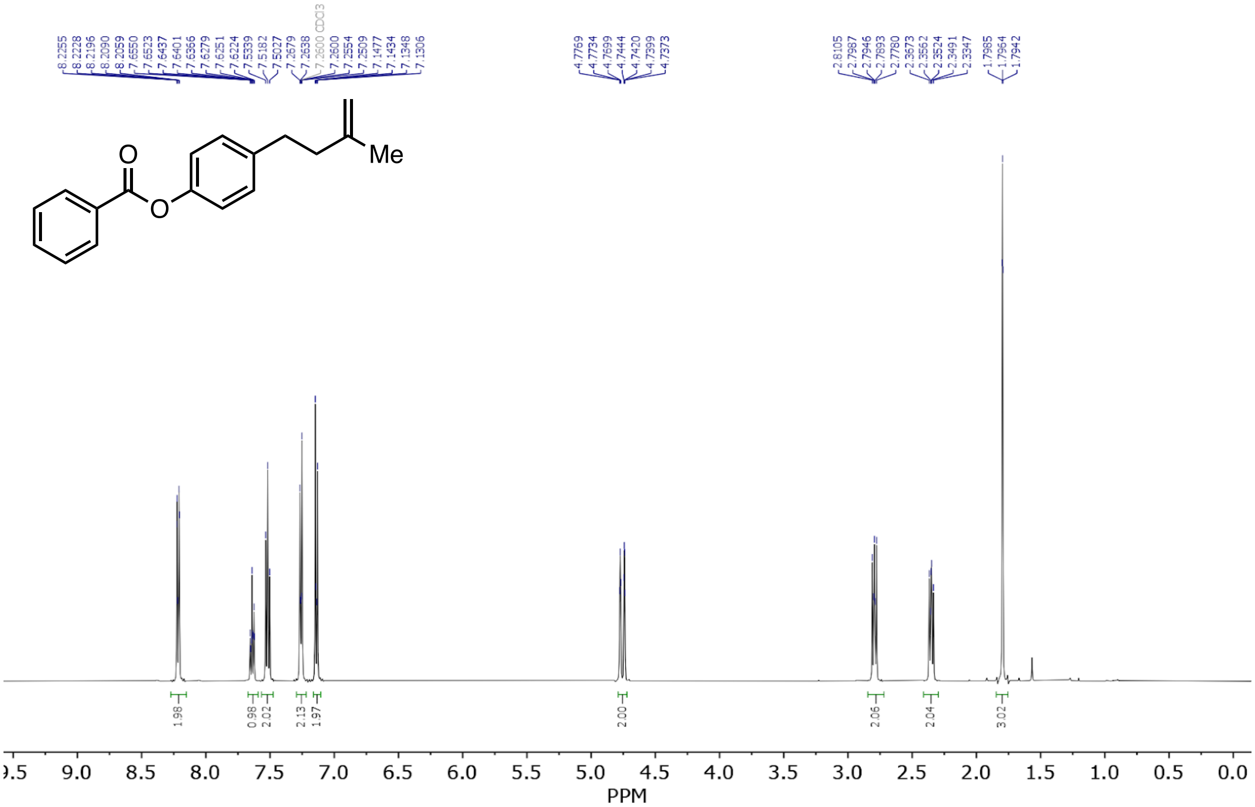

^{13}C{H} NMR (126 MHz, $CDCl_3$) of 4-(3-methylbut-3-en-1-yl)phenyl benzoate (1g)

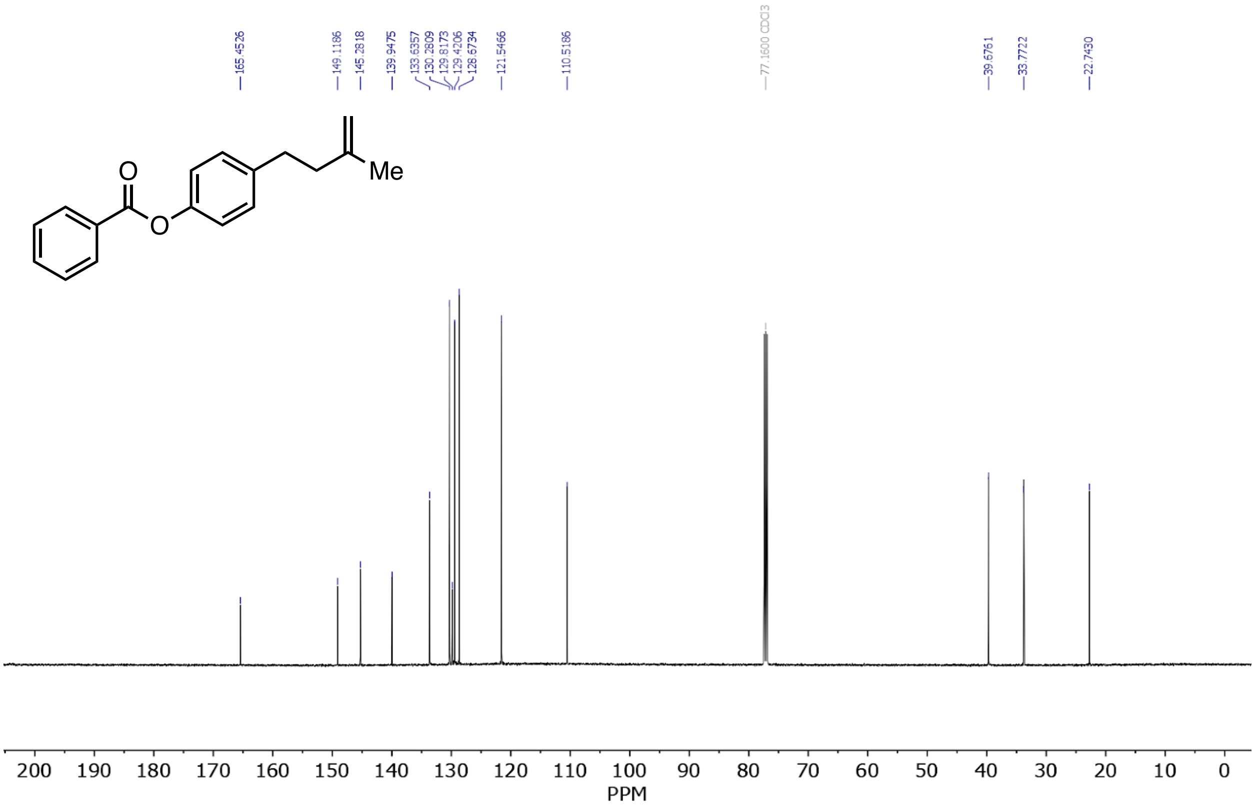

¹H NMR (400 MHz, $CDCl_3$) of 5-methylhex-5-en-1-yl acetate (1h)

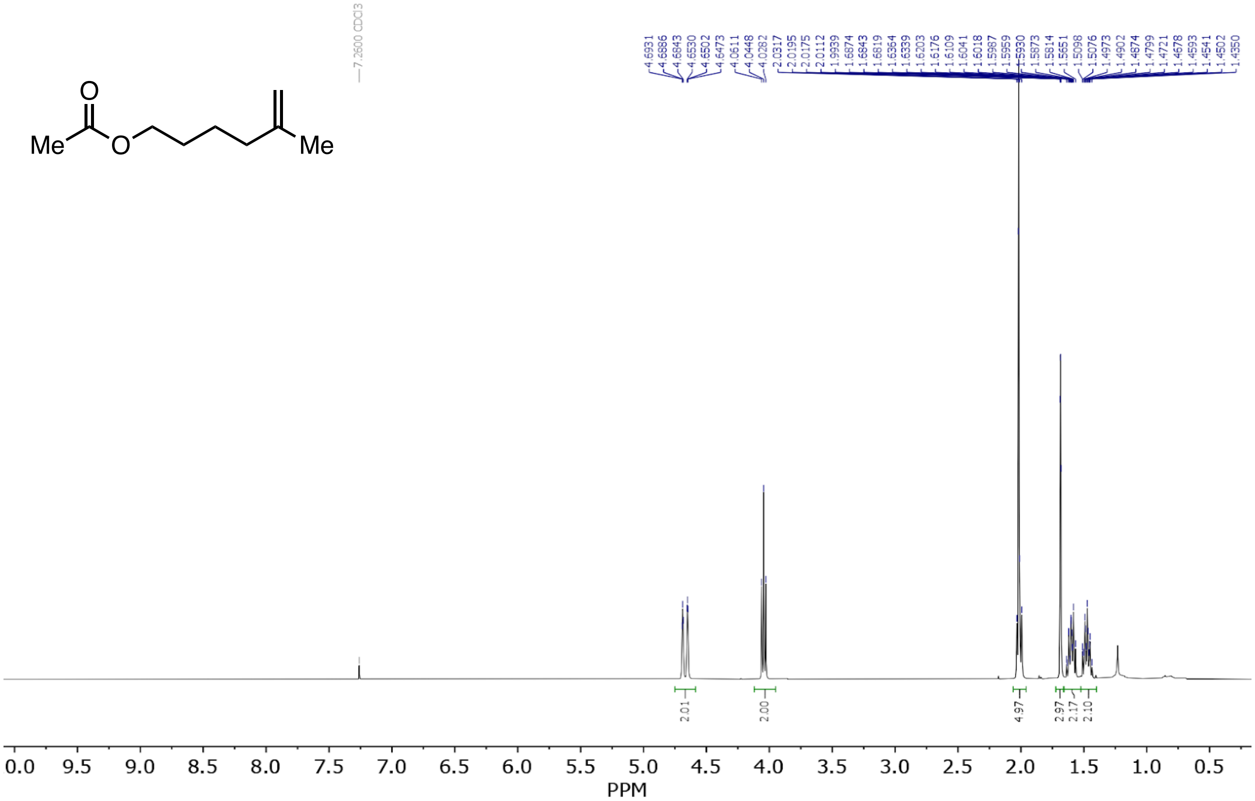

¹³C{H} NMR (101 MHz, $CDCl_3$) of 5-methylhex-5-en-1-yl acetate (1h)

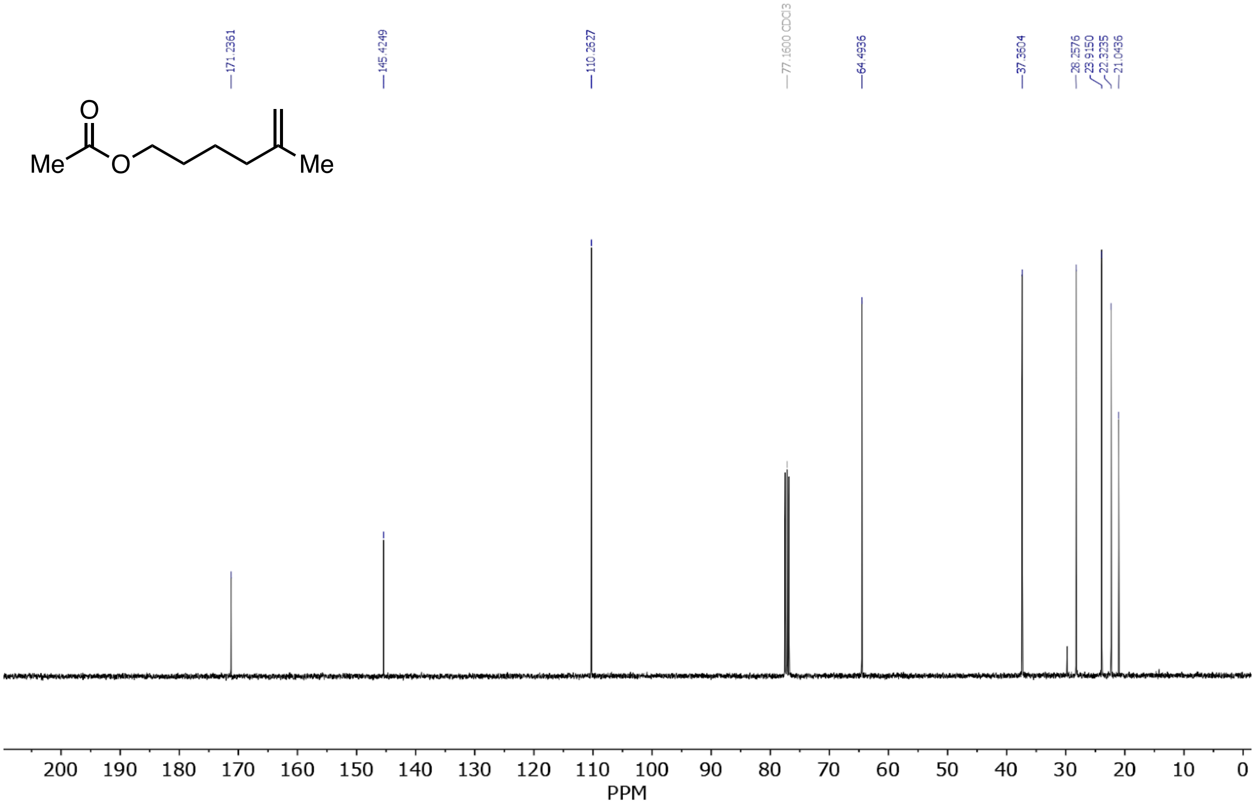

^{1}H NMR (400 MHz, $CDCl_3$) of 2-(5-methylhex-5-en-1-yl)isoindoline-1,3-dione (1j)

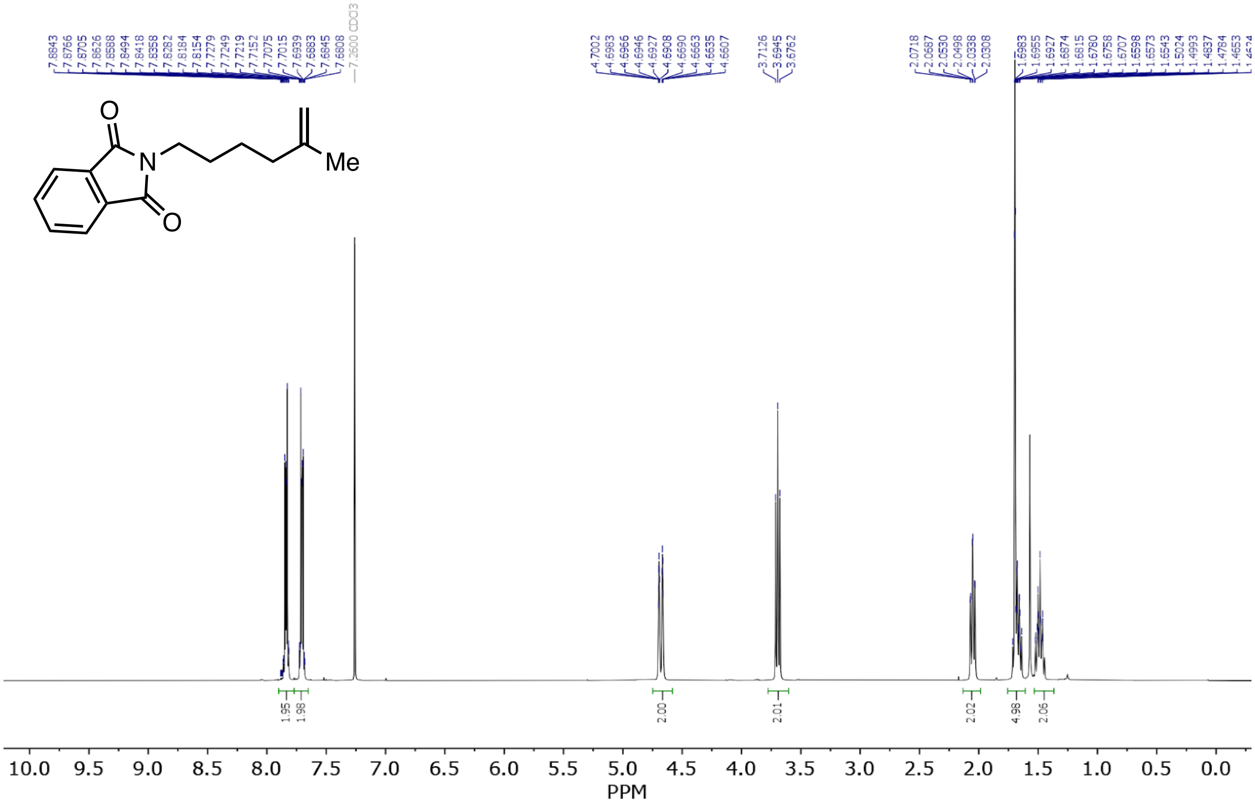

^{13}C{H} NMR (101 MHz, $CDCl_3$) of 2-(5-methylhex-5-en-1-yl)isoindoline-1,3-dione (1j)

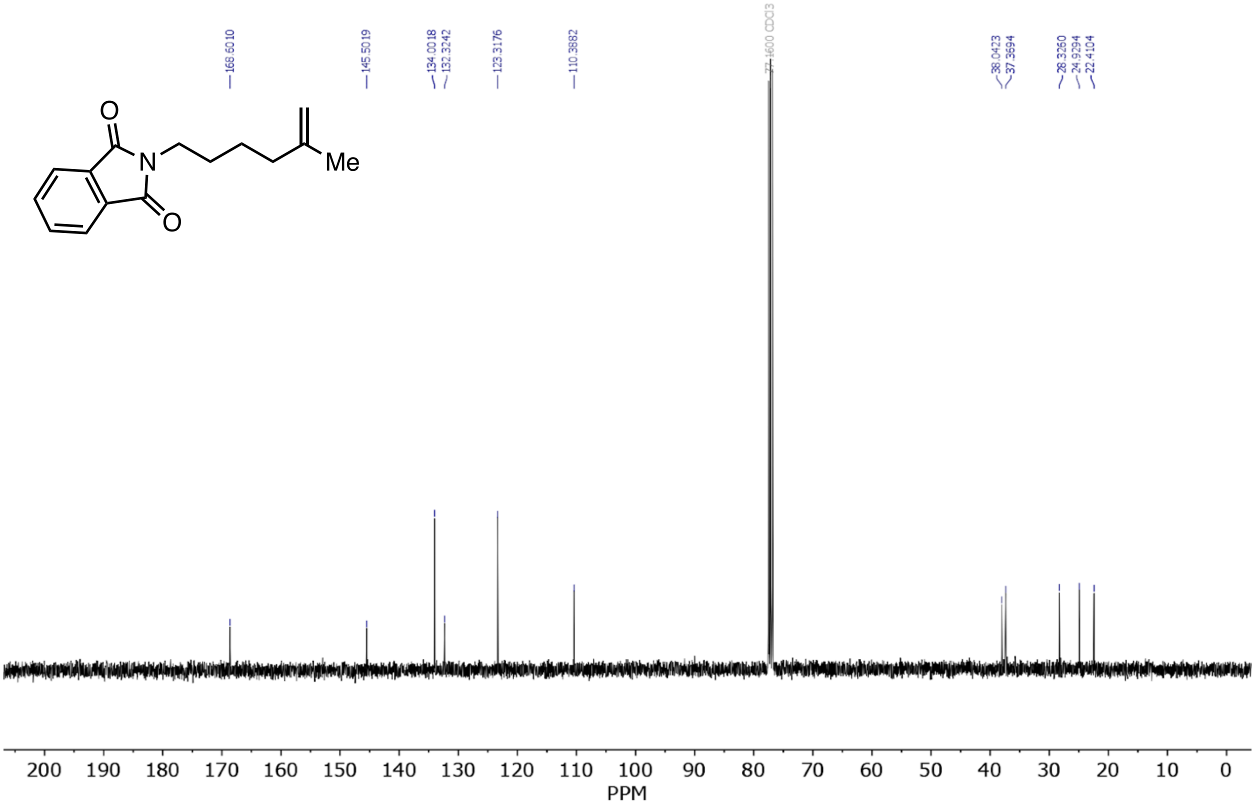

^{1}H NMR (400 MHz, CDCl$_3$) of 4-methyl-*N*-(5-methylhex-5-en-1-yl)benzenesulfonamide (1k)

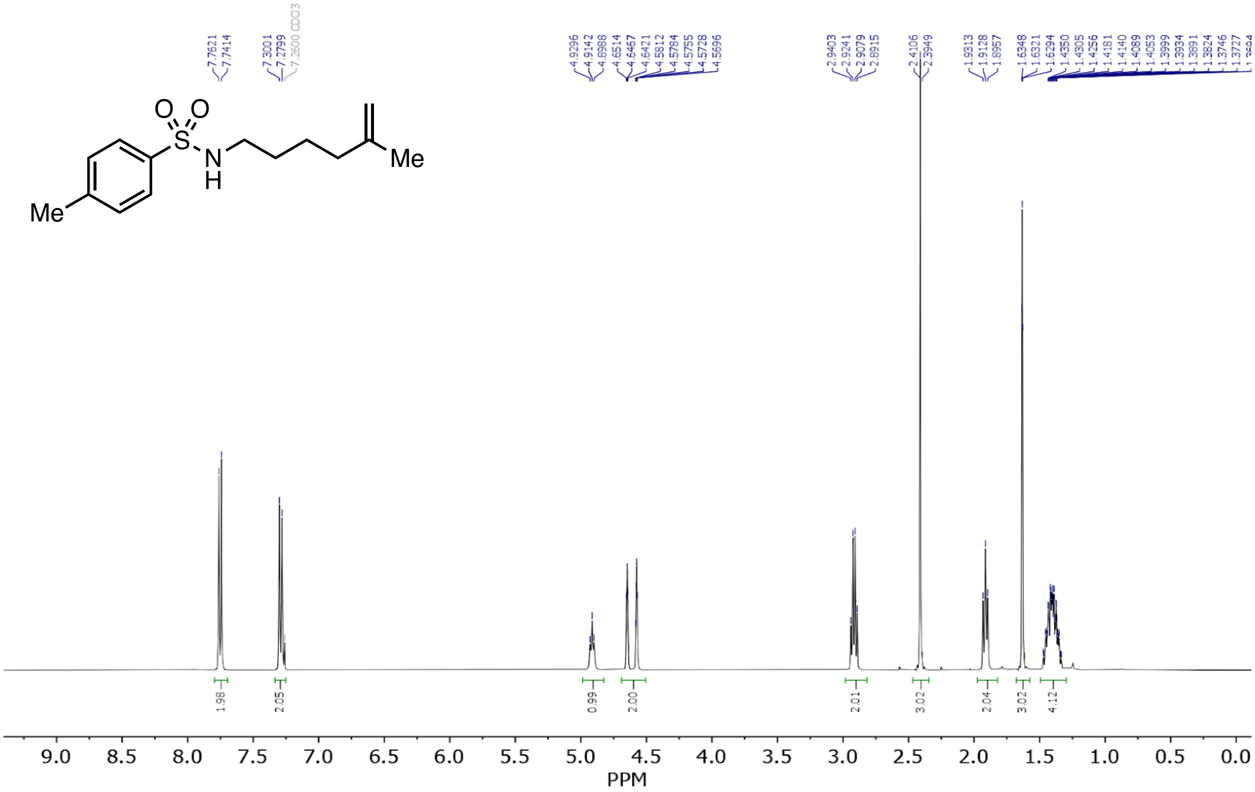

^{13}C{H} NMR (101 MHz, CDCl$_3$) of 4-methyl-*N*-(5-methylhex-5-en-1-yl)benzenesulfonamide (1k)

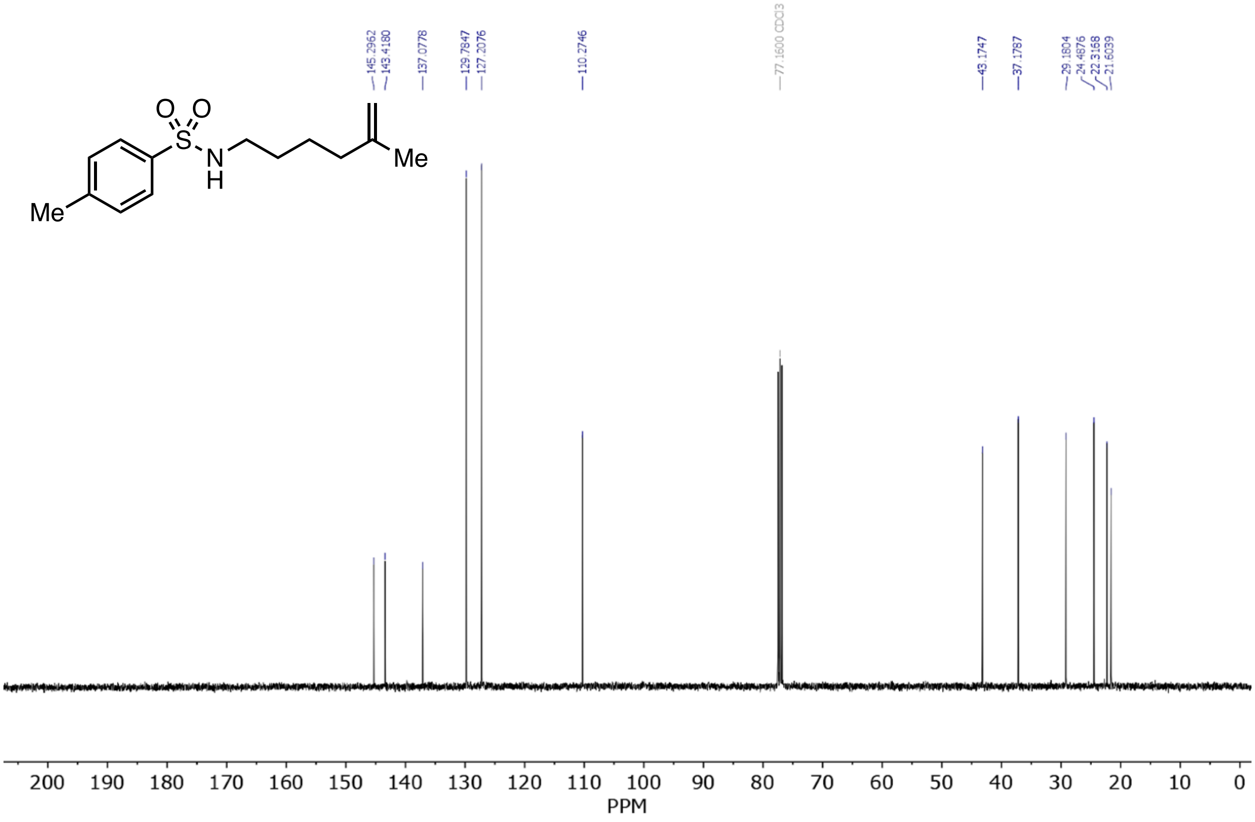

^{1}H NMR (500 MHz, $CDCl_3$) of 2-(4-phenylbutan-2-yl)-1,3-dioxolane (2a)

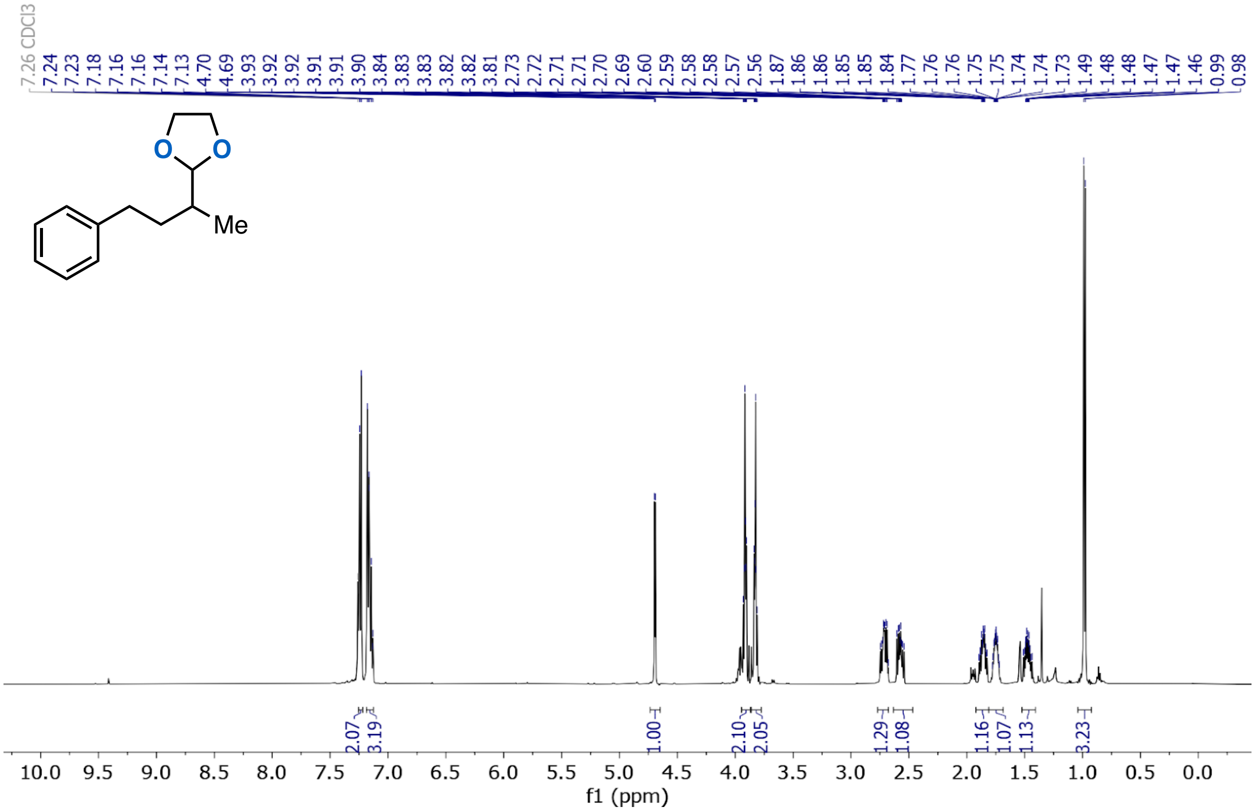

^{13}C{H} NMR (126 MHz, $CDCl_3$) of 2-(4-phenylbutan-2-yl)-1,3-dioxolane (2a)

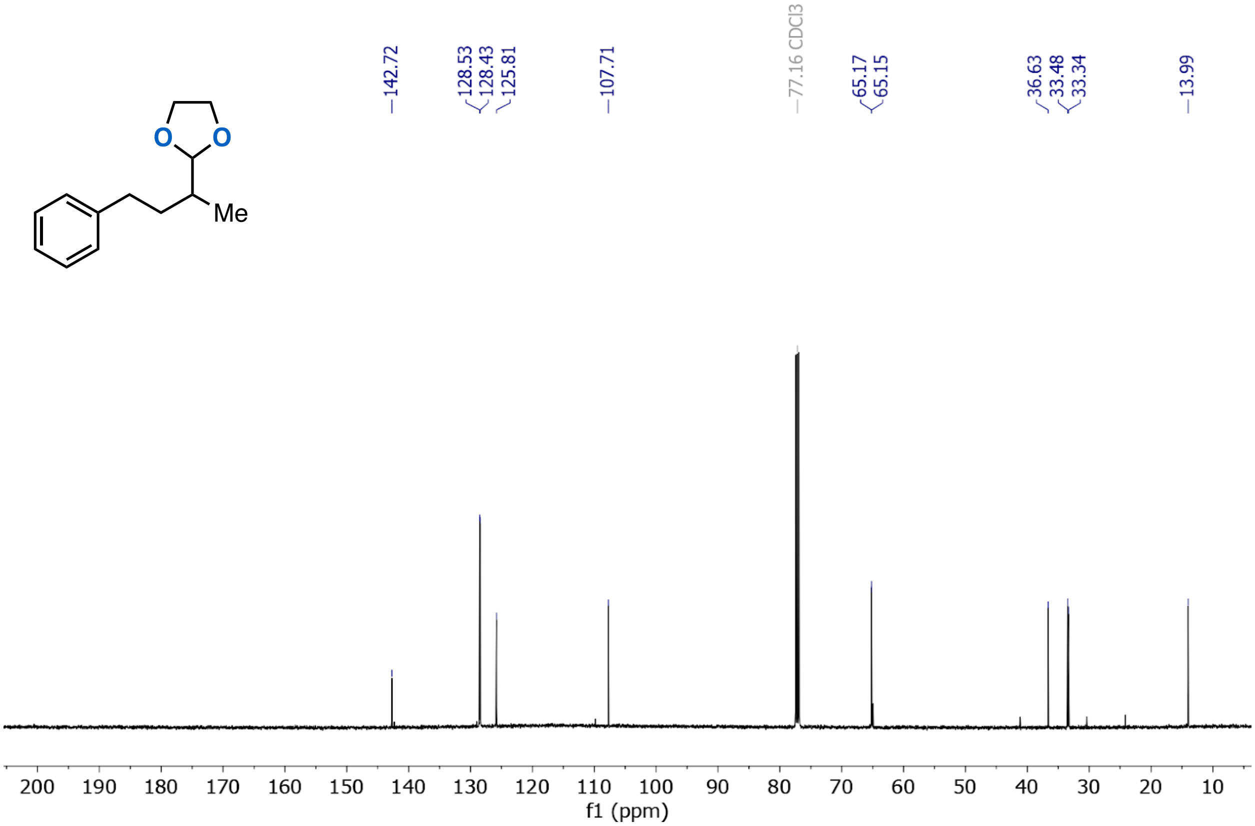

^{1}H NMR (400 MHz, $CDCl_3$) of 2-heptyl-4,4,5,5-tetramethyl-1,3-dioxolane (2b)

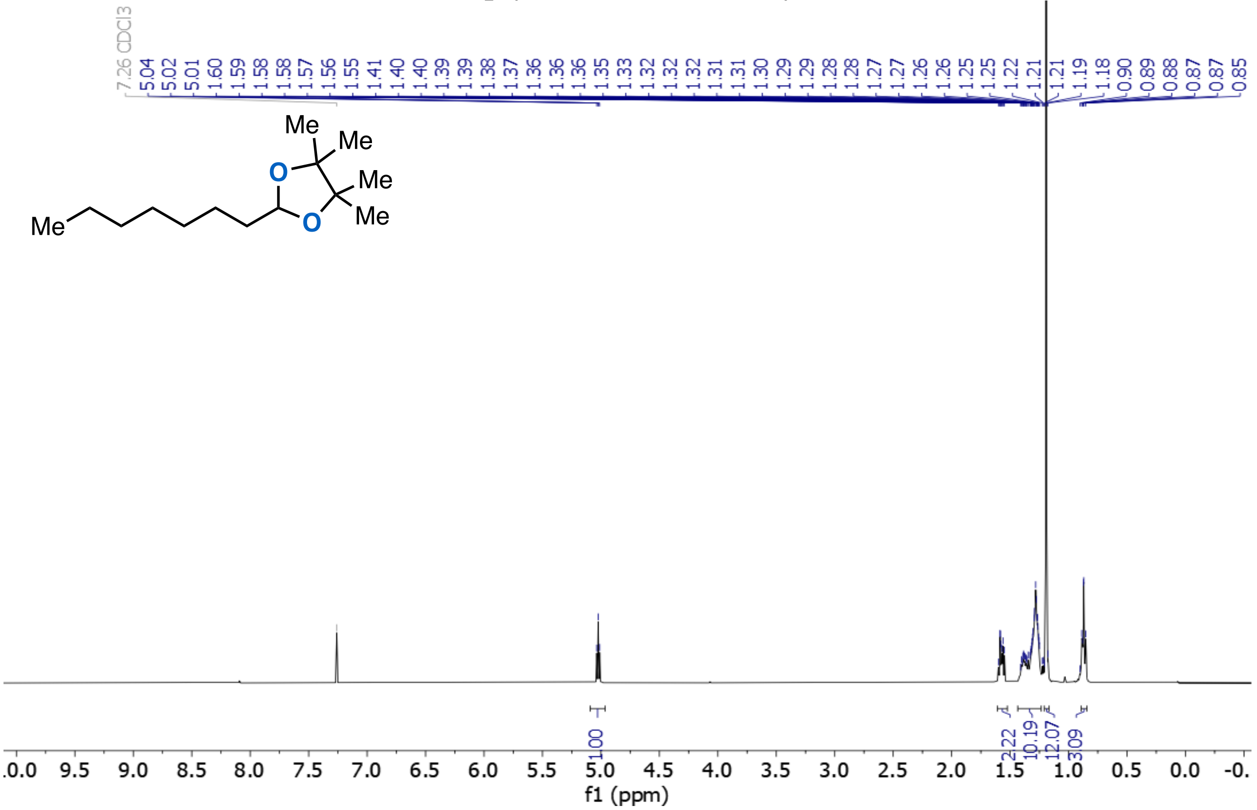

$^{13}C\{H\}$ NMR (101 MHz, $CDCl_3$) of 2-heptyl-4,4,5,5-tetramethyl-1,3-dioxolane (2b)

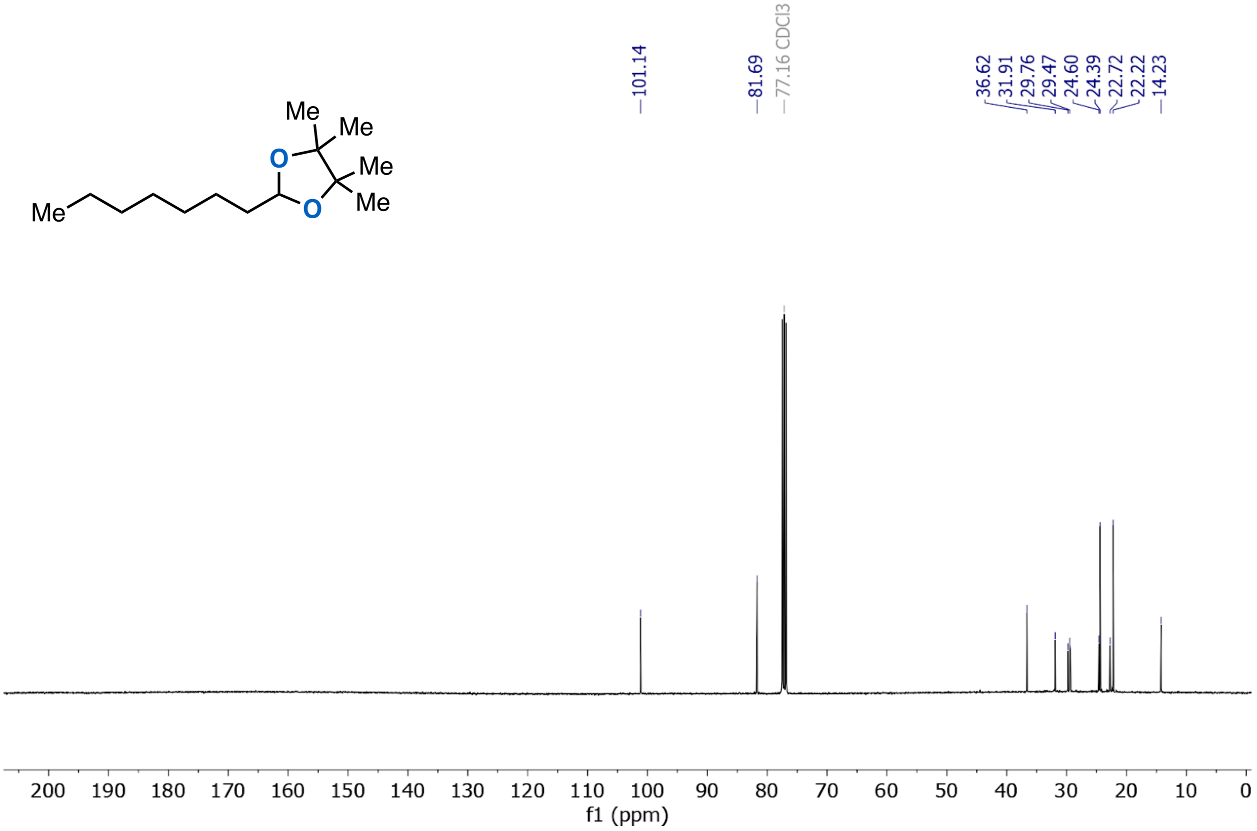

^{1}H NMR (500 MHz, $CDCl_3$) of 7-(1,3-dioxolan-2-yl)heptyl acetate (2c)

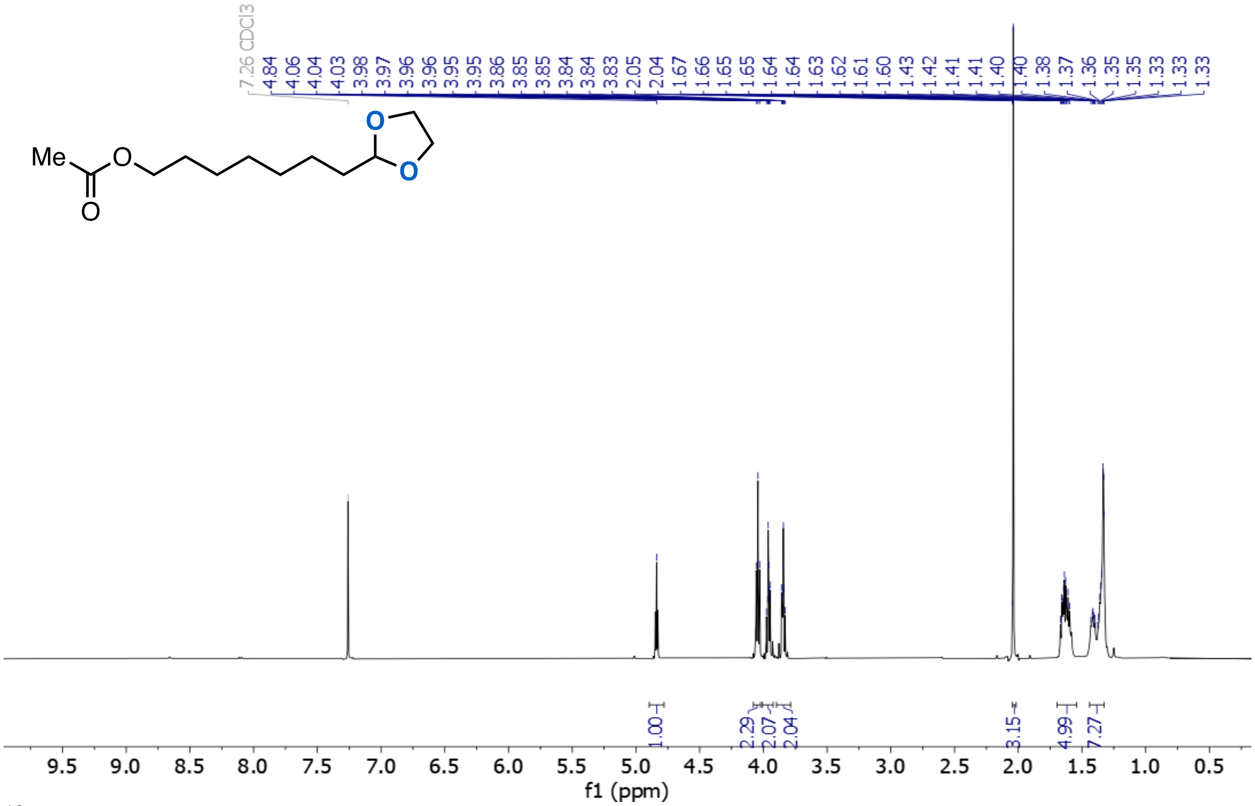

^{13}C{H} NMR (126 MHz, $CDCl_3$) of 7-(1,3-dioxolan-2-yl)heptyl acetate (2c)

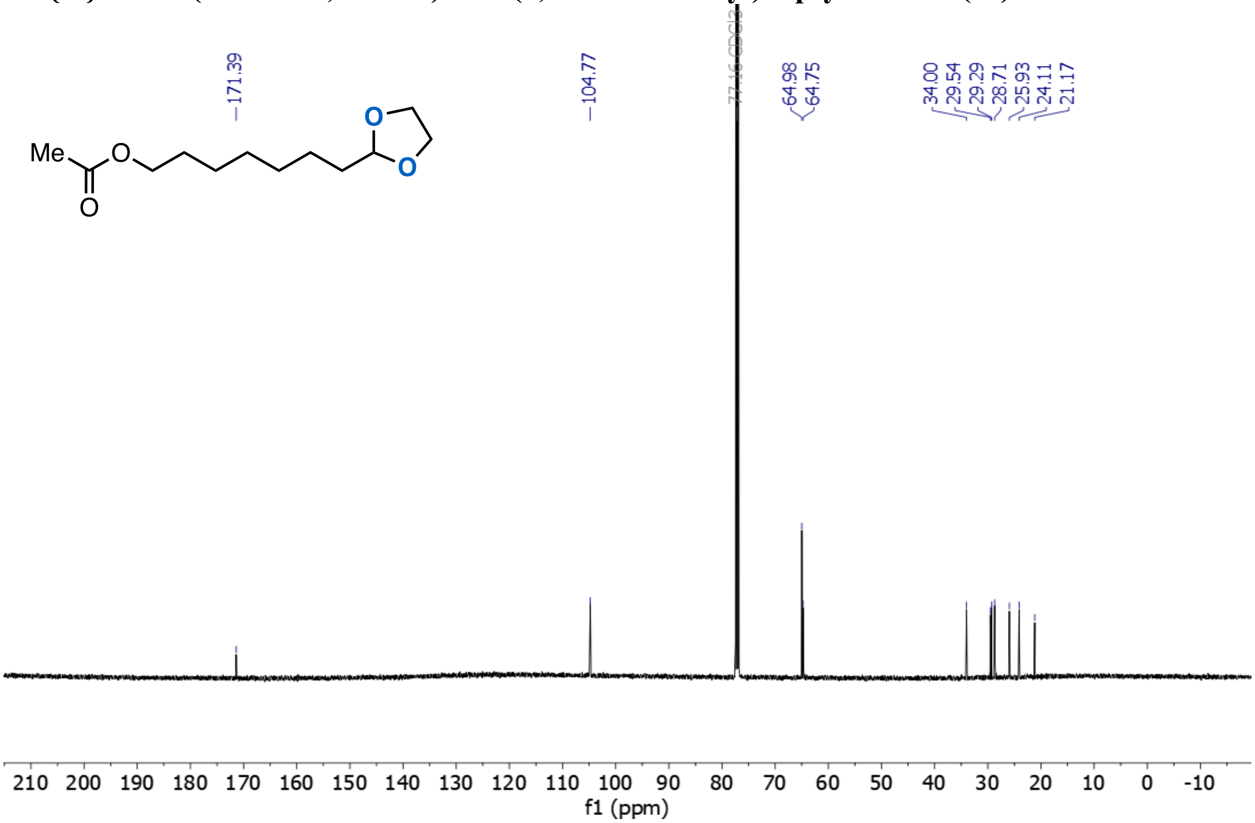

^{1}H NMR (500 MHz, CDCl$_3$) of *S*-(7-(1,3-dioxolan-2-yl)heptyl) ethanethioate (2d)

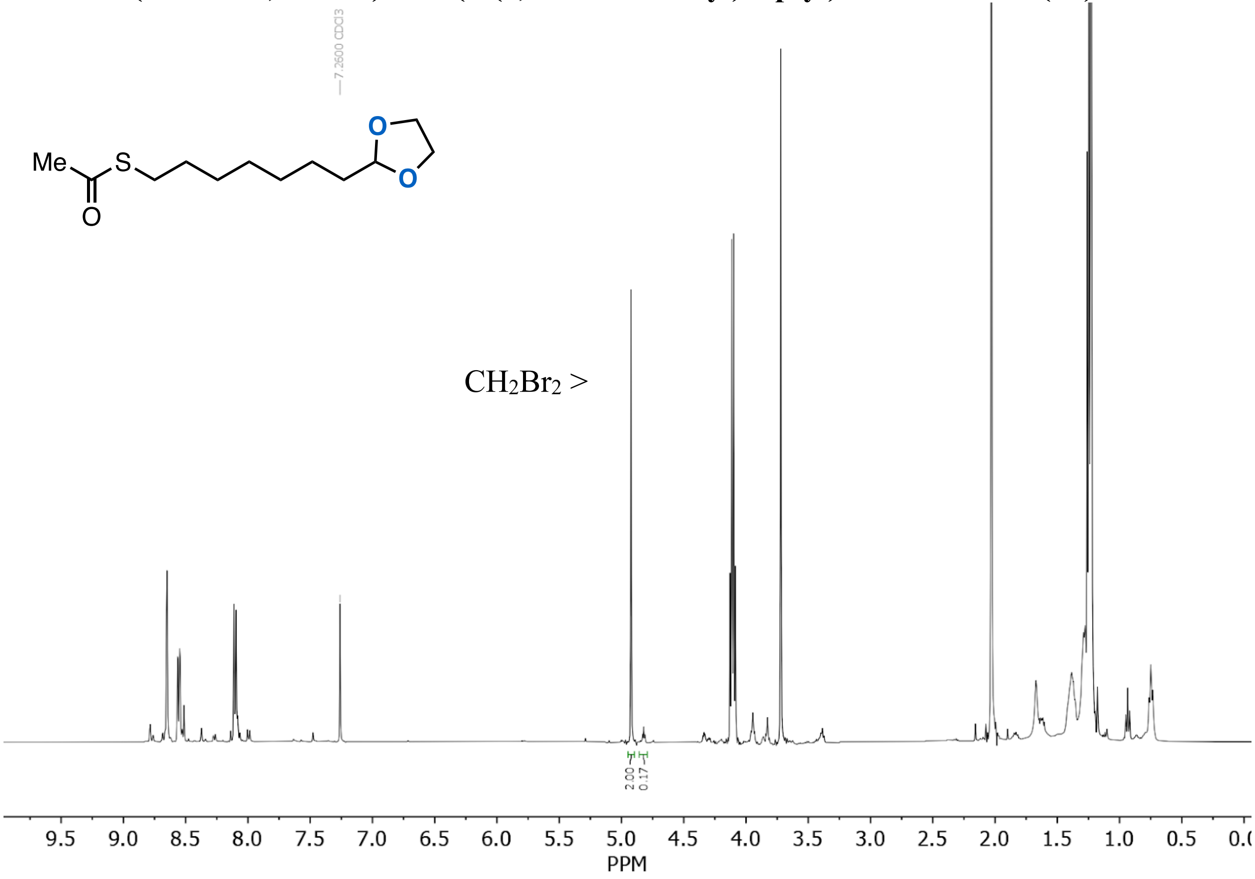

^{1}H NMR (500 MHz, $CDCl_3$) of 2-(7-(1,3-dioxolan-2-yl)heptyl)-4,4,5,5-tetramethyl-1,3,2-dioxaborolane (2e)

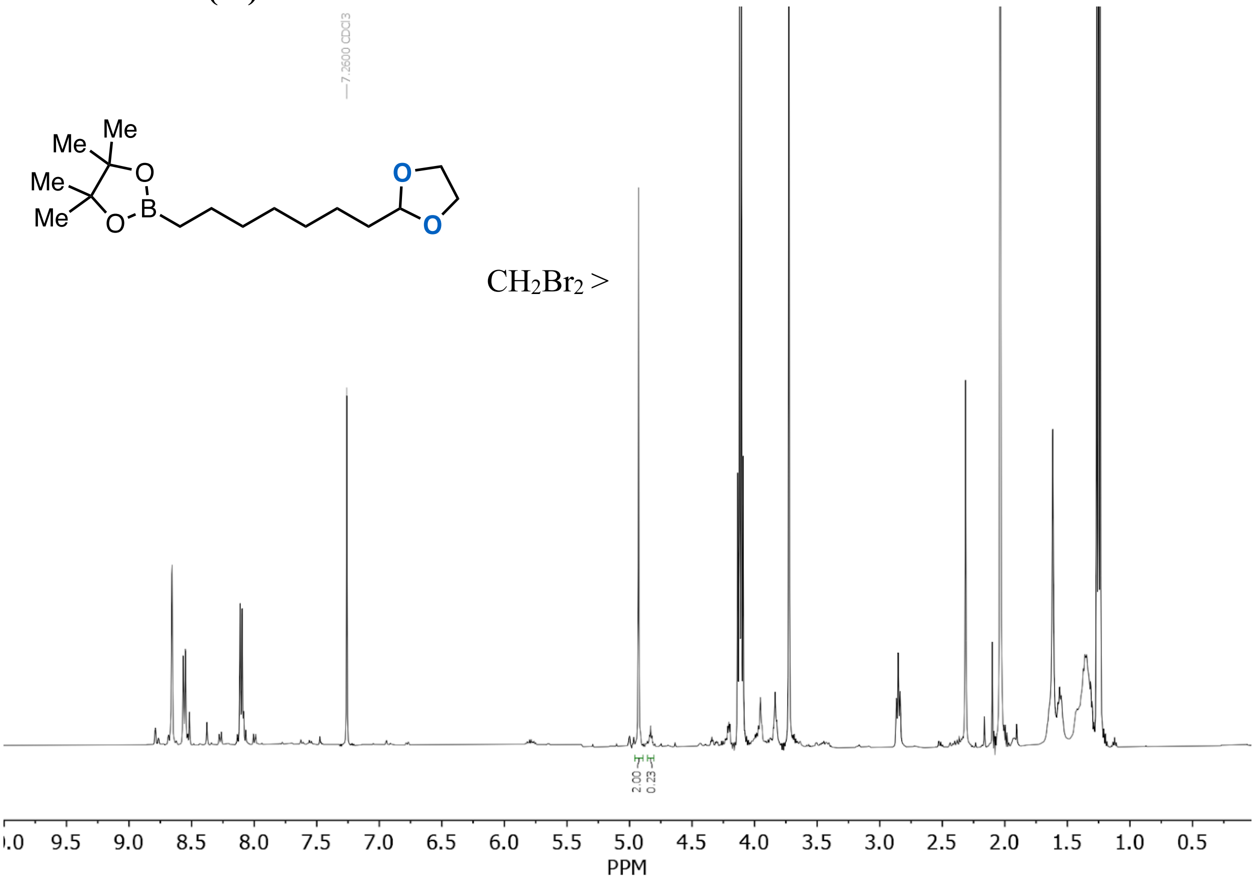

^{1}H NMR (500 MHz, $CDCl_3$) of (4,4-dimethoxybutyl)benzen and (3,3-dimethoxypropyl)benzene (2f + 2f')

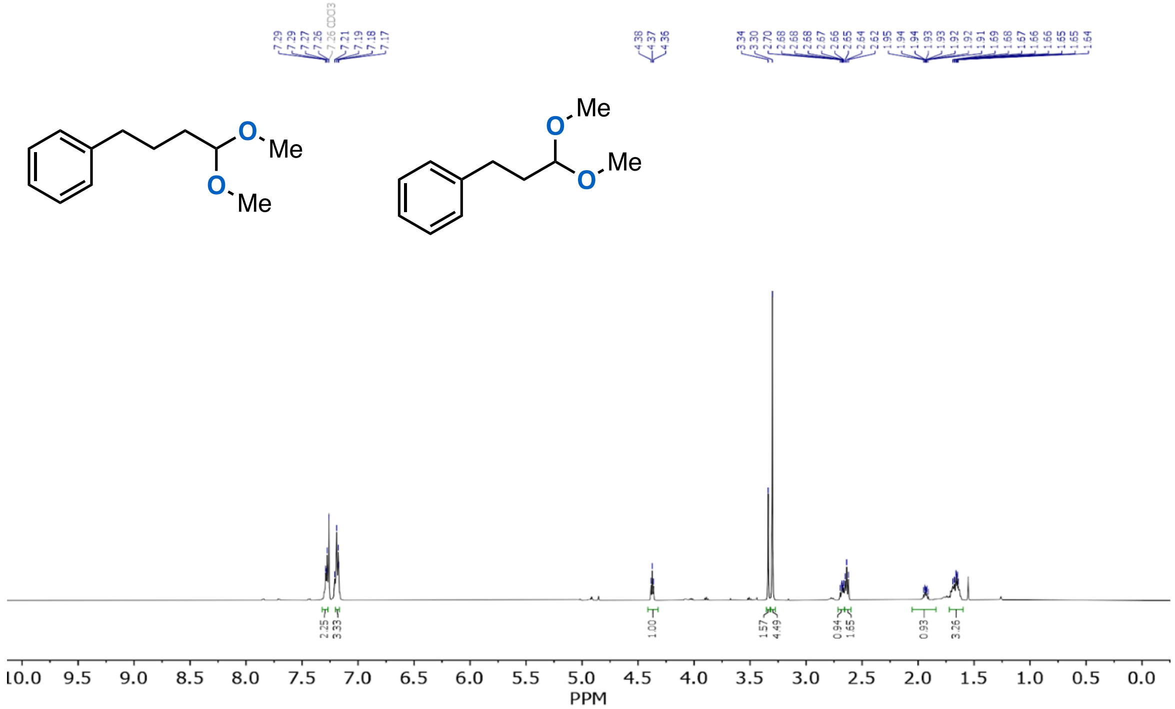

^{13}C{H} NMR (126 MHz, $CDCl_3$) of (4,4-dimethoxybutyl)benzene and (3,3-dimethoxypropyl)benzene (2f + 2f')

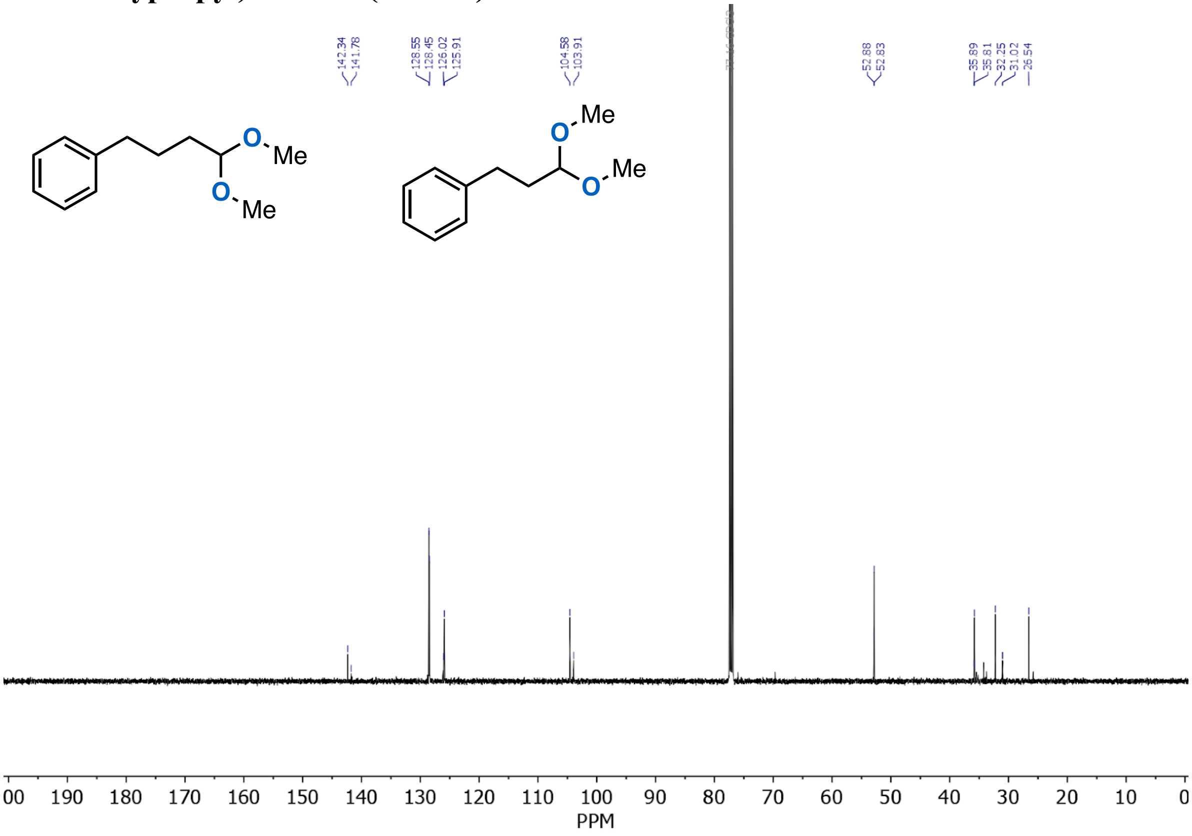

^{1}H NMR (500 MHz, $CDCl_3$) of 4-(3-methyl-4-oxobutyl)phenyl benzoate (2g)

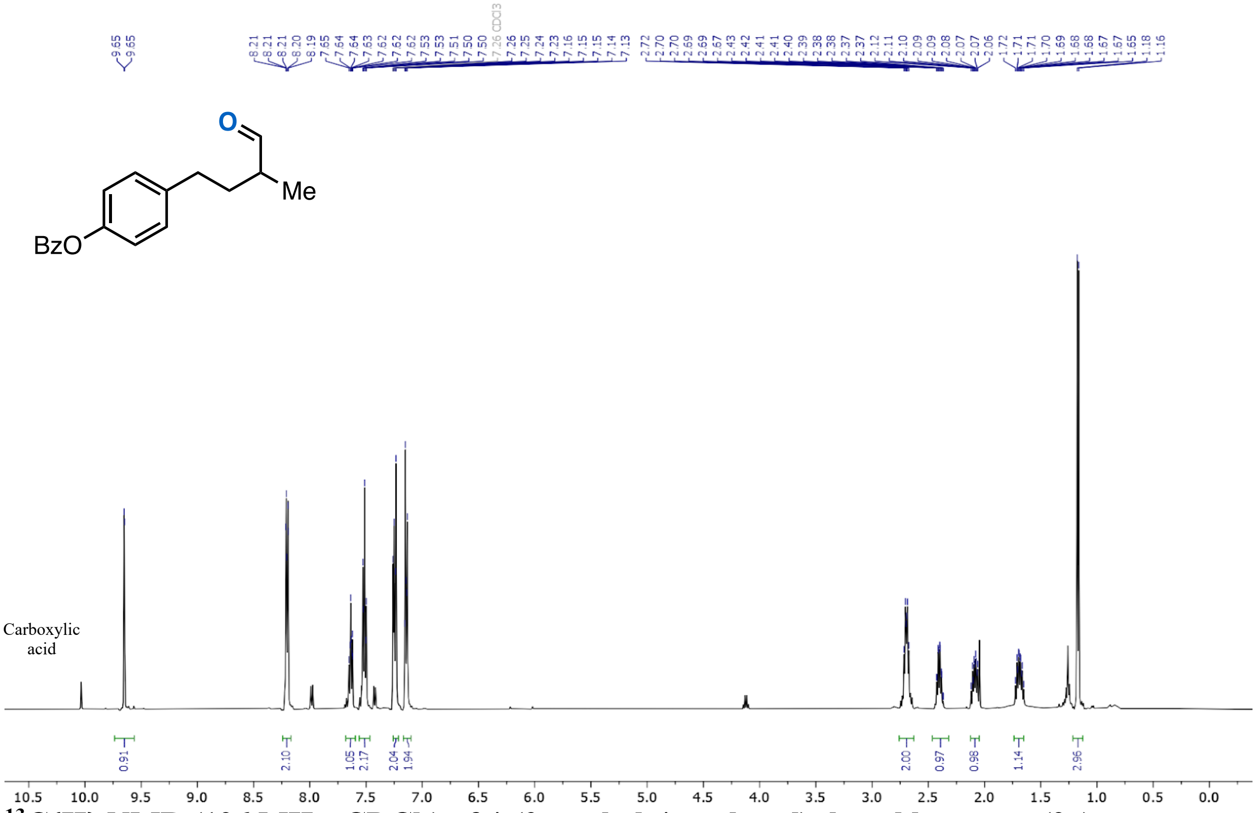

$^{13}C\{H\}$ NMR (126 MHz, $CDCl_3$) of 4-(3-methyl-4-oxobutyl)phenyl benzoate (2g)

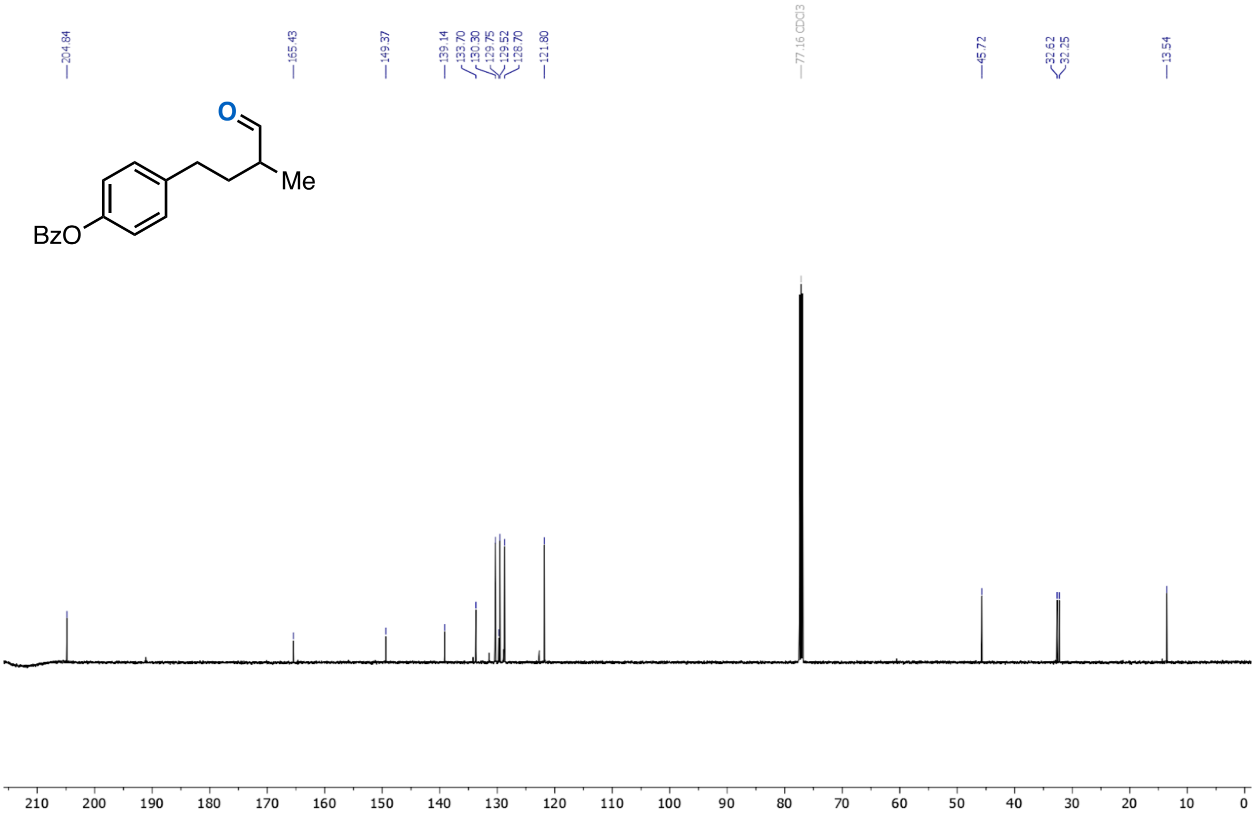

^{1}H NMR (400 MHz, $CDCl_3$) of 5-methyl-6-oxohexyl acetate (2h)

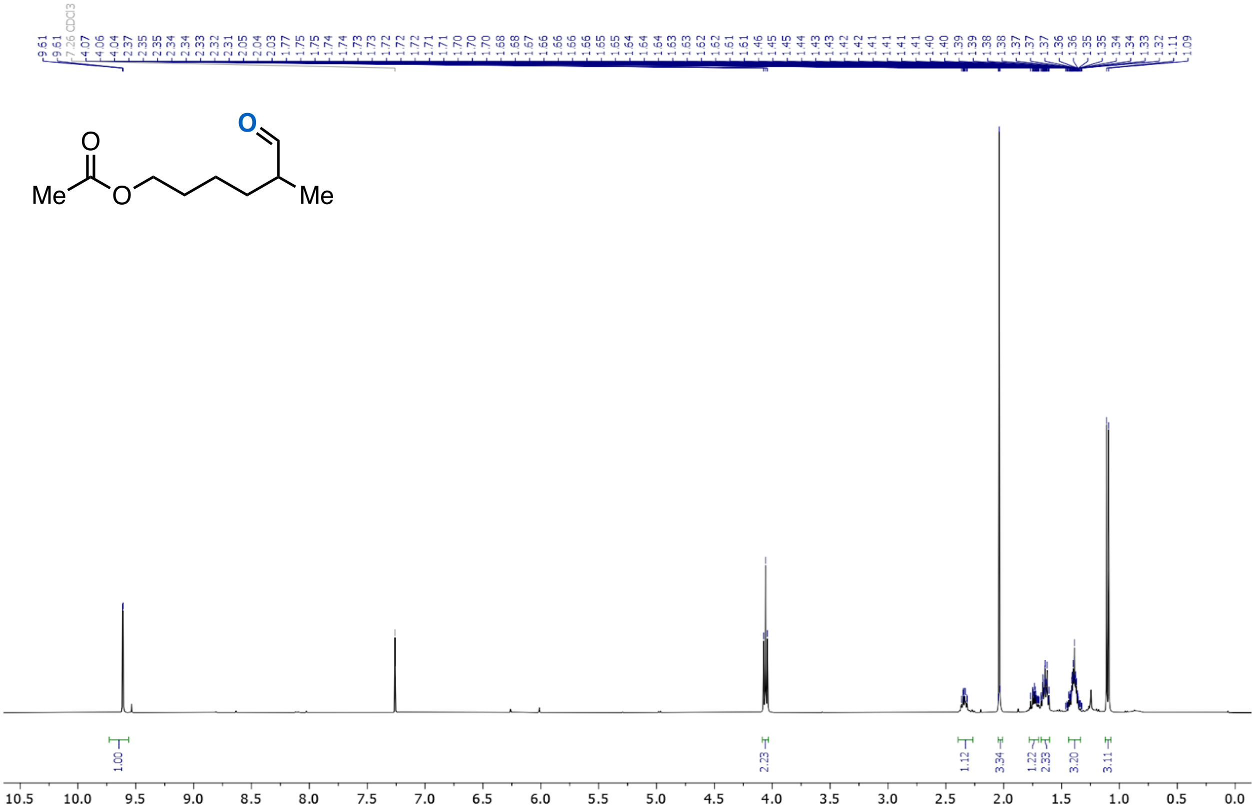

$^{13}C\{H\}$ NMR (101 MHz, $CDCl_3$) of 5-methyl-6-oxohexyl acetate (2h)

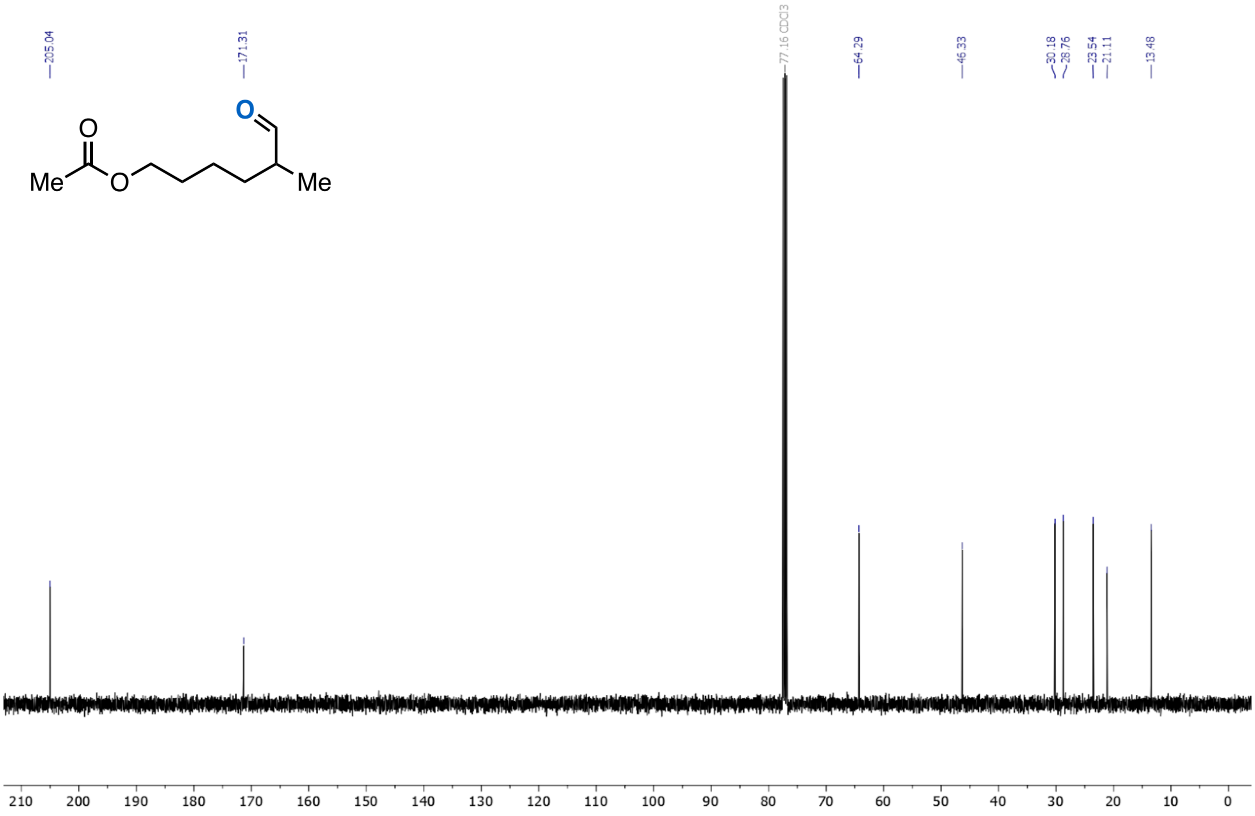

^{1}H NMR (400 MHz, $CDCl_3$) of 6-methyl-7-oxoheptanenitrile (2i)

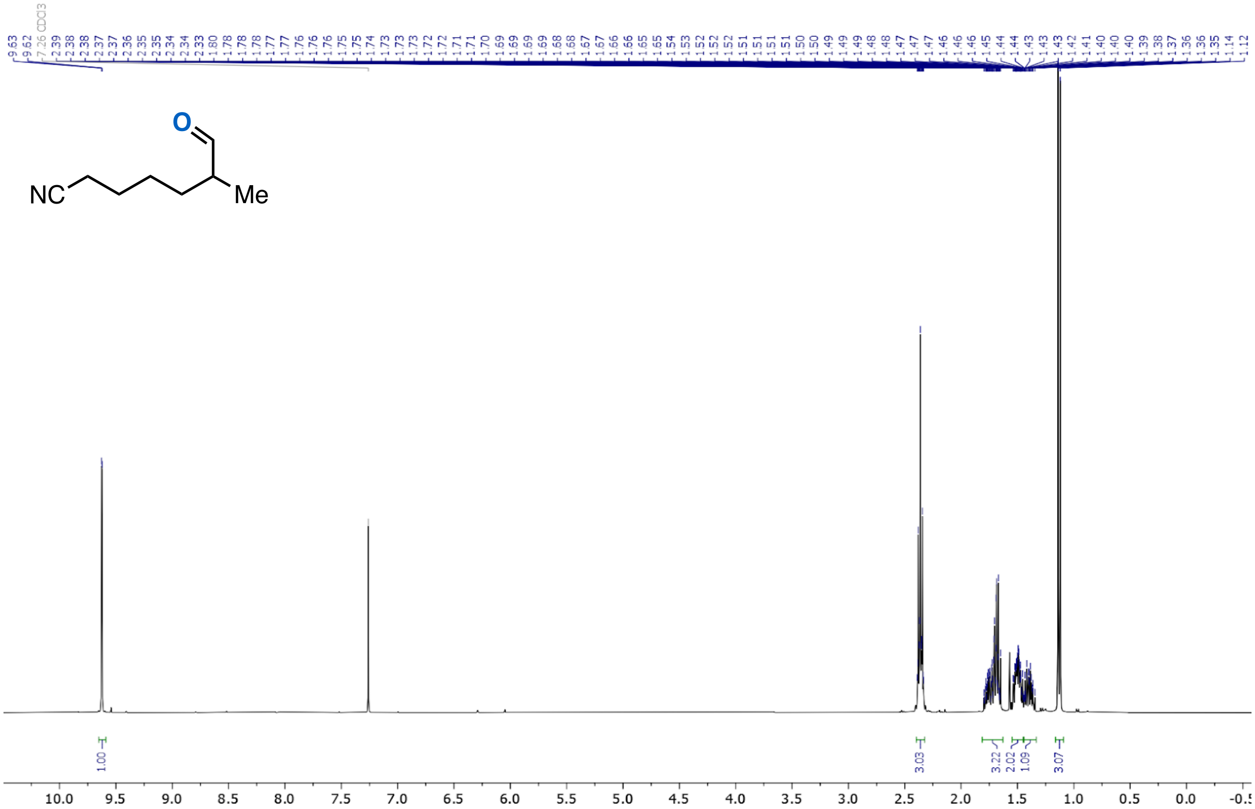

^{13}C{H} NMR (101 MHz, $CDCl_3$) of 6-methyl-7-oxoheptanenitrile (2i)

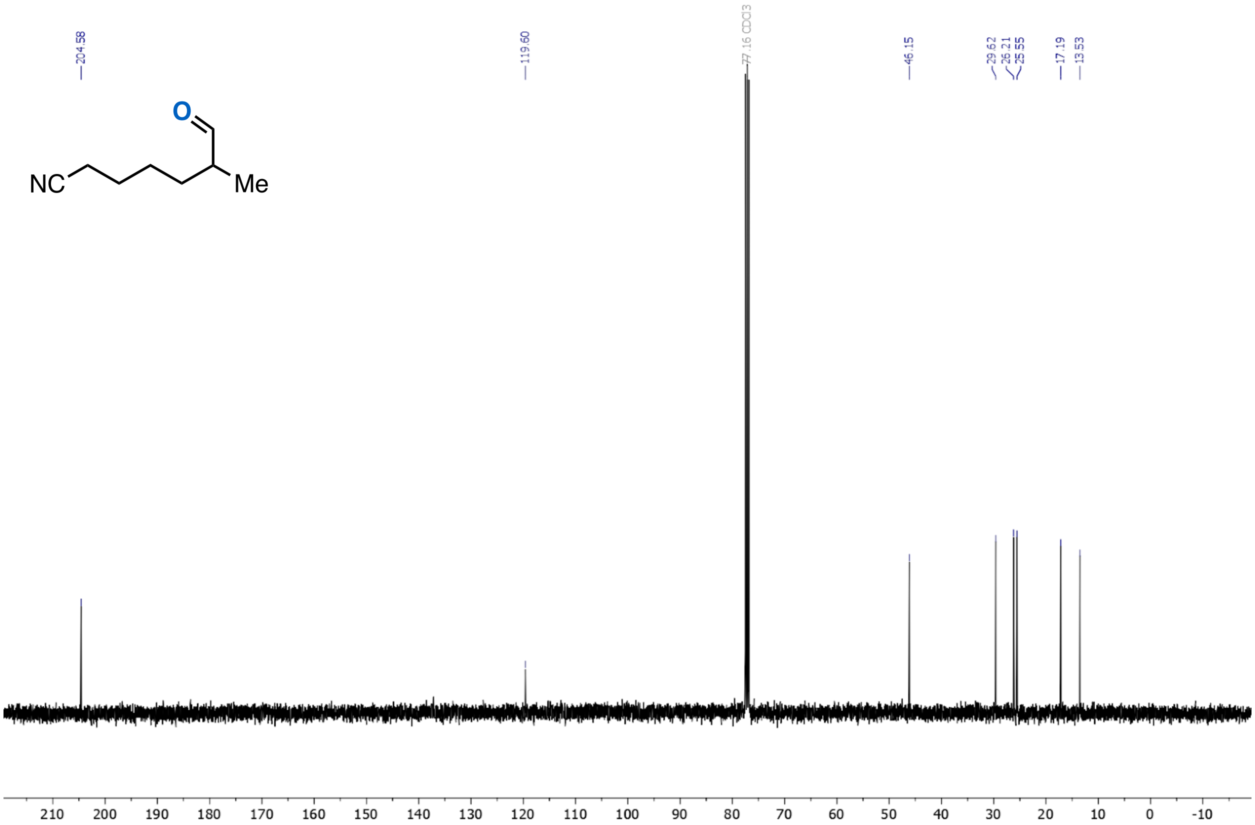

^{1}H NMR (400 MHz, $CDCl_3$) of 6-(1,3-dioxoisoindolin-2-yl)-2-methylhexanal (2j)

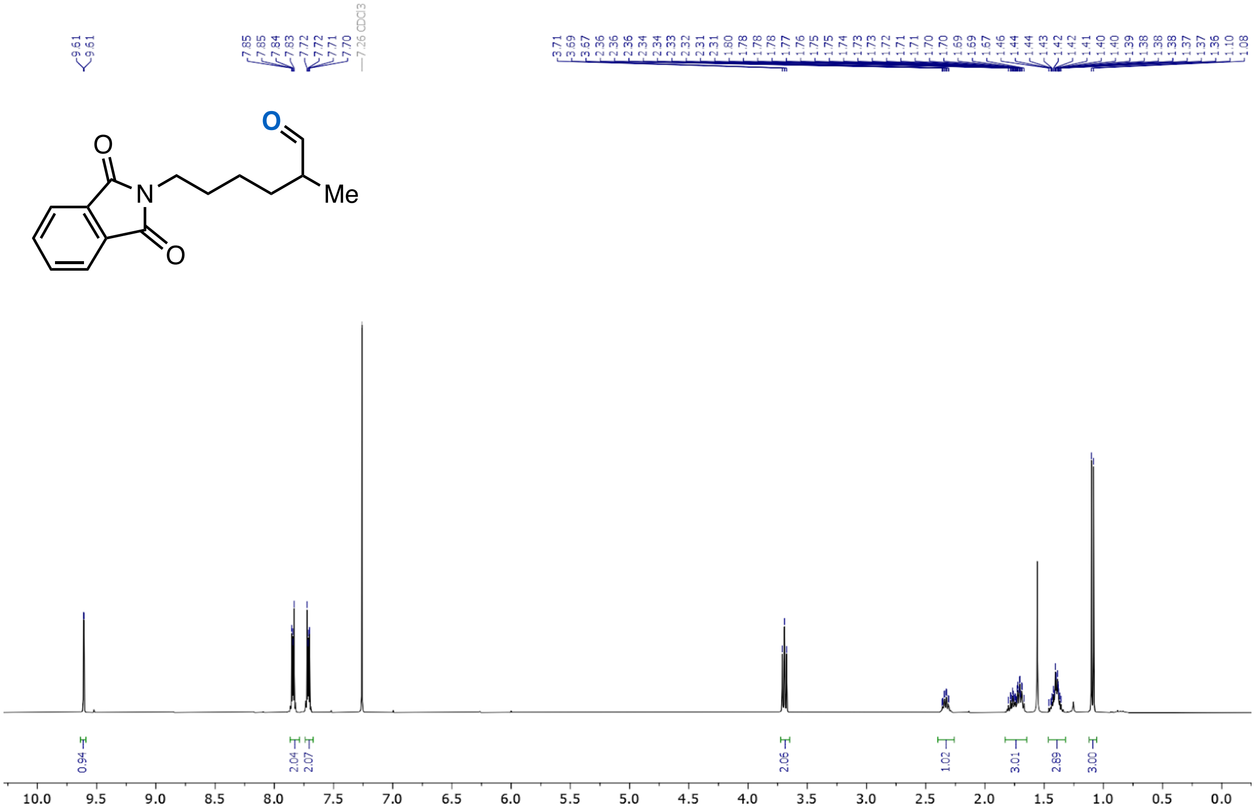

^{13}C{H} NMR (101 MHz, $CDCl_3$) of 6-(1,3-dioxoisoindolin-2-yl)-2-methylhexanal (2j)

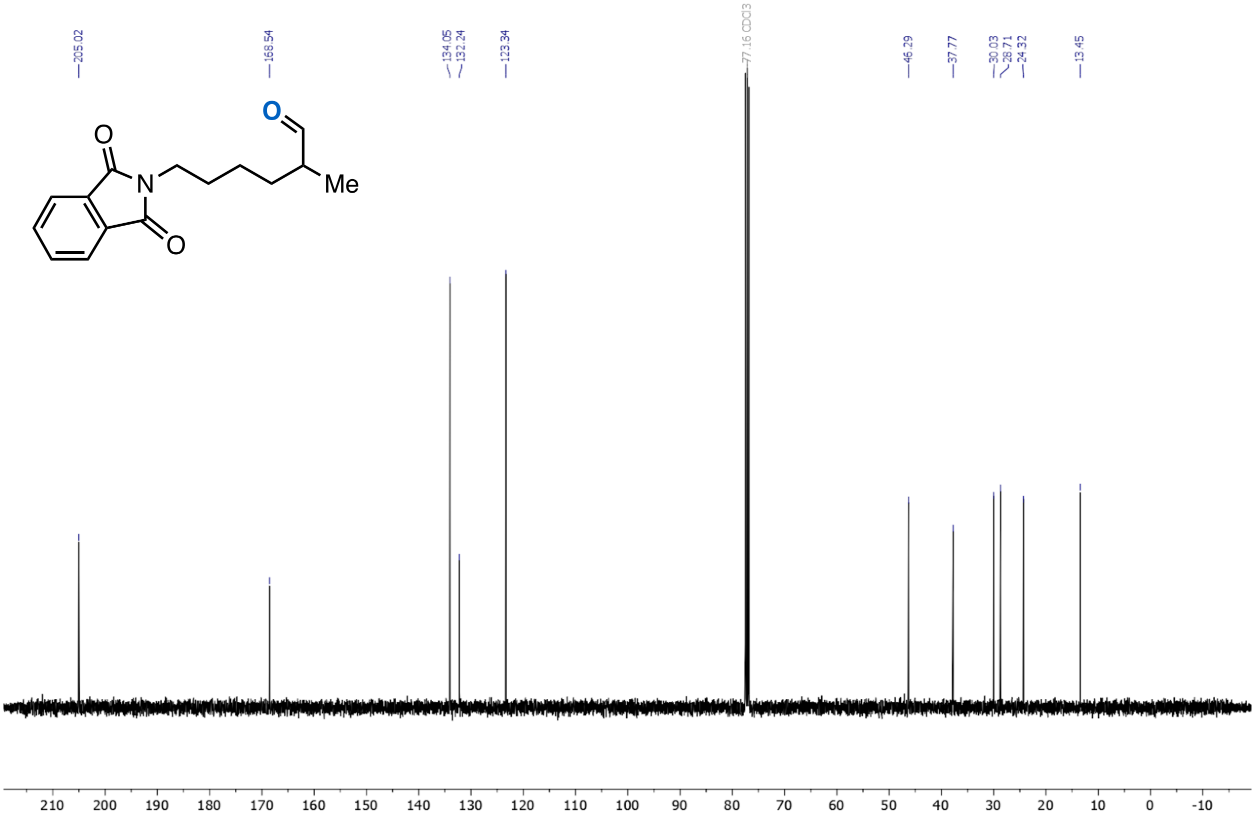

^{1}H NMR (500 MHz, $CDCl_3$) of 4-methyl-*N*-(5-methyl-6-oxohexyl)benzenesulfonamide (2k)

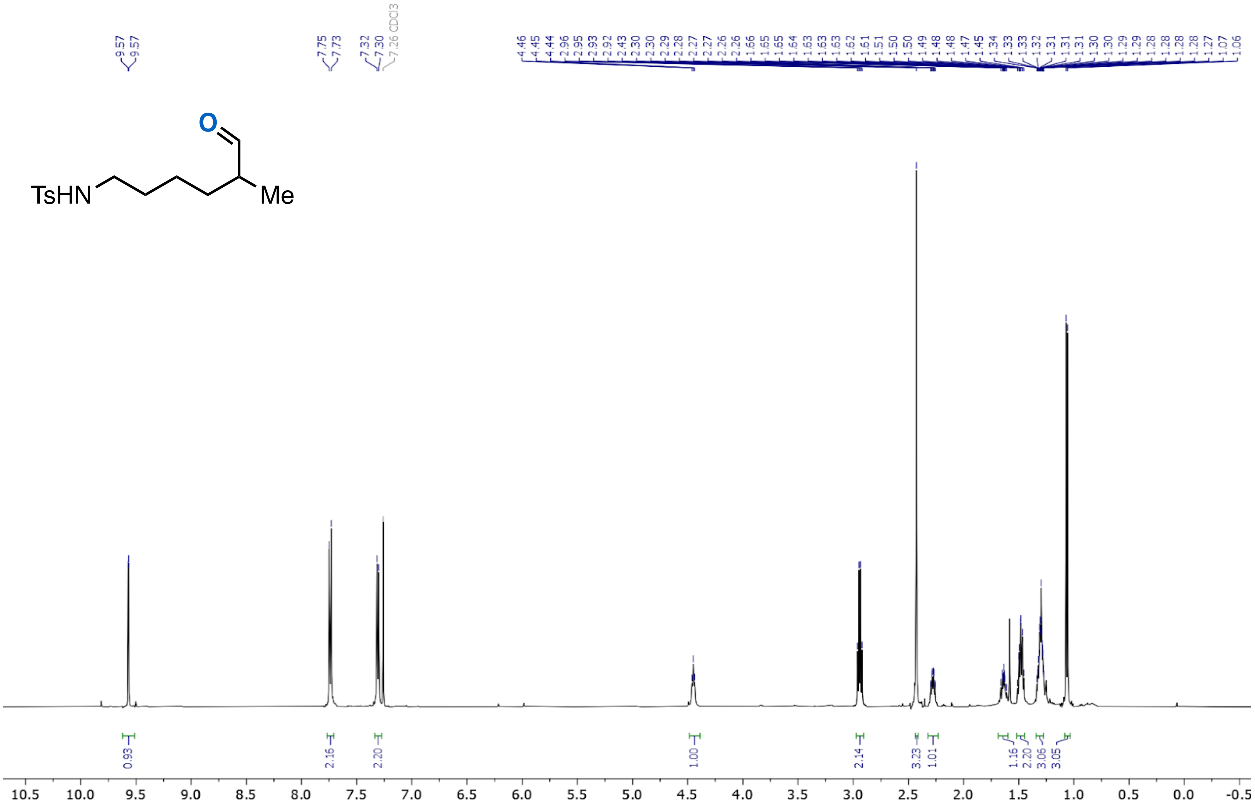

^{13}C{H} NMR (126 MHz, $CDCl_3$) of 4-methyl-*N*-(5-methyl-6-oxohexyl)benzenesulfonamide (2k)

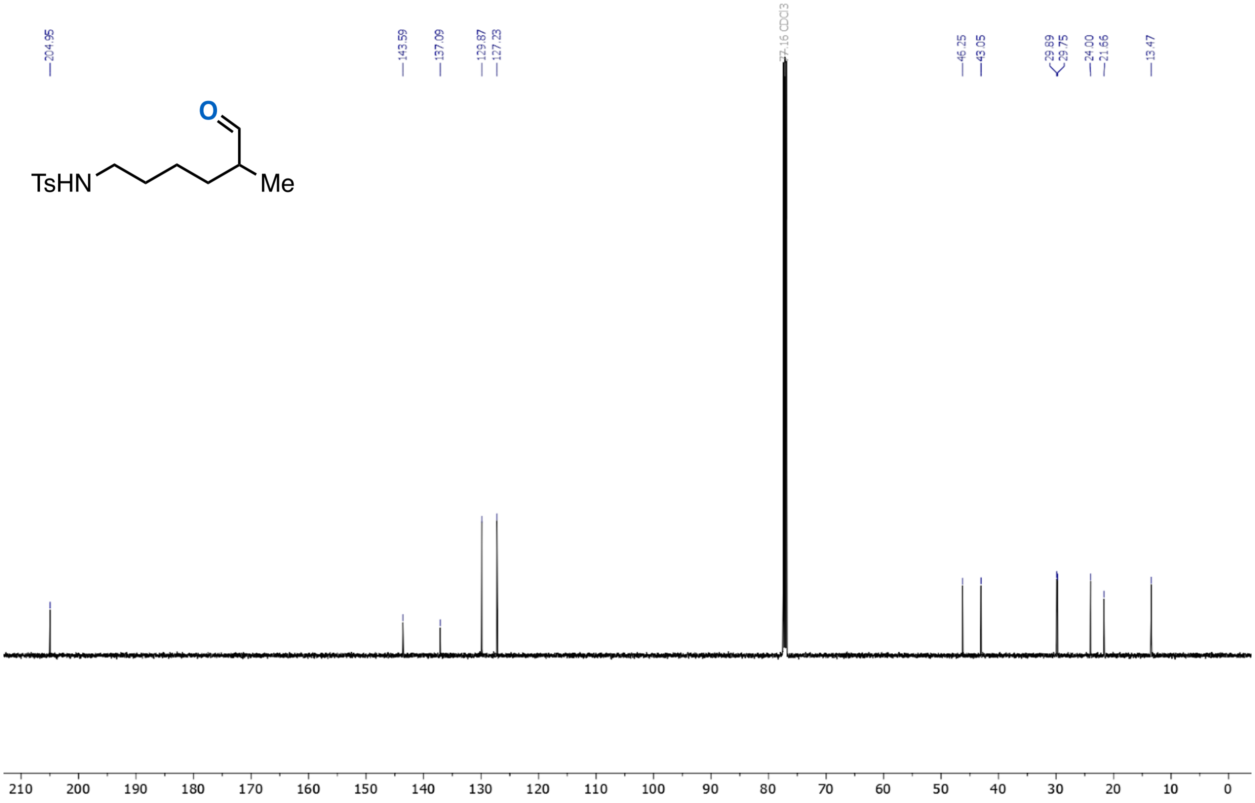

^{1}H NMR (500 MHz, $CDCl_3$) of 2-(6-chlorohexan-2-yl)-1,3-dioxolane (2l)

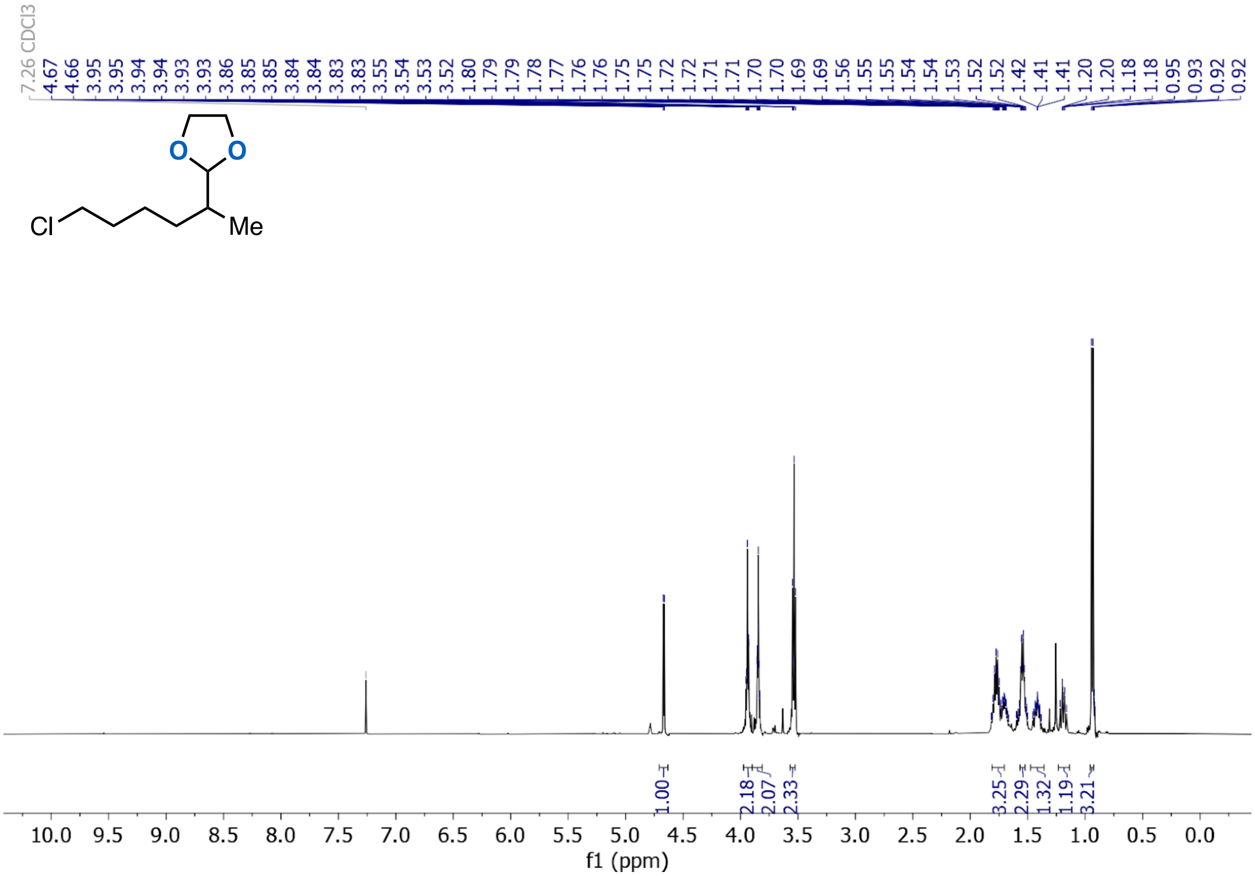

^{13}C{H} NMR (126 MHz, $CDCl_3$) of 2-(6-chlorohexan-2-yl)-1,3-dioxolane (2l)

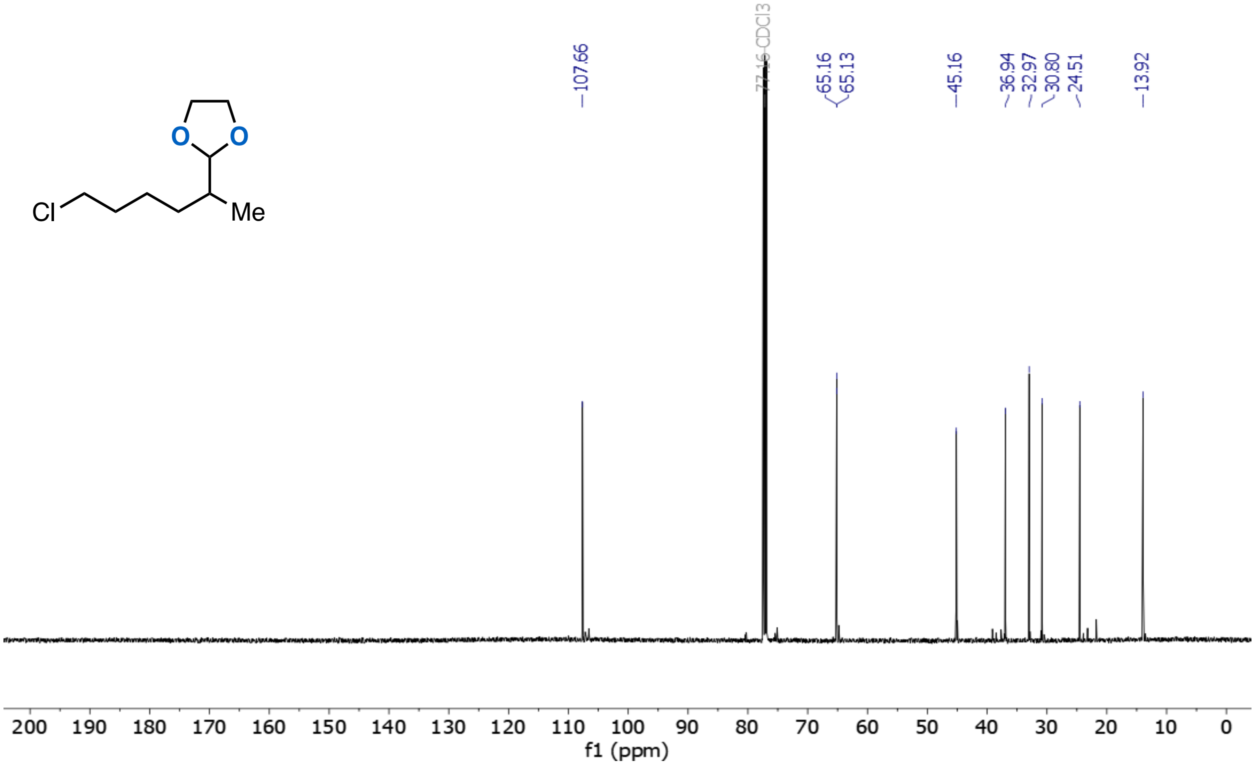

^{1}H NMR (400 MHz, $CDCl_3$) of 2-(decan-2-yl)-1,3-dioxolane (2m)

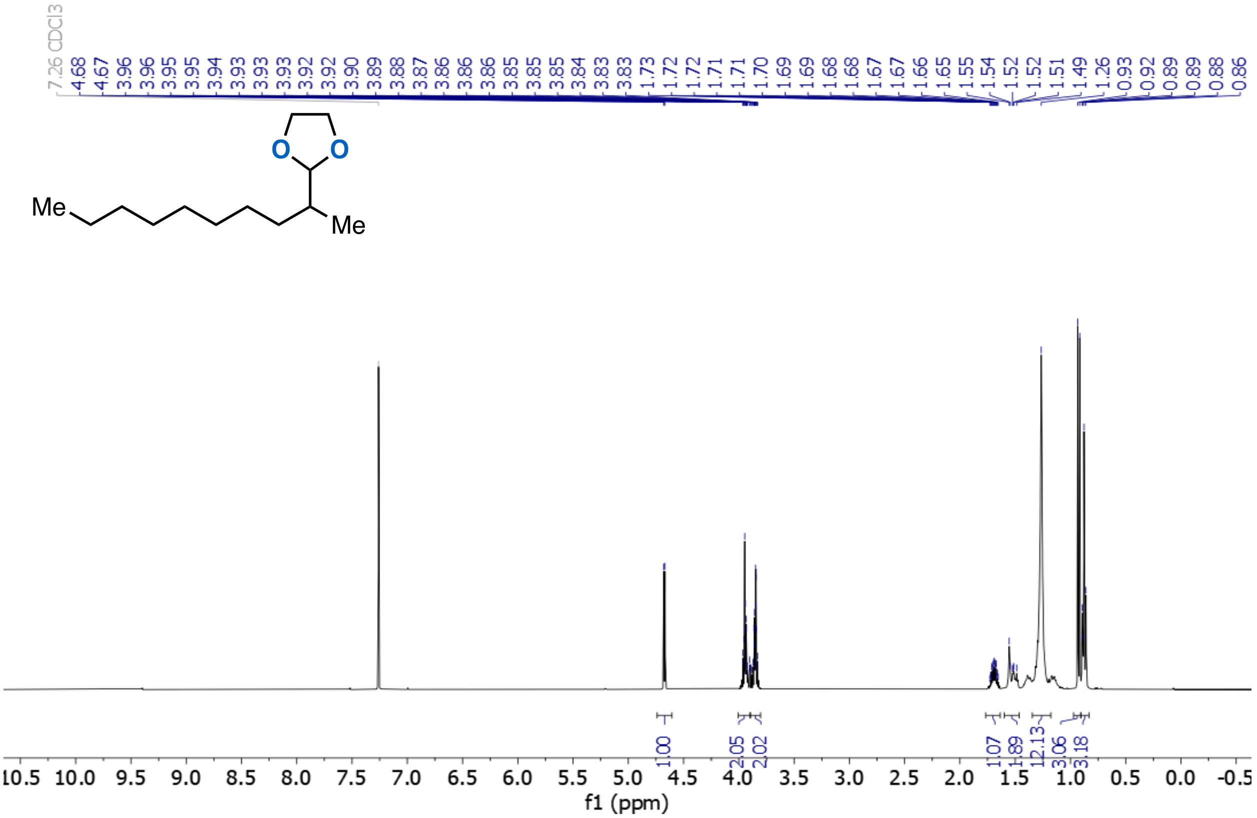

^{13}C{H} NMR (101 MHz, $CDCl_3$) of 2-(decan-2-yl)-1,3-dioxolane (2m)

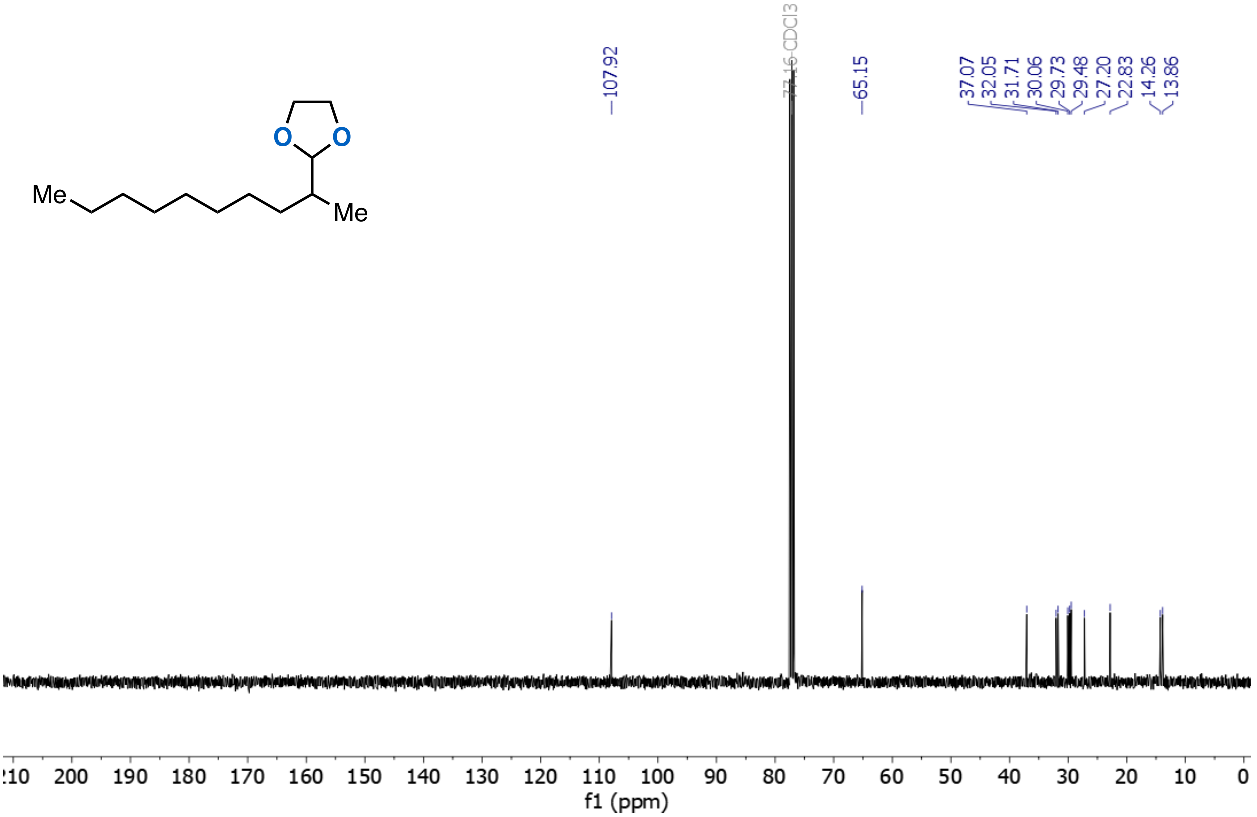

^{1}H NMR (500 MHz, $CDCl_3$) of 2-phenylcyclohexane-1-carbaldehyde (2n)

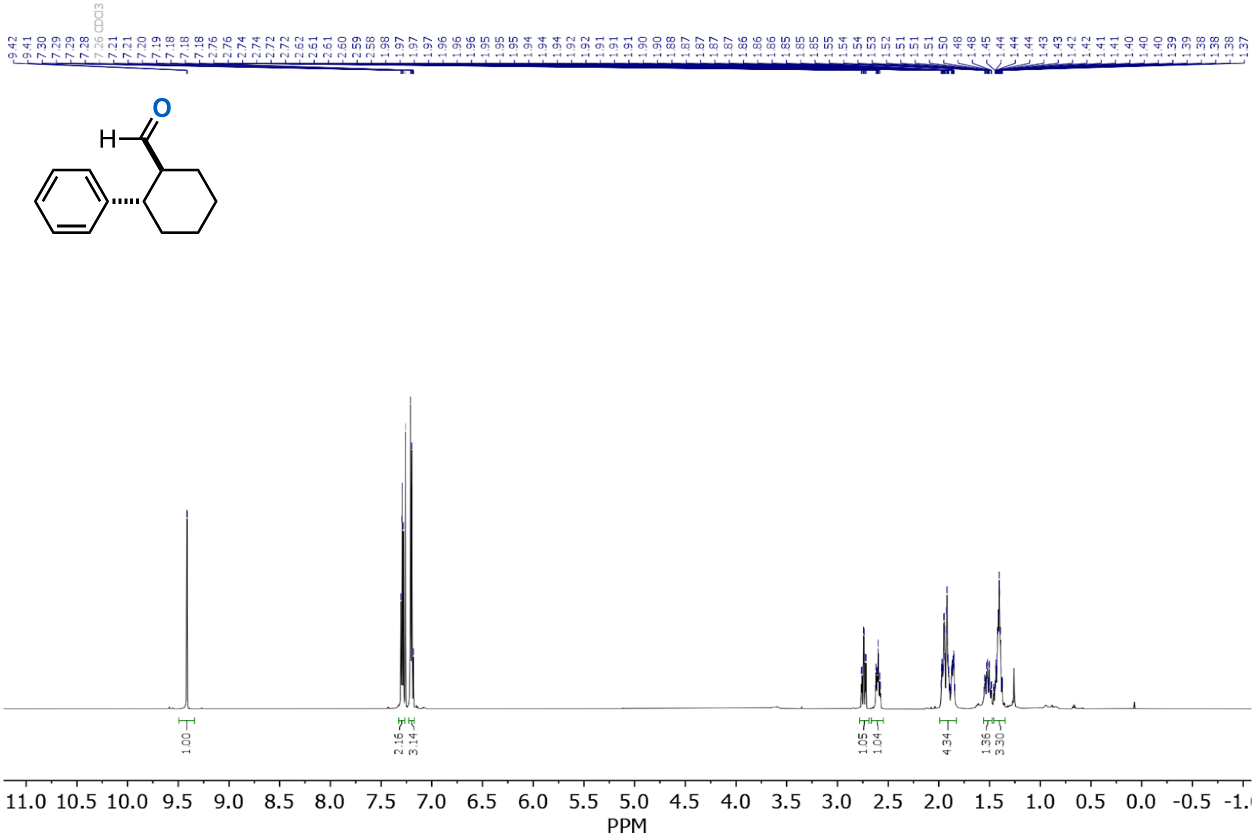

^{13}C{H} NMR (126 MHz, $CDCl_3$) of of 2-phenylcyclohexane-1-carbaldehyde (2n)

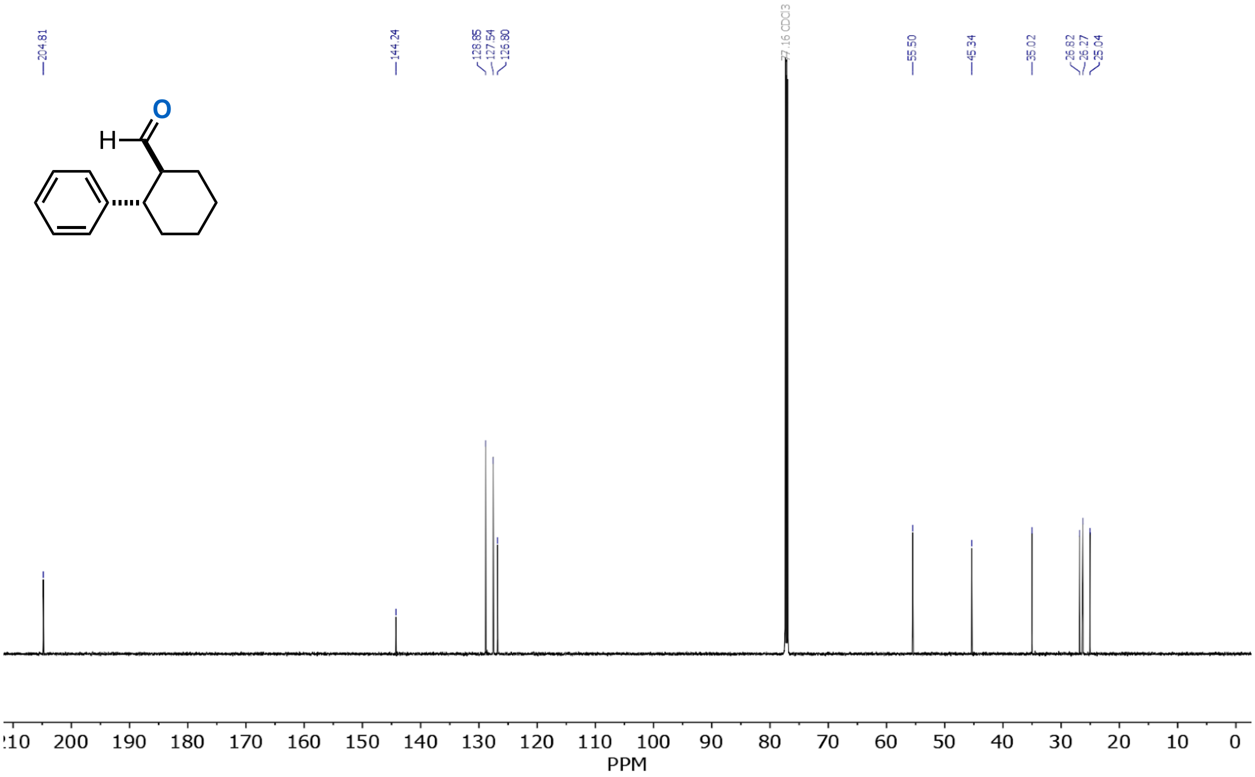

^{1}H NMR (400 MHz, $CDCl_3$) of spiro[benzo[d][1,3]dioxole-2,1'-cyclohexane] (2o)

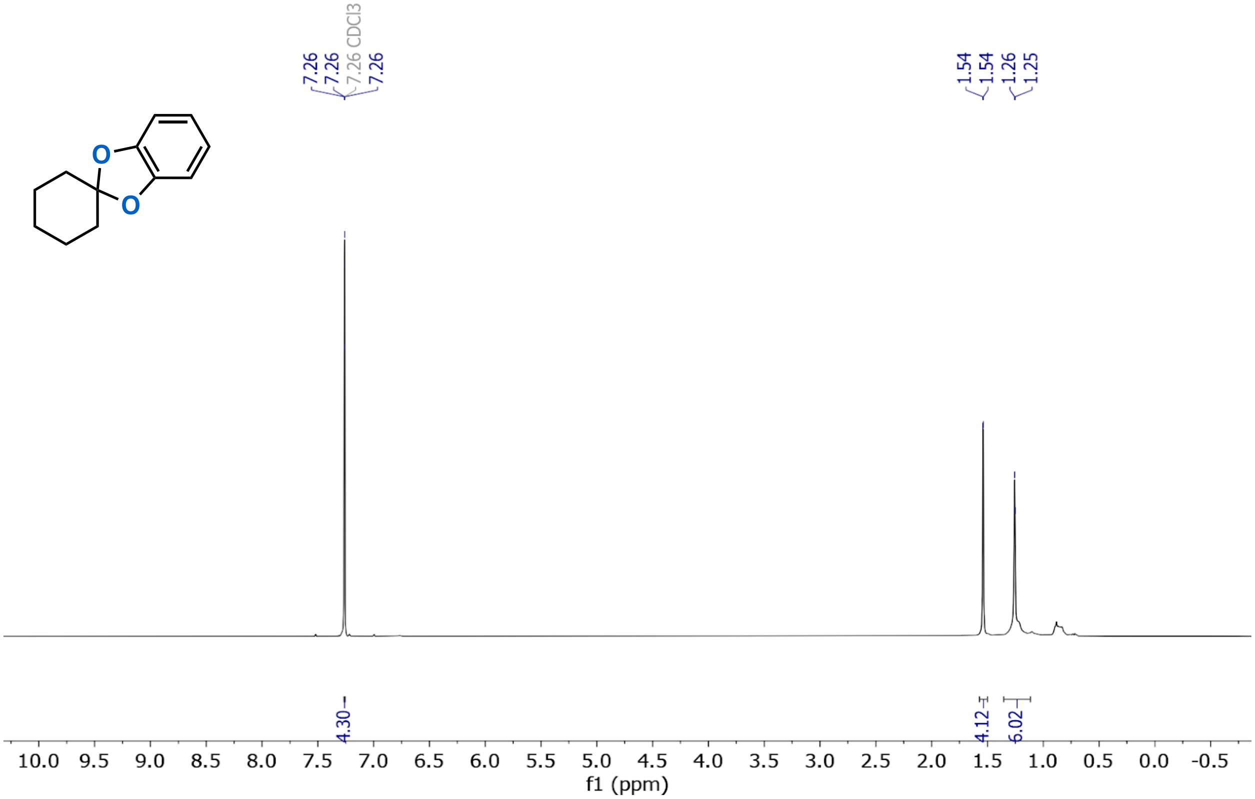

^{13}C{H} NMR (126 MHz, $CDCl_3$) of spiro[benzo[d][1,3]dioxole-2,1'-cyclohexane] (2o)

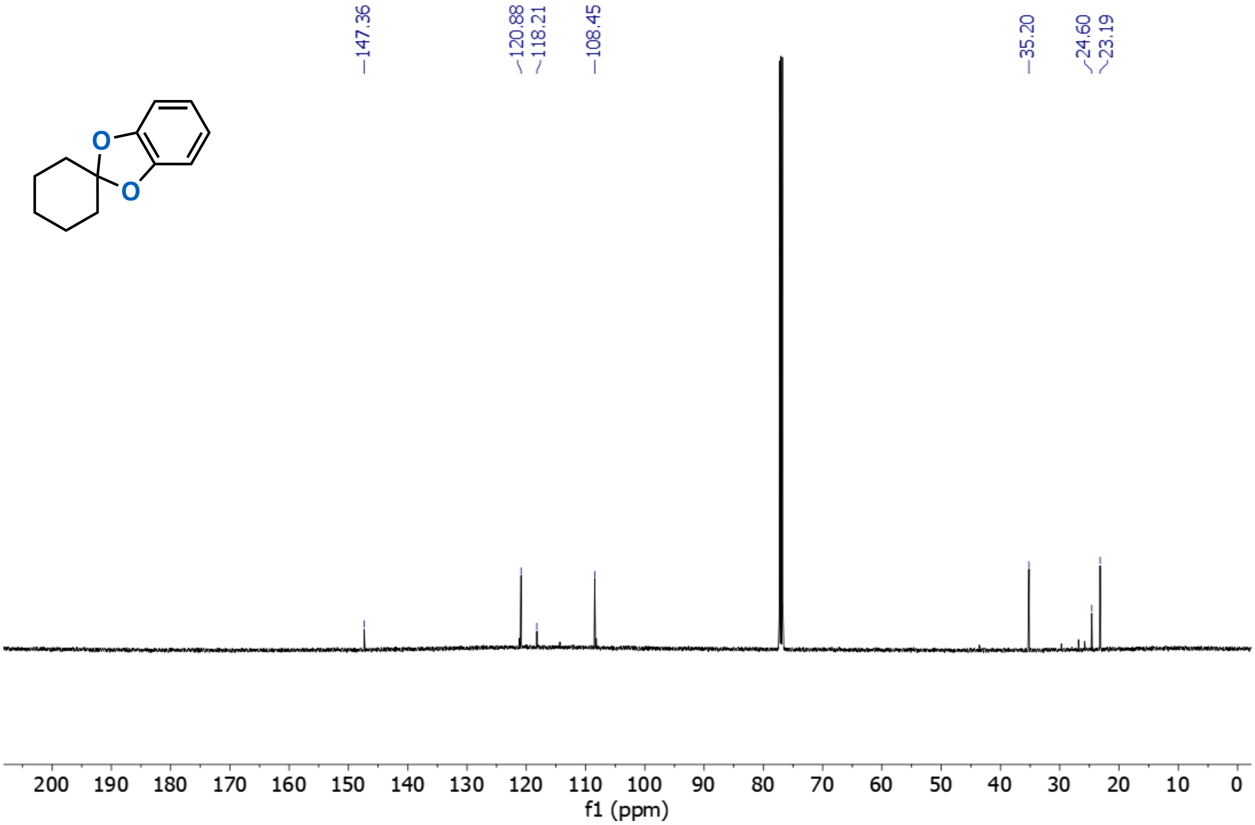

^{1}H NMR (500 MHz, $CDCl_3$) of 3-methyl-5-phenylpentan-2-one (2p)

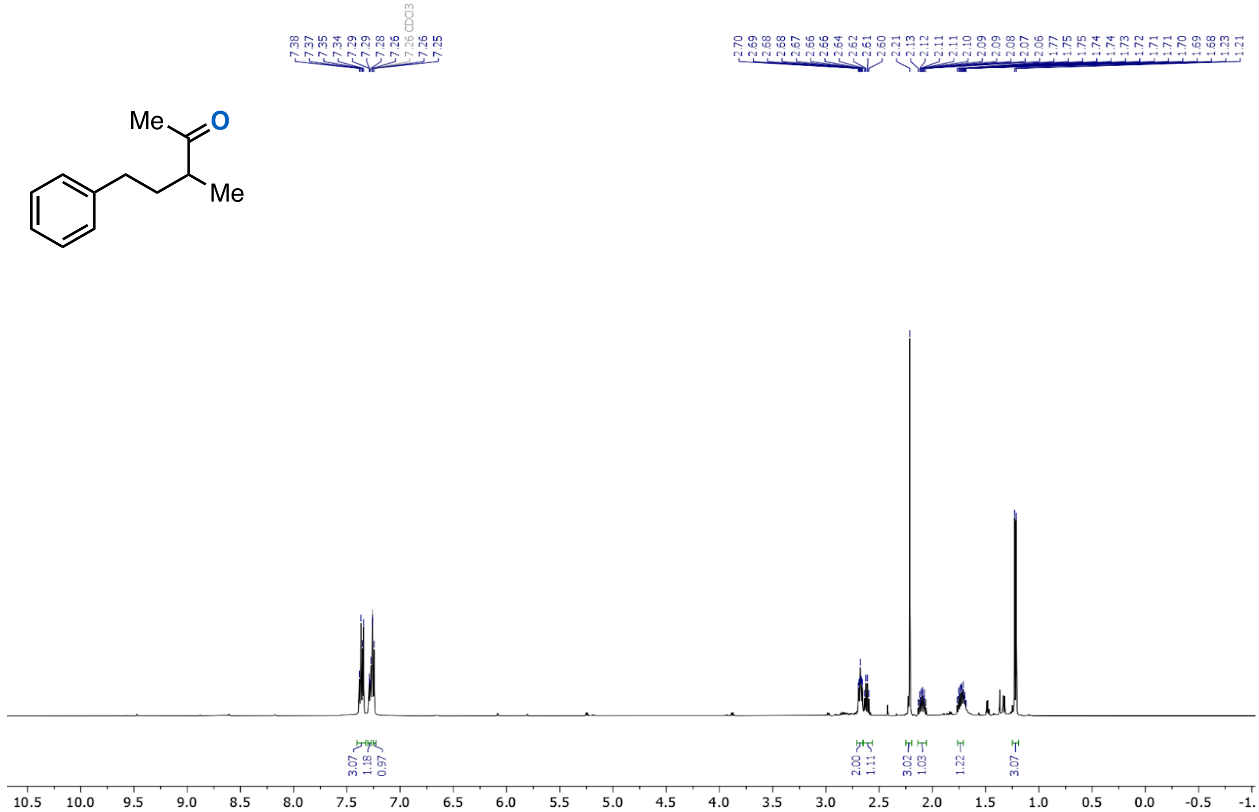

^{13}C{H} NMR (126 MHz, $CDCl_3$) of 3-methyl-5-phenylpentan-2-one (2p)

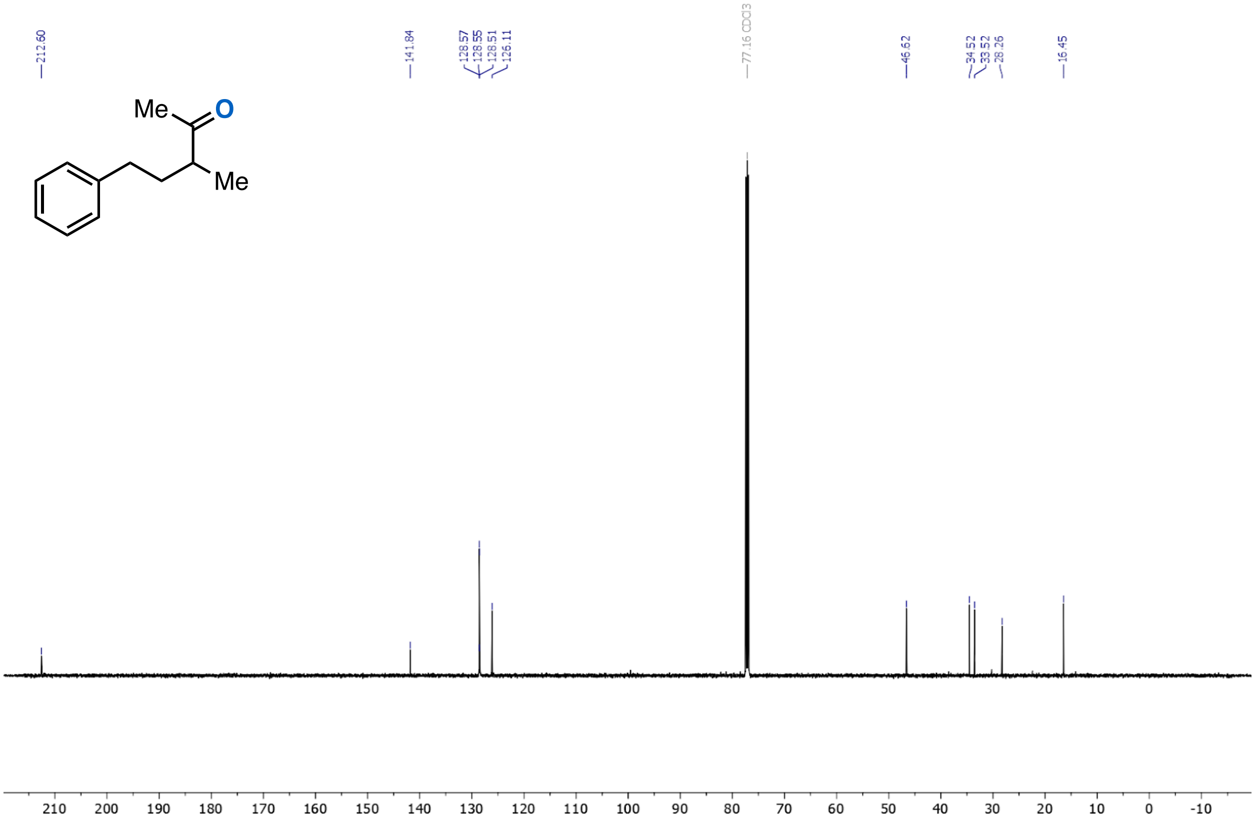

^{1}H NMR (500 MHz, $CDCl_3$) of (R)-3,7-dimethyl-6-oxooctyl benzoate (2q)

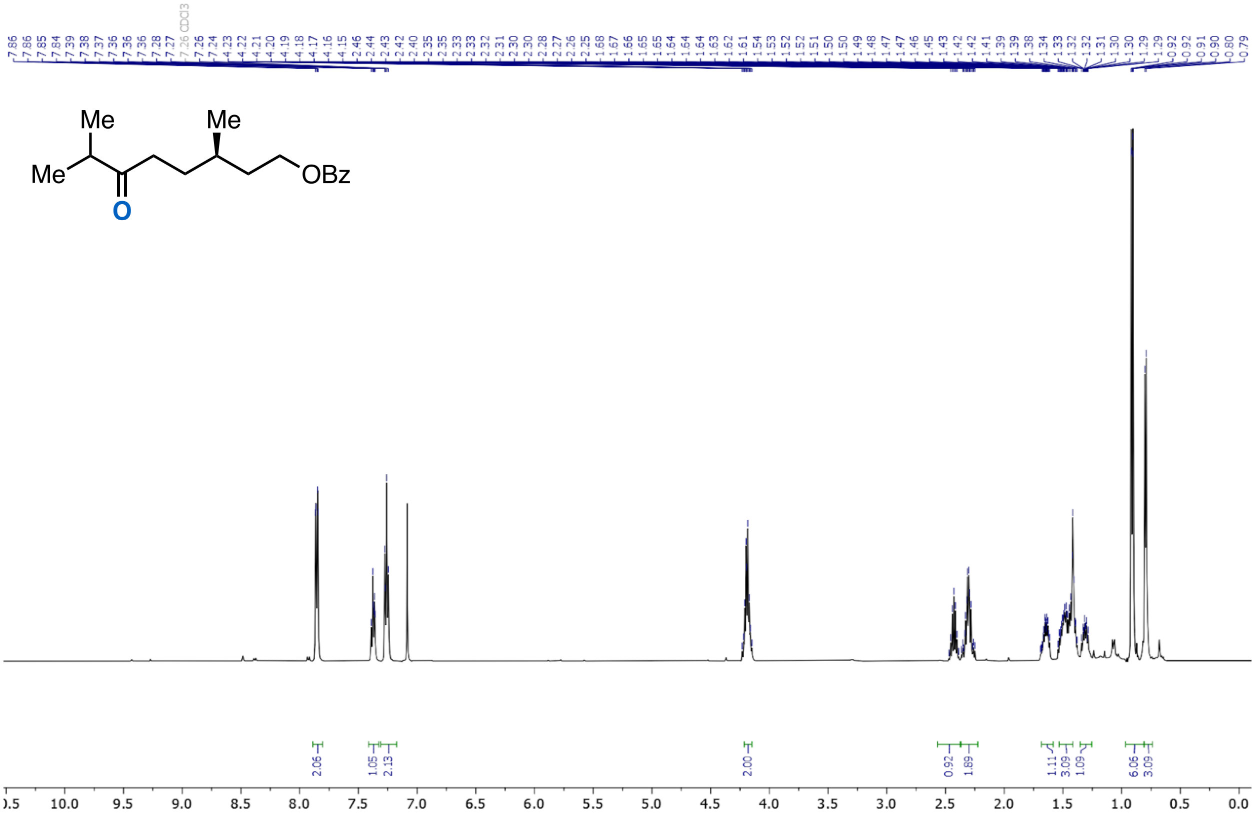

^{13}C{H} NMR (126 MHz, $CDCl_3$) of (R)-3,7-dimethyl-6-oxooctyl benzoate (2q)

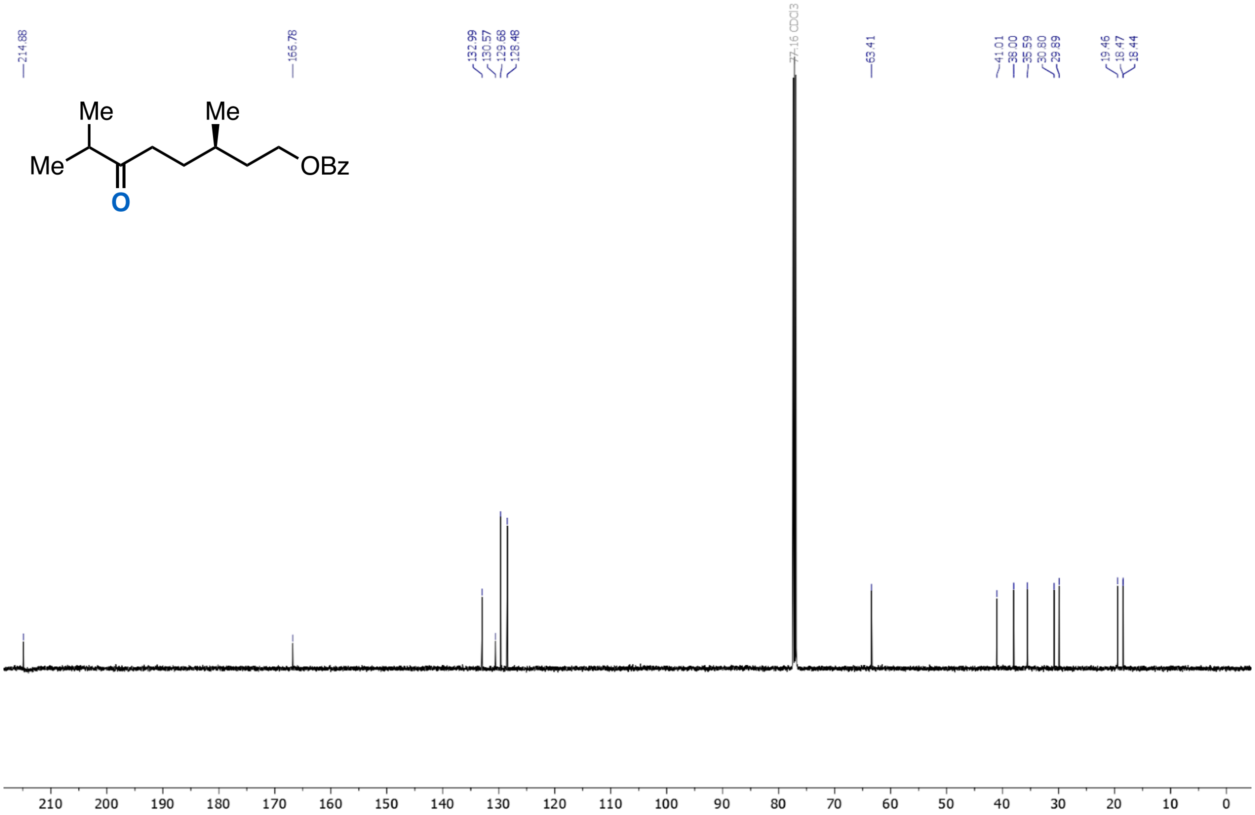

^{1}H NMR (400 MHz, $CDCl_3$) of (R)-2-(4-methyl-5-oxocyclohex-3-en-1-yl)propanal (2r)

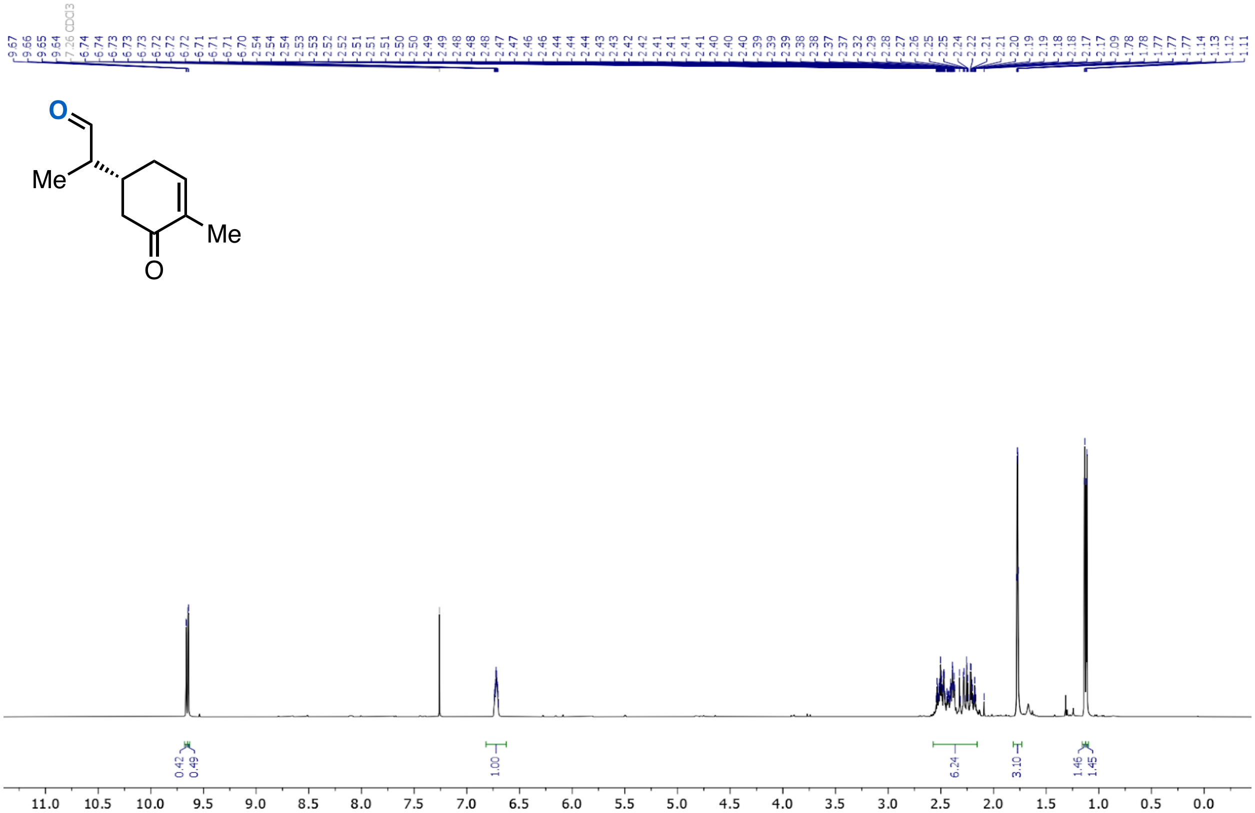

^{13}C{H} NMR (101 MHz, $CDCl_3$) of (R)-2-(4-methyl-5-oxocyclohex-3-en-1-yl)propanal (2r)

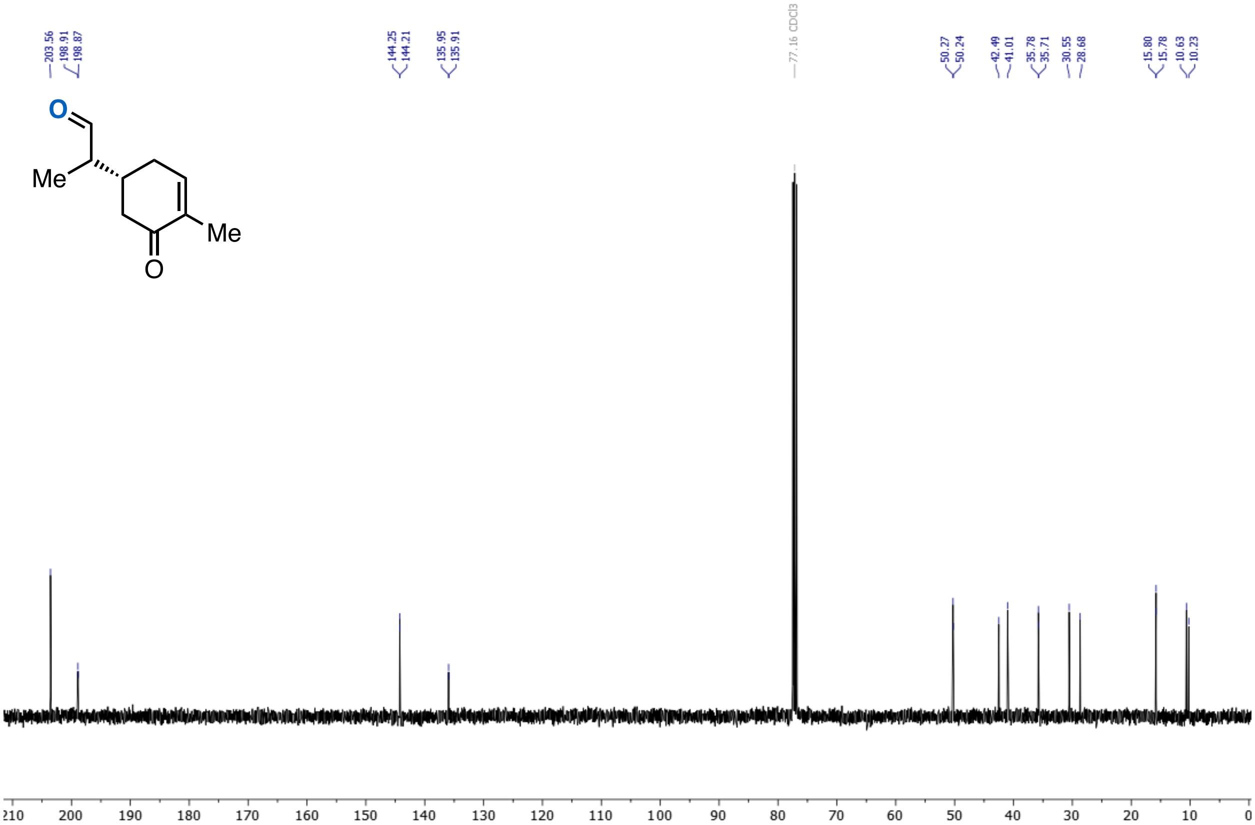

^{1}H NMR (400 MHz, CDCl$_3$) of (1*R*,8*S*)-4,10,10-trimethyl-7-methylenebicyclo[6.2.0]decane-4-carbaldehyde (2s)

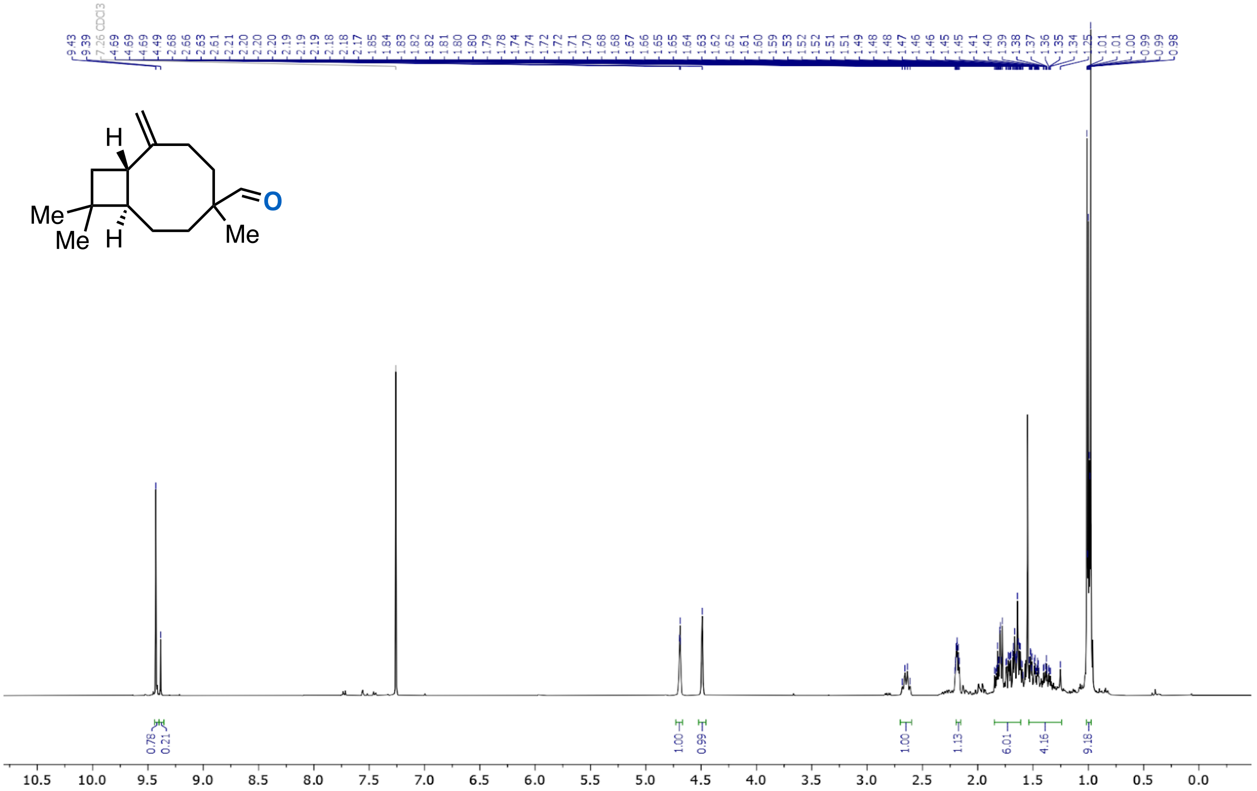

^{13}C{H} NMR (101 MHz, CDCl$_3$) of (1*R*,8*S*)-4,10,10-trimethyl-7-methylenebicyclo[6.2.0]decane-4-carbaldehyde (2s)

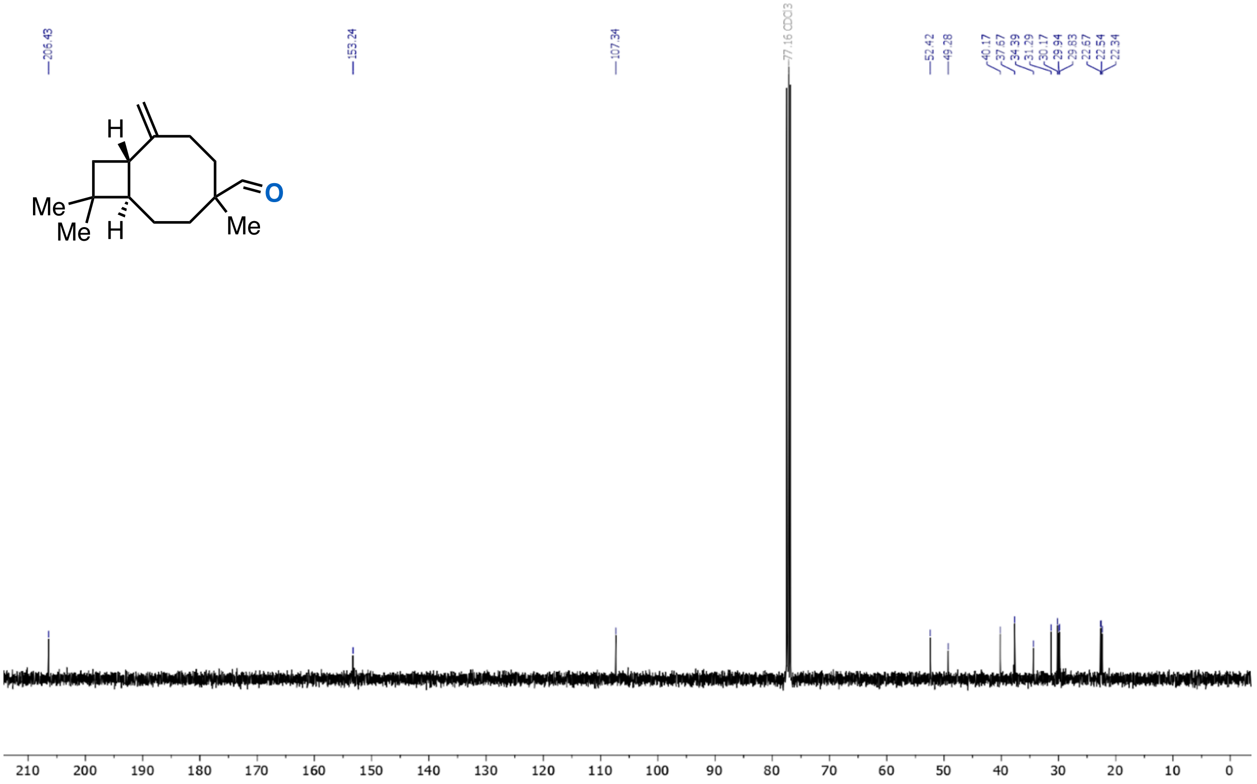

^{1}H NMR (500 MHz, $CDCl_3$) of ((1R,3aS,5aR,5bR,7aR,9S,11aR,11bR,13bR)-9-acetoxy-5a,5b,8,8,11a-pentamethyl-1-((S)-1-oxopropan-2-yl)icosahydro-3aH-cyclopenta[a]chrysen-3a-yl)methyl acetate (2t)

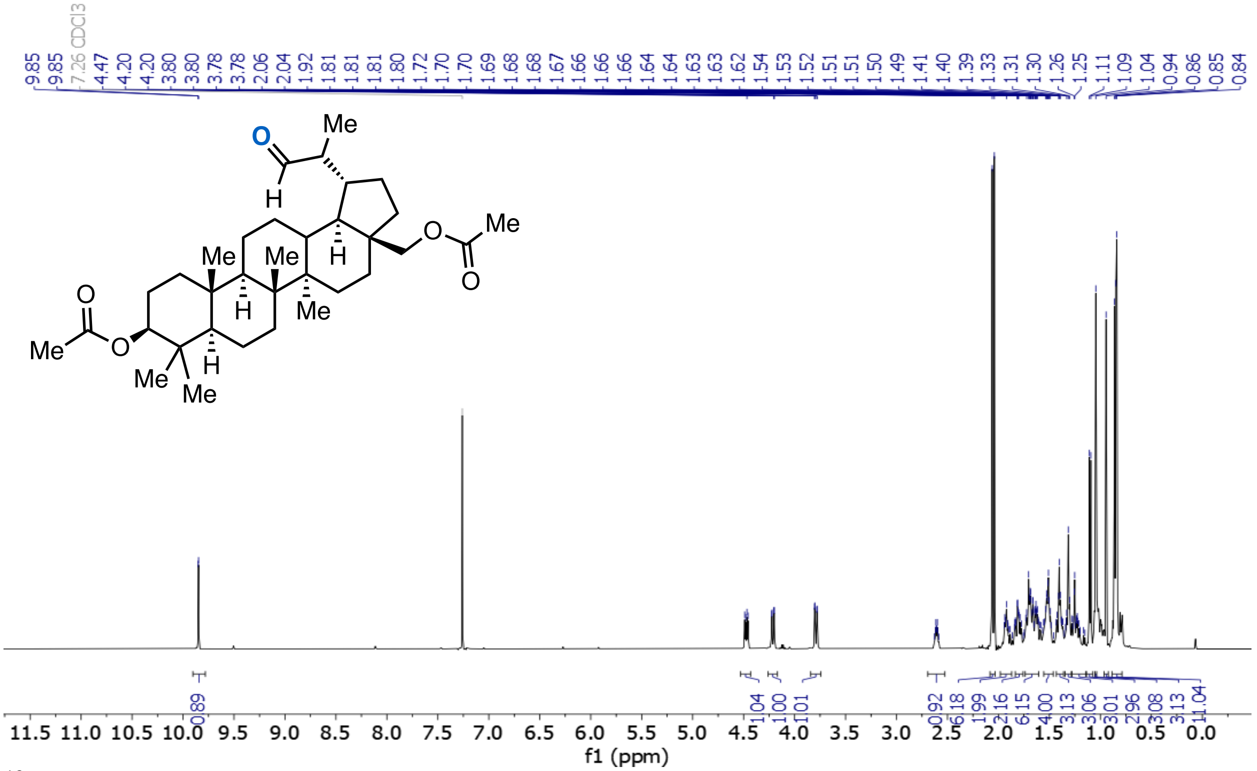

^{13}C{H} NMR (126 MHz, $CDCl_3$) of ((1R,3aS,5aR,5bR,7aR,9S,11aR,11bR,13bR)-9-acetoxy-5a,5b,8,8,11a-pentamethyl-1-((S)-1-oxopropan-2-yl)icosahydro-3aH-cyclopenta[a]chrysen-3a-yl)methyl acetate (2t)

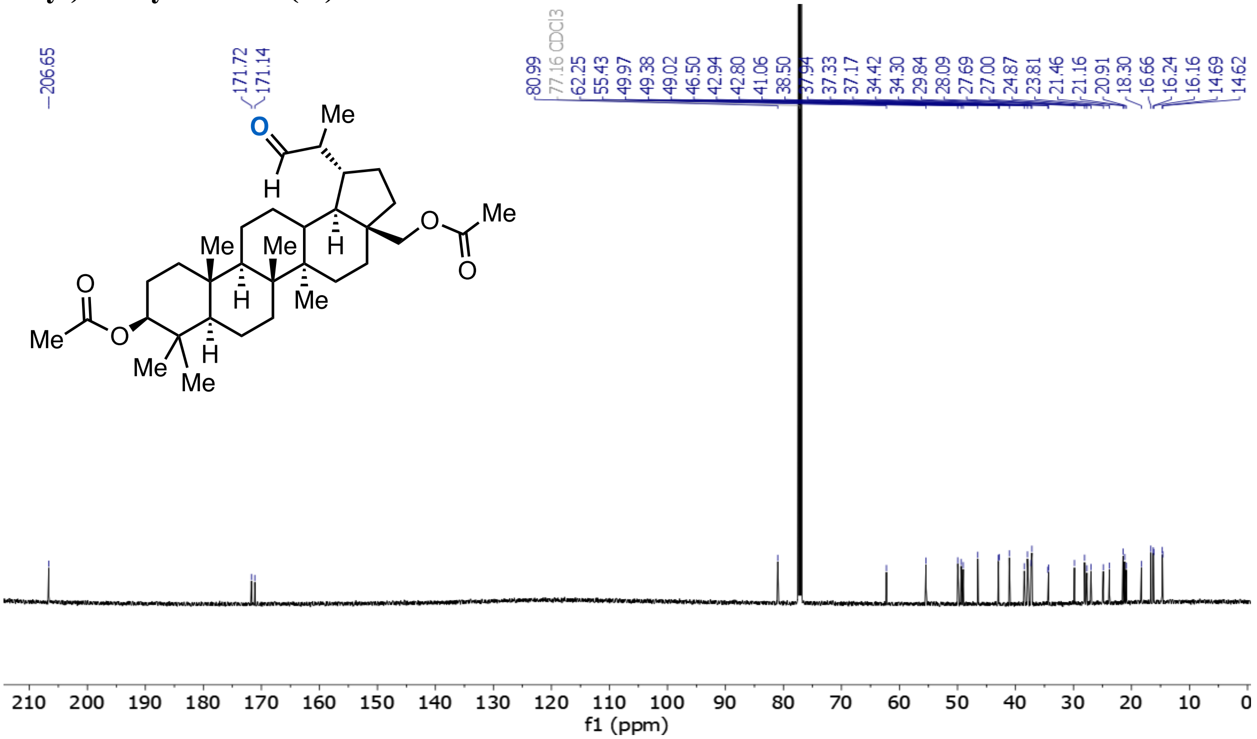

^{1}H NMR (400 MHz, $CDCl_3$) of 2,2-dimethyl-4-phenethyl-1,3-dioxolane (11a)

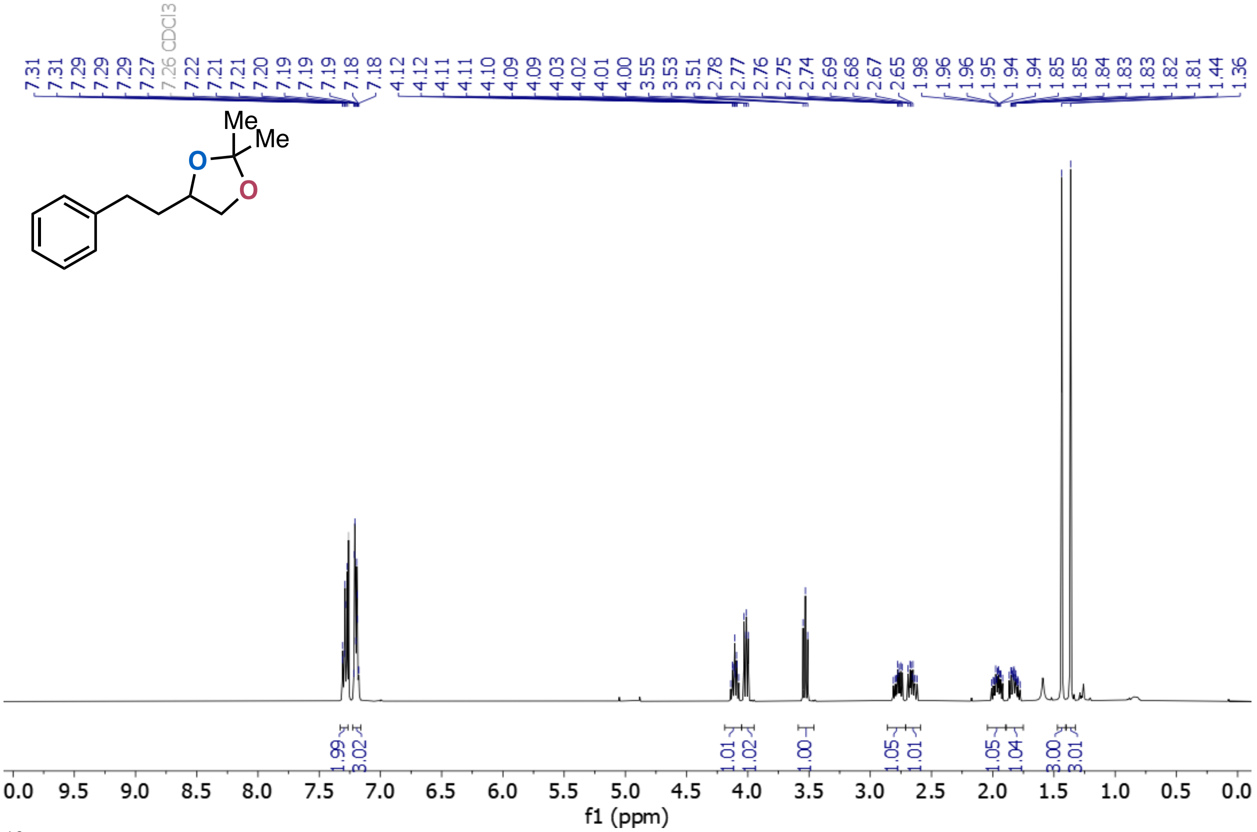

^{13}C{H} NMR (101 MHz, $CDCl_3$) of 2,2-dimethyl-4-phenethyl-1,3-dioxolane (11a)

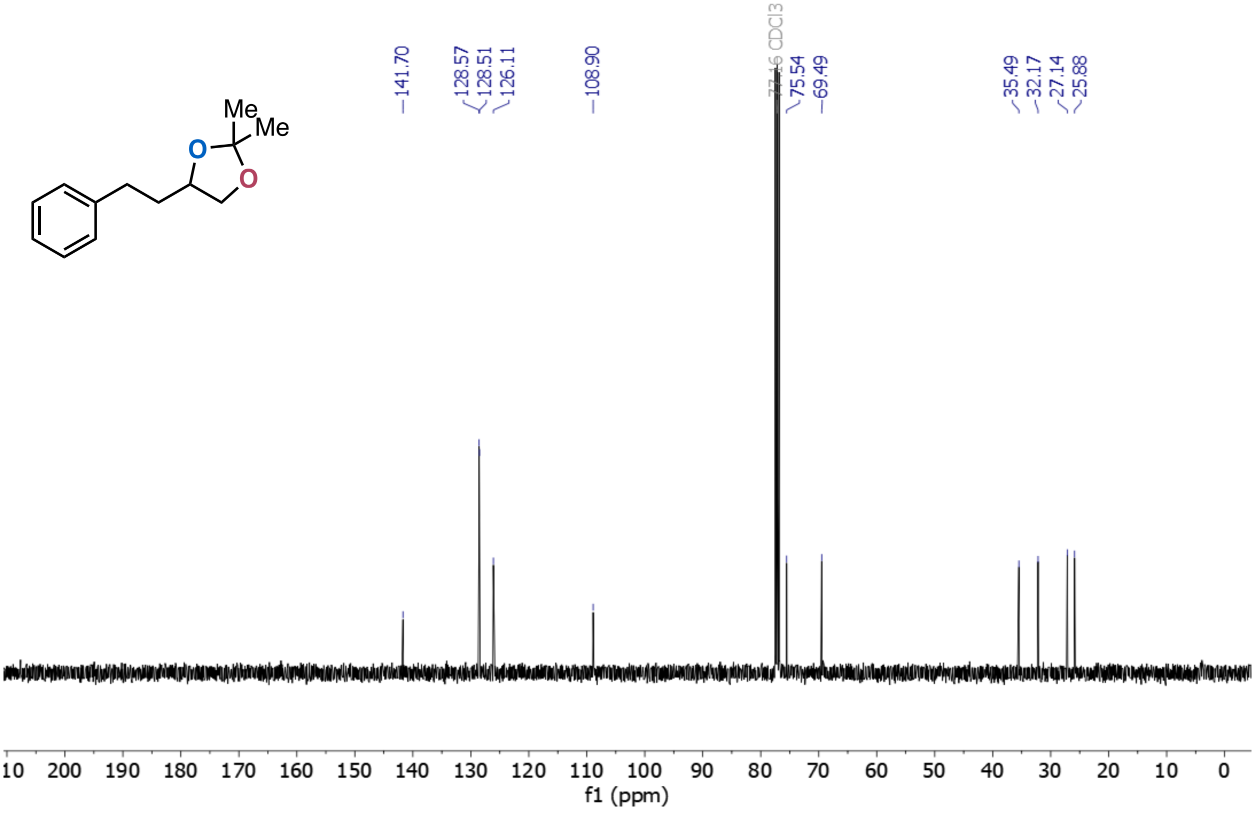

^{1}H NMR (500 MHz, CDCl₃) of 4-decyl-2,2-dimethyl-1,3-dioxolane (11b)

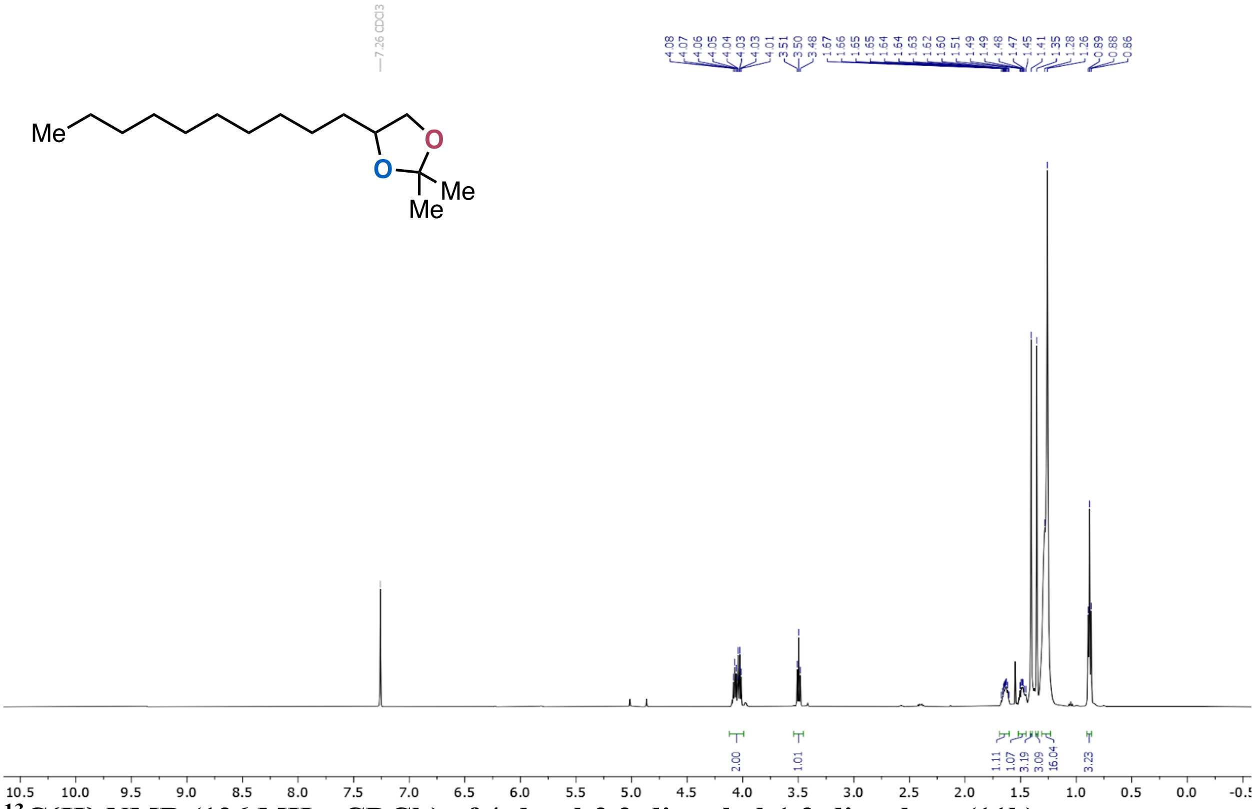

^{13}C{H} NMR (126 MHz, CDCl₃) of 4-decyl-2,2-dimethyl-1,3-dioxolane (11b)

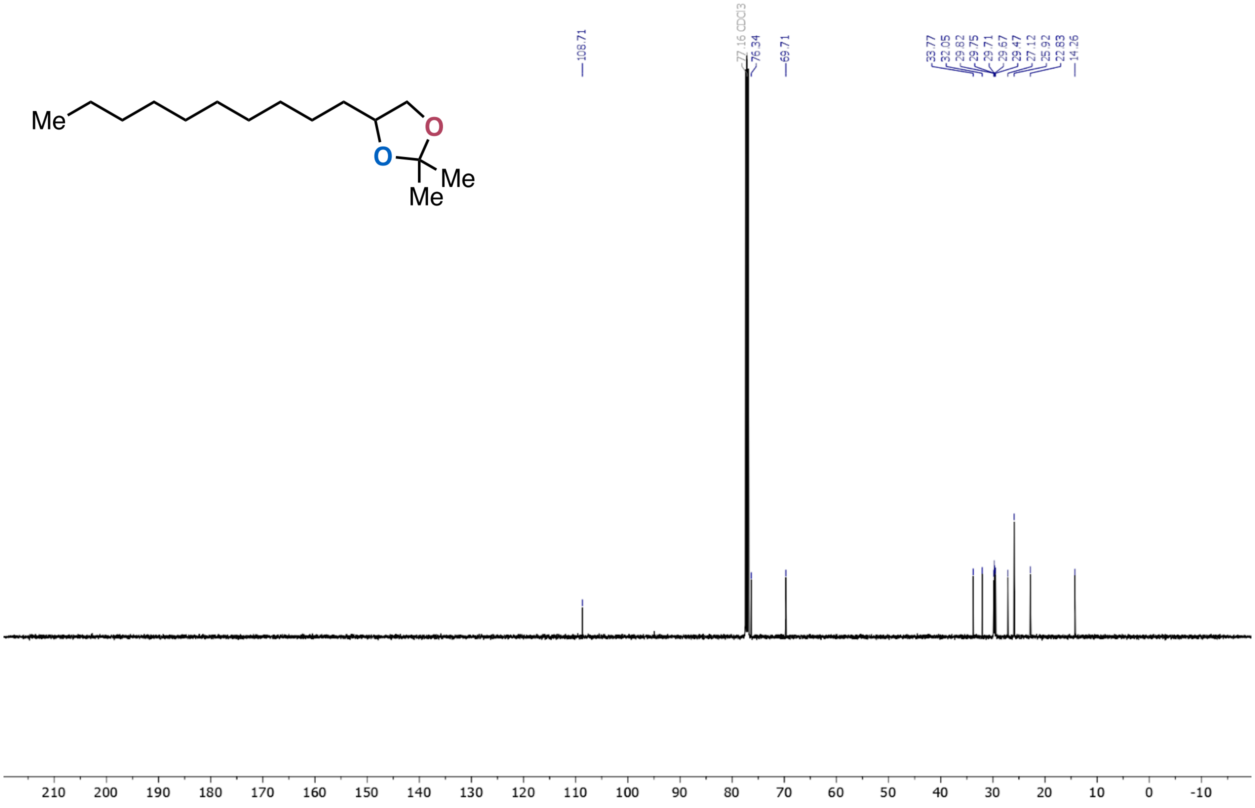

^{1}H NMR (500 MHz, $CDCl_3$) of 6-(2,2-dimethyl-1,3-dioxolan-4-yl)hexyl acetate (11c)

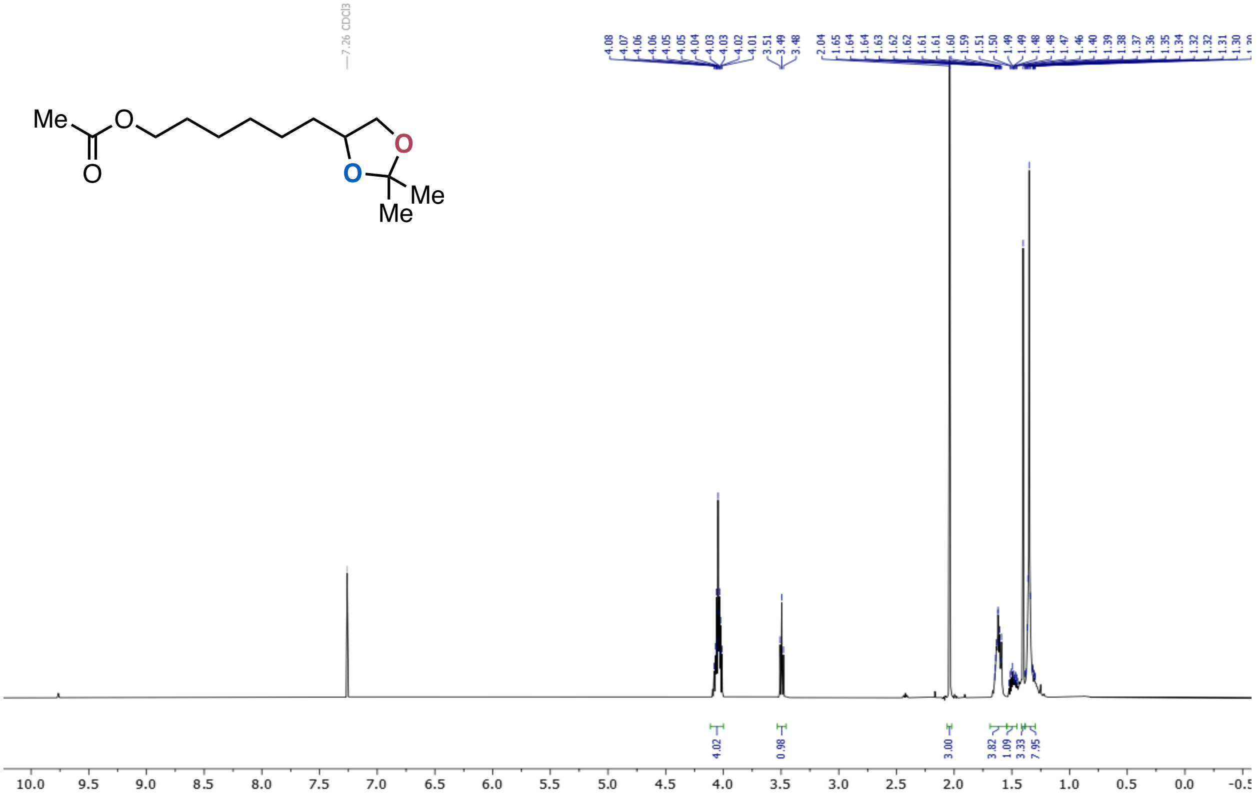

^{13}C{H} NMR (126 MHz, $CDCl_3$) of 6-(2,2-dimethyl-1,3-dioxolan-4-yl)hexyl acetate (11c)

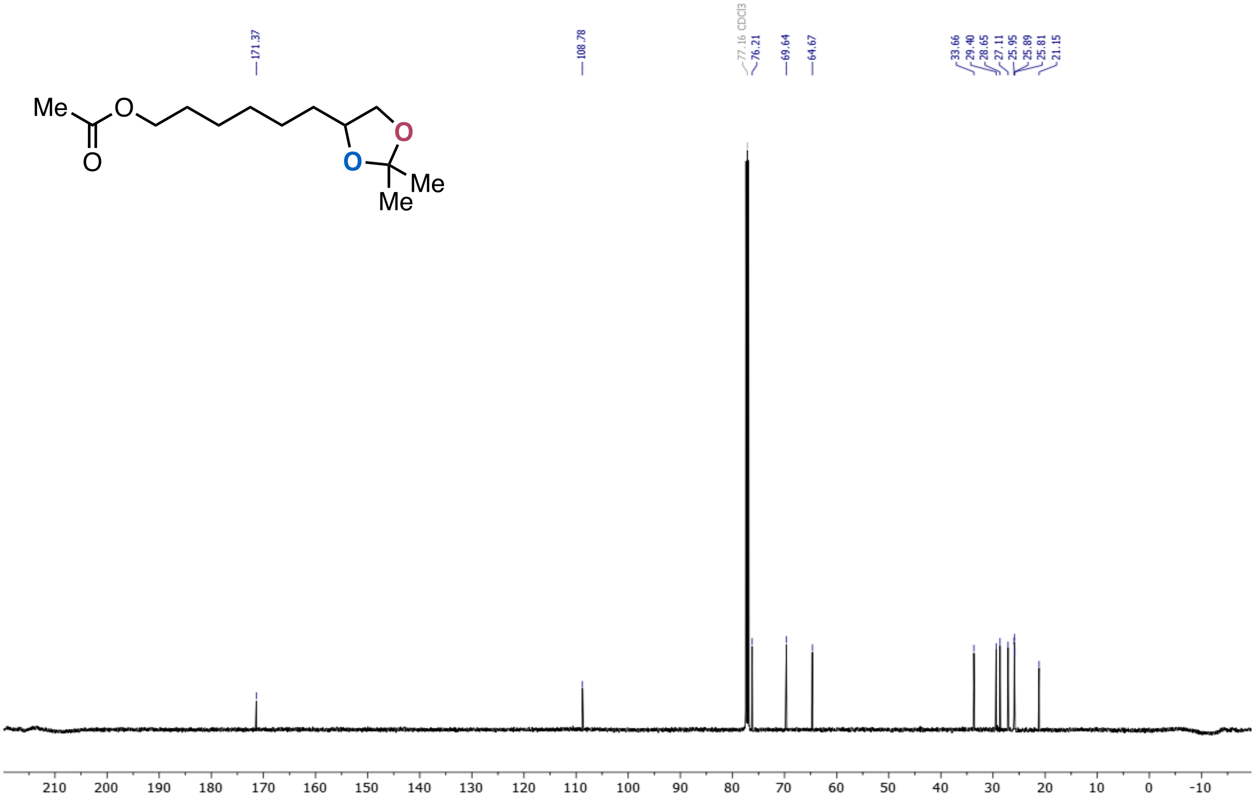

^{1}H NMR (500 MHz, $CDCl_3$) of 8-bromooctane-1,2-diol (11d)

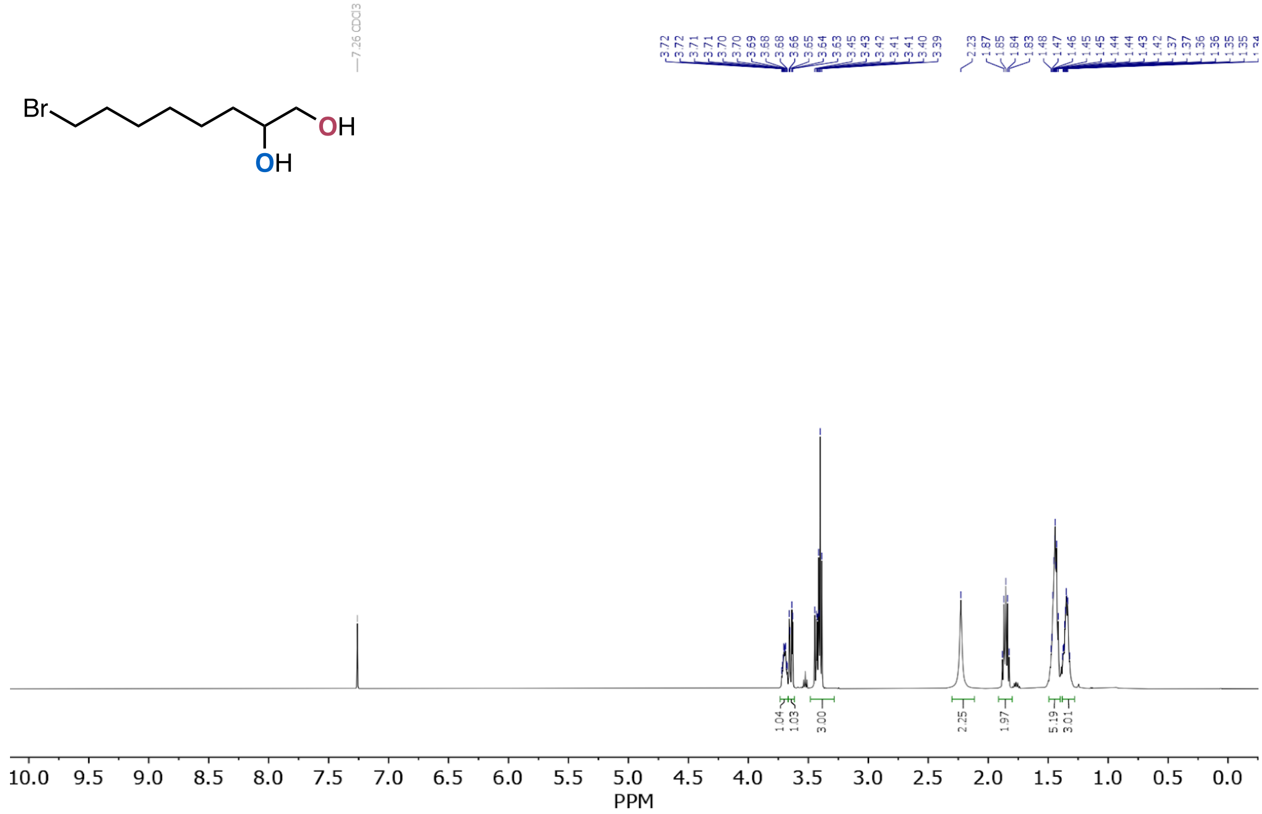

^{13}C{H} NMR (126 MHz, $CDCl_3$) of 8-bromooctane-1,2-diol (11d)

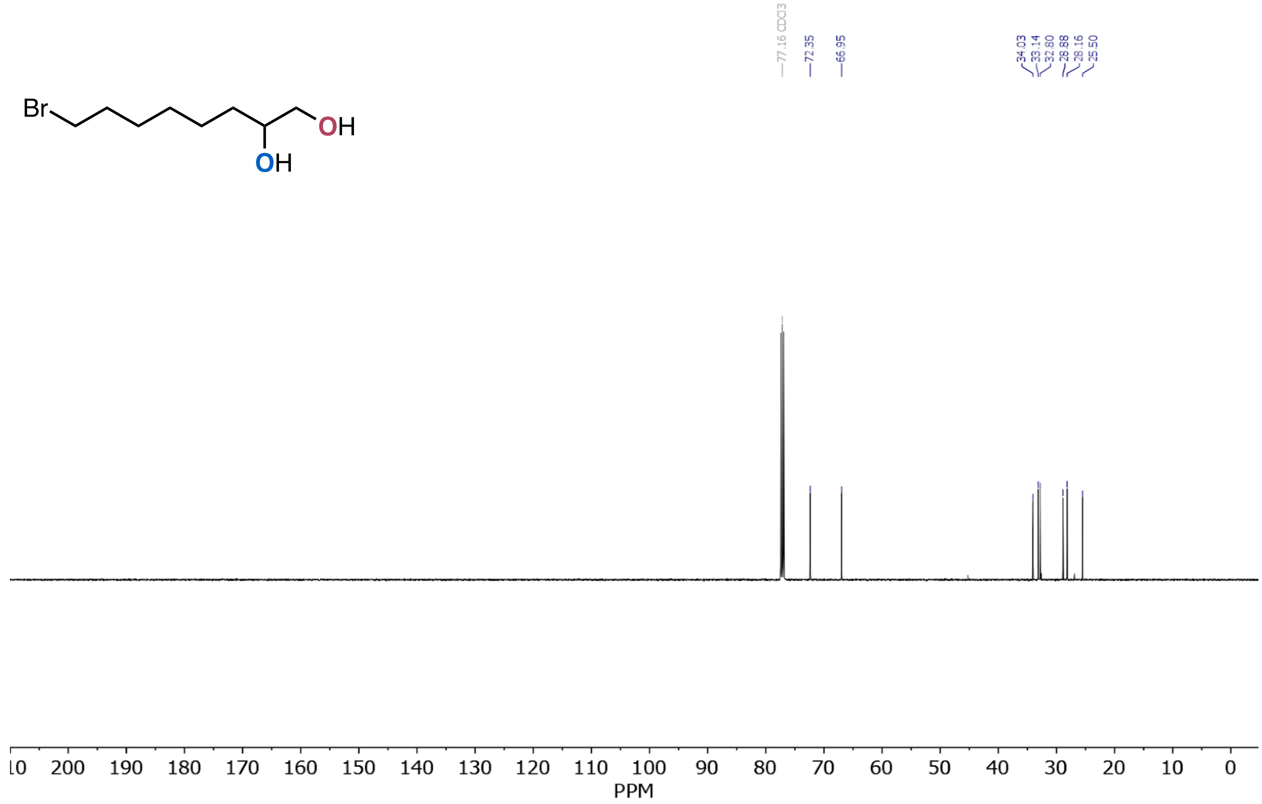

^{1}H NMR (400 MHz, $CDCl_3$) of 2-(6-(2,2-dimethyl-1,3-dioxolan-4-yl)hexyl)isoindoline-1,3-dione (11e)

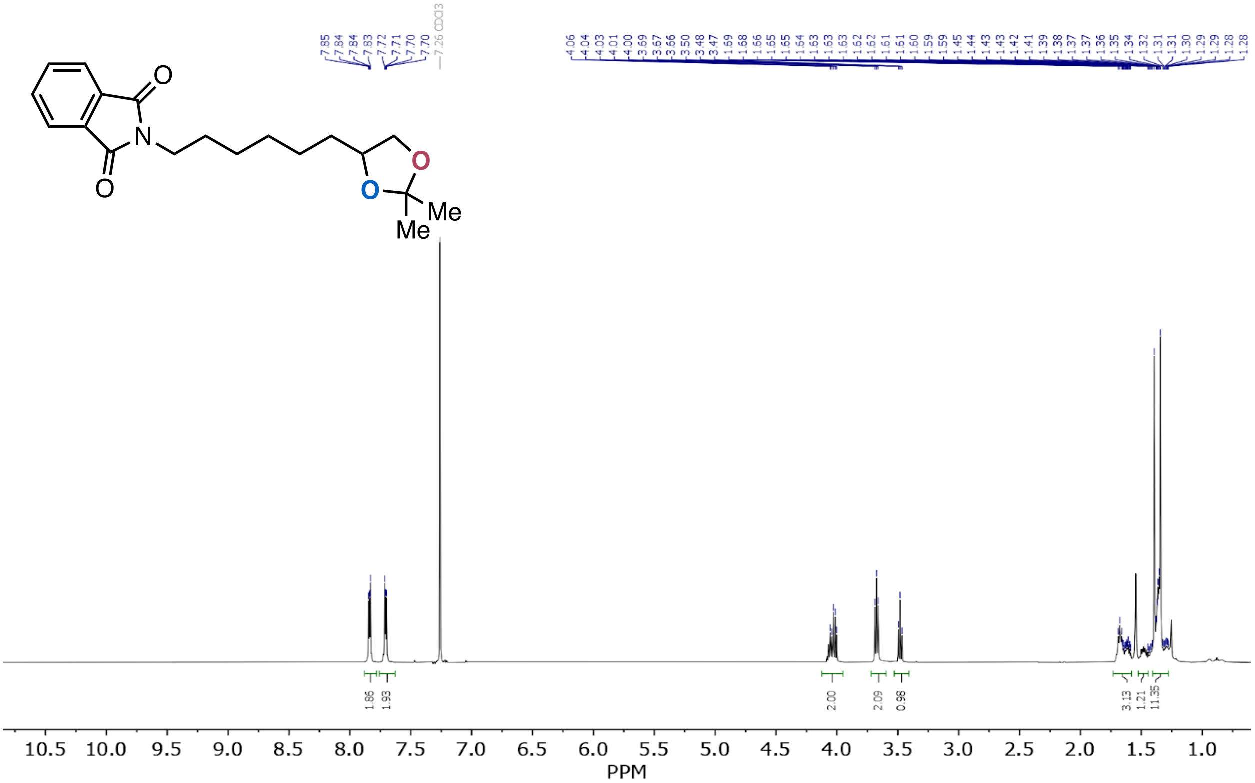

^{13}C{H} NMR (101 MHz, $CDCl_3$) of 2-(6-(2,2-dimethyl-1,3-dioxolan-4-yl)hexyl)isoindoline-1,3-dione (11e)

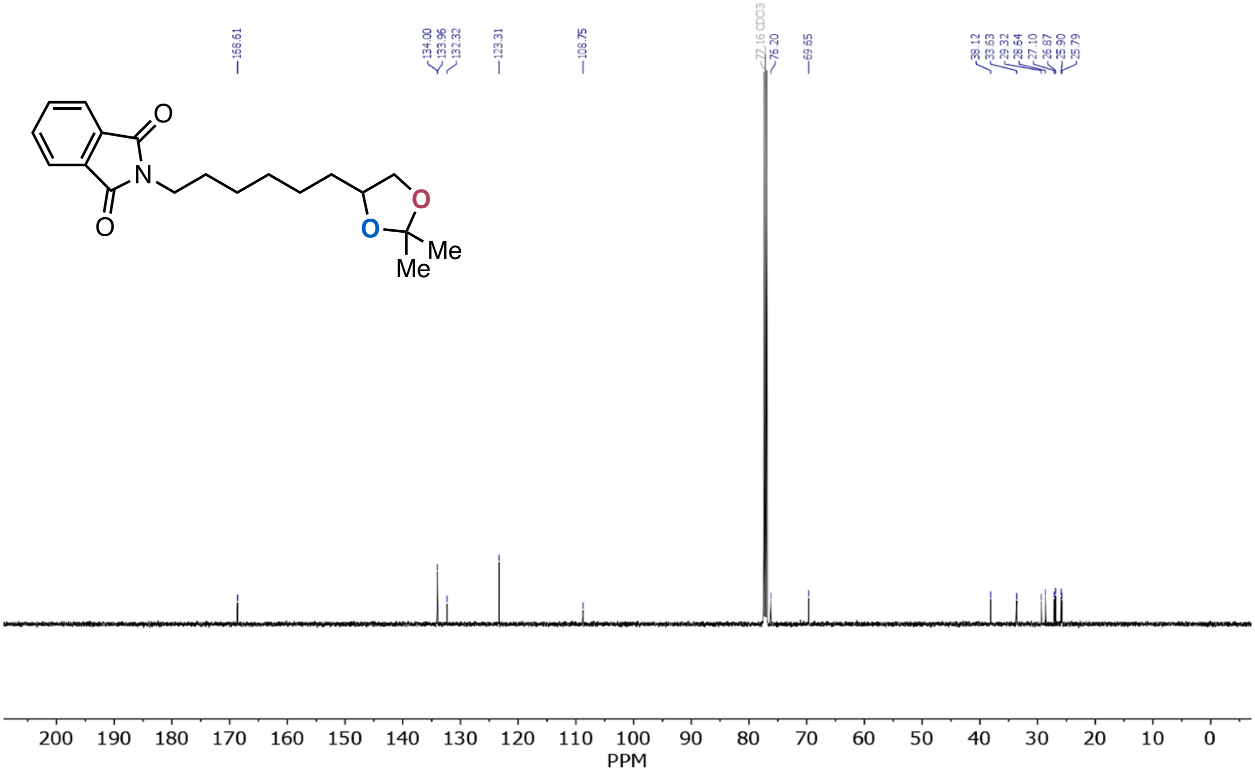

^{1}H NMR (500 MHz, CDCl$_3$) of *tert*-butyl (6-(2,2-dimethyl-1,3-dioxolan-4-yl)hexyl)carbamate (11f)

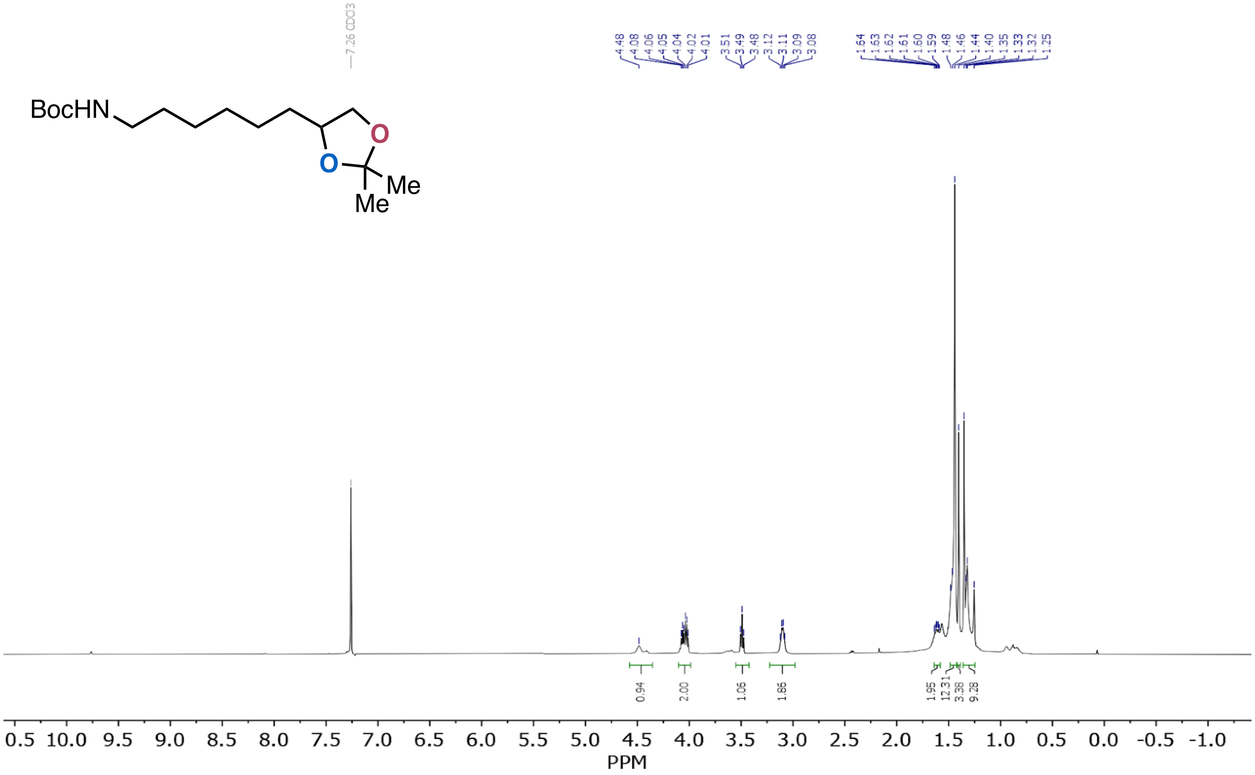

^{13}C{H} NMR (126 MHz, CDCl$_3$) of *tert*-butyl (6-(2,2-dimethyl-1,3-dioxolan-4-yl)hexyl)carbamate (11f)

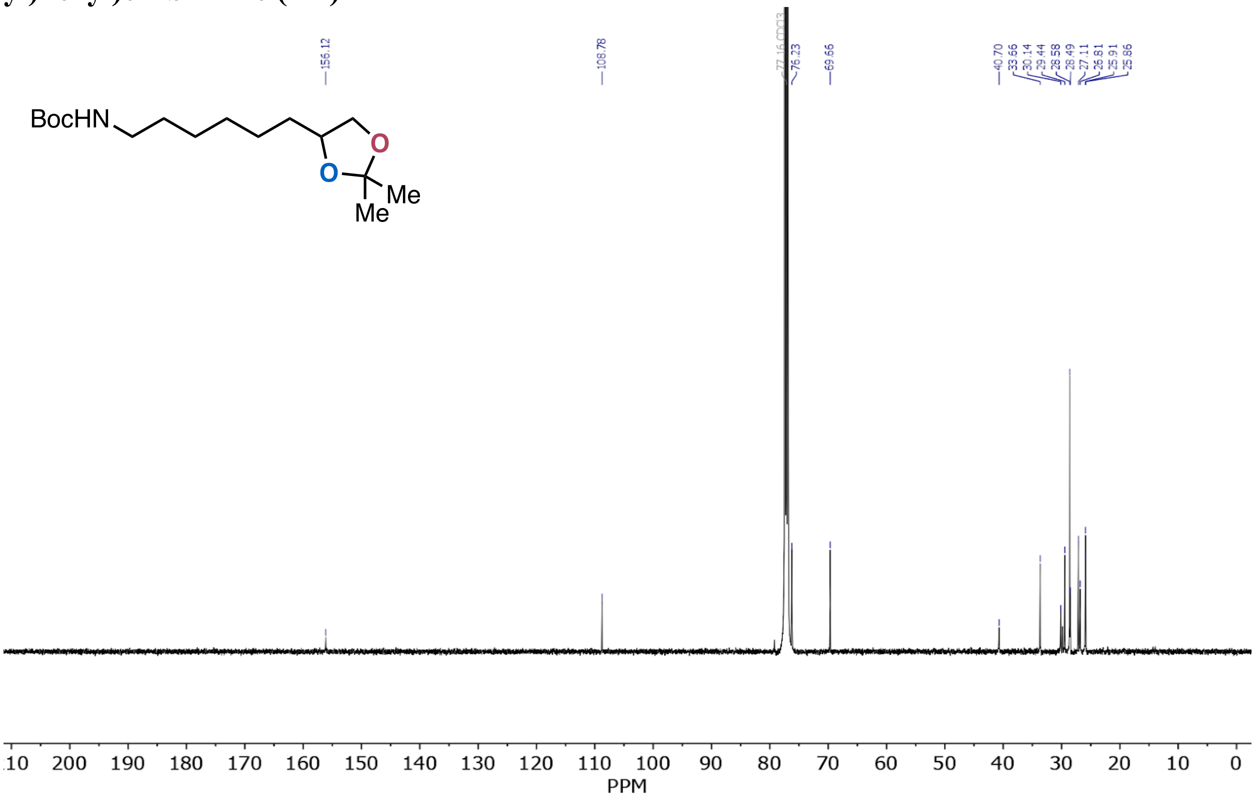

^{1}H NMR (400 MHz, $CDCl_3$) of 4,5-dibutyl-2,2-dimethyl-1,3-dioxolane (11g)

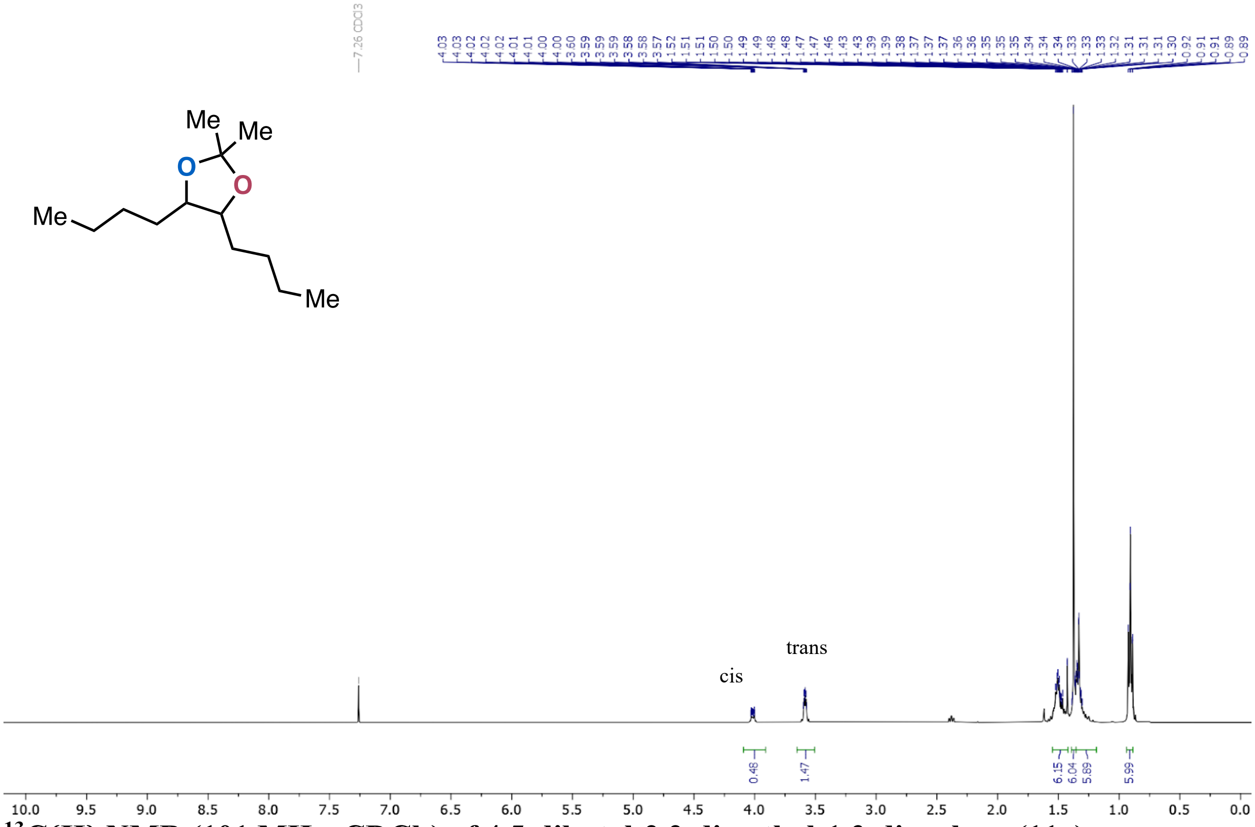

^{13}C{H} NMR (101 MHz, $CDCl_3$) of 4,5-dibutyl-2,2-dimethyl-1,3-dioxolane (11g)

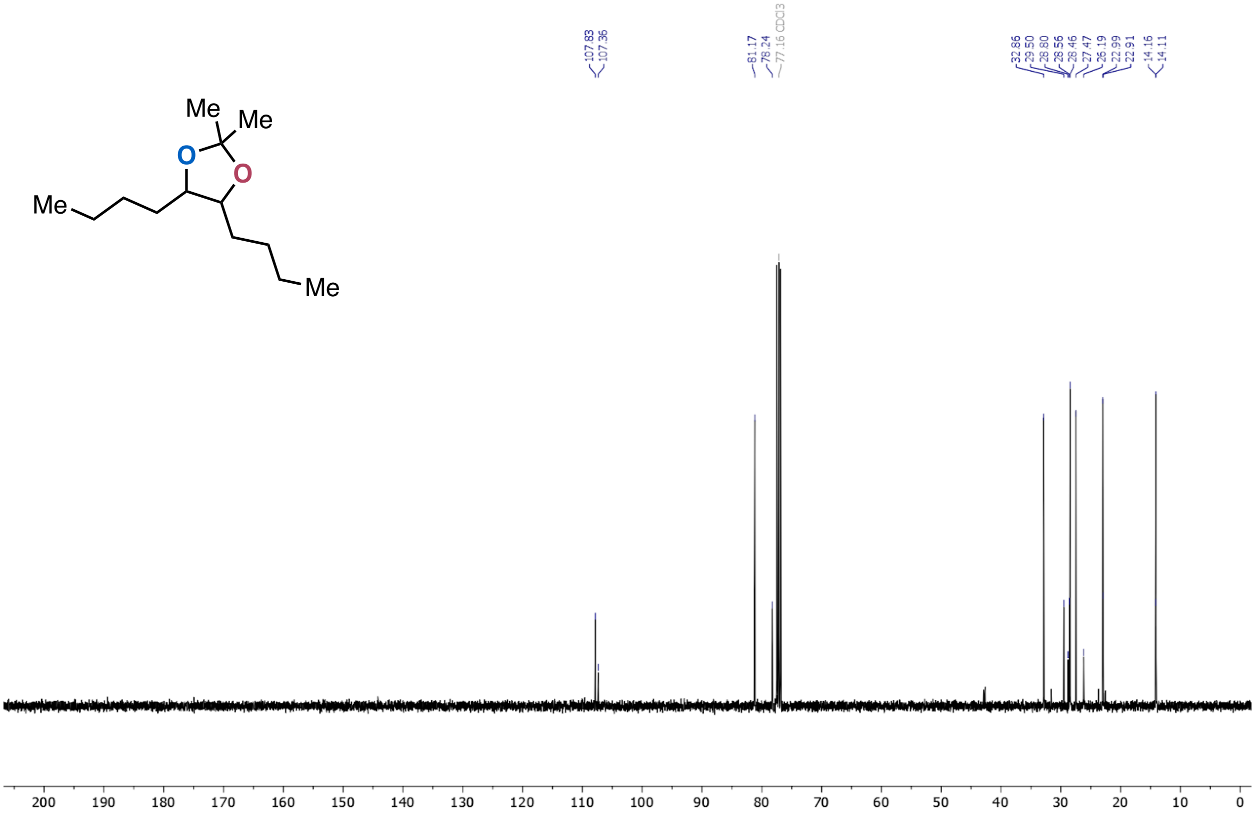

^{1}H NMR (400 MHz, $CDCl_3$) of methyl 8-(2,2-dimethyl-5-octyl-1,3-dioxolan-4-yl)octanoate (11h)

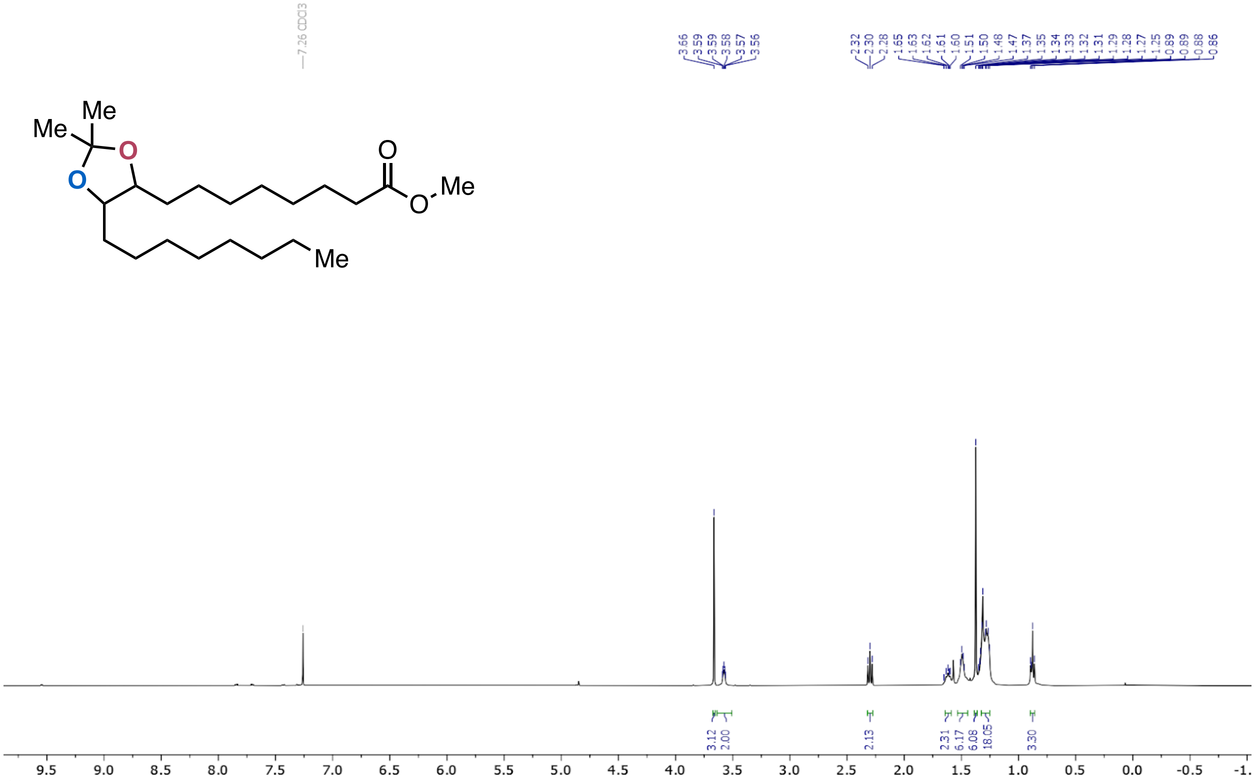

^{13}C{H} NMR (101 MHz, $CDCl_3$) of methyl 8-(2,2-dimethyl-5-octyl-1,3-dioxolan-4-yl)octanoate (11h)

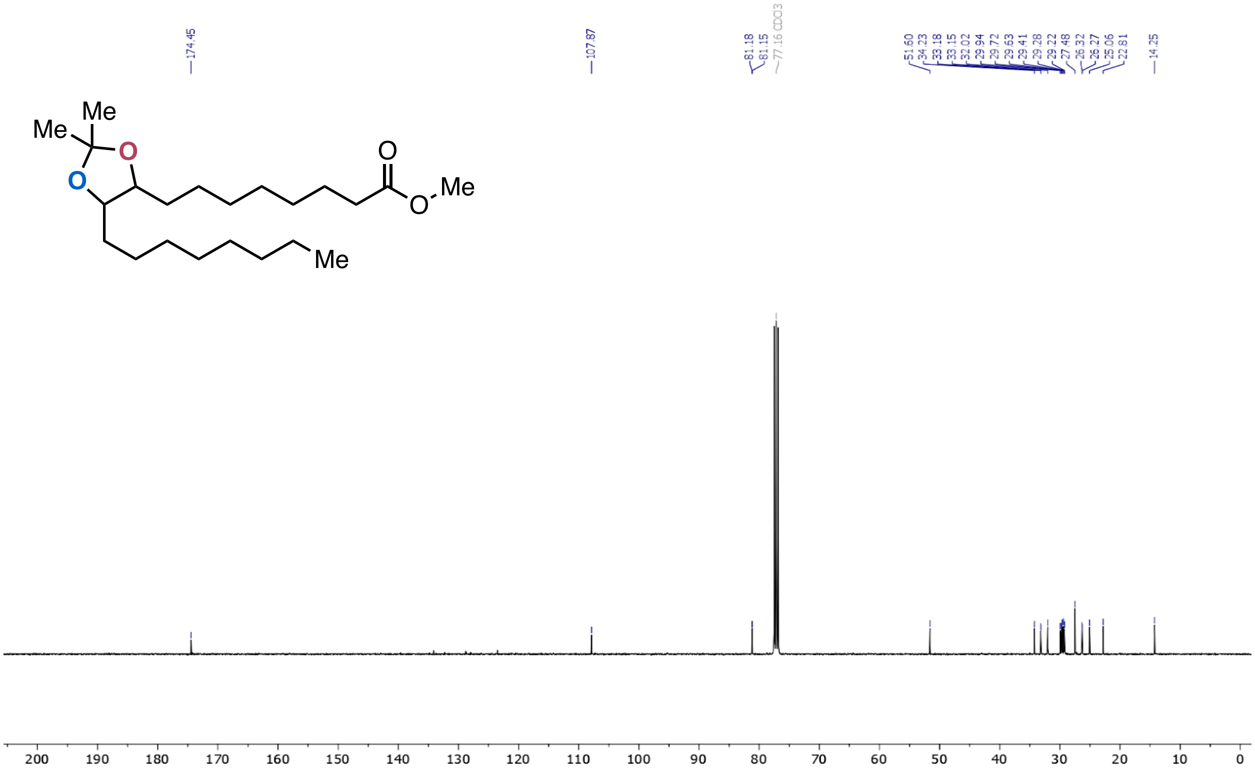

References

1. X. Bertrand, J.-F. Paquin, *Org. Lett.* **2019**, *21*, 9759–9762.
2. A. Ruffoni, C. Hampton, M. Simonetti, D. Leonori, *Nature* **2022**, *610*, 81–86.
3. M. F. Buffet, D. J. Dixon, G. L. Edwards, S. V. Ley, E. W. Tate, *J. Chem. Soc., Perkin Trans. 1* **2000**, 1815–1827.
4. S. J. Leiris, O. M. Khdour, Z. J. Segerman, K. S. Tsosie, J.-C. Chapuis, S. M. Hecht, *Bioorg. Med. Chem.* **2010**, *18*, 3481–3493.
5. R.-Z. Huang, K. K. Lau, Z. Li, T.-L. Liu, Y. Zhao, *J. Am. Chem. Soc.* **2018**, *140*, 14647–14654.
6. S. W. Youn, S. J. Pastine, D. Sames, *Org. Lett.* **2004**, *6*, 581–584.
7. R. C. Santos, J. A. R. Salvador, S. Marín, M. Cascante, J. N. Moreira, T. C. P. Dinis, *Bioorg. Med. Chem.* **2010**, *18*, 4385–4396.
8. F. Romanov-Michailidis, K. F. Sedillo, J. M. Neely, T. Rovis, *J. Am. Chem. Soc.* **2015**, *137*, 8892–8895.
9. Komori, S.; Yamaguchi, Y.; Kataoka, Y.; Ura, Y., *J. Org. Chem.* **2019**, *84*, 3093–3099.
10. A. R. Katritzky, Z. Yang, D. J. Cundy, *Synth. Commun.* **1993**, *23*, 3061–3071.
11. Coxon, J. M.; Hartshorn, M. P.; Swallow, W. H., *J. Org. Chem.* **1974,** *39*, 1142–1148.
12. S. St John-Campbell, A. J. P. White, J. A. Bull, *Org. Lett.* **2020,** *22*, 1807–1812.
13.Harvison, P. J.; Forte, A. J.; Nelson, S. D., *J. Med. Chem.* **1986**, *29*, 1737–1743.
14. Stephen D. Holmbo, Nicole A. Godfrey, Joshua J. Hirner, and Sergey V. Pronin *J. Am. Chem. Soc.* **2016**, *138*, 12316–12319.
15. Torosyan, S. A.; Gimalova, F. A.; Valeev, R. F.; Miftakhov, M. S., *Russ. J. Org. Chem.* **2011**, *47*, 682–686.
16. Tatarova, L. E.; Korchagina, D. V.; Barkhash, V. A., *Russ. J. Org. Chem.* **2002,** *38*, 519–524.
17. Okamoto, I.; Takeya, T.; Kagawa, Y.; Kotani, E., *Chem. Pharm. Bull.* **2000**, *48*, 120–125.
18. Narasimhulu, M.; Srikanth Reddy, T.; Chinni Mahesh, K.; Sai Krishna, A.; Venkateswara Rao, J.; Venkateswarlu, Y., *Bioorg. Med. Chem. Lett.* **2009**, *19*, 3125–3127.
19. J. R. Vyvyan, J. A. Meyer, K. D. Meyer, *J. Org. Chem.* **2003**, *68*, 9144–9147.
20. Hampton, C.; Simonetti, M.; Leonori, D *Angew. Chem. Int. Ed.* **2023,** *62,* e202214508.
21. Curci, R.; D'Accolti, L.; Dinoi, A.; Fusco, C.; Rosa, A., *Tetrahedron Lett.* **1996,** *37*, 115–118.
22. T. Wistuba, C. Limberg, P. Kircher, *Angew. Chem. Int. Ed.* **1999**, *38*, 3037–3039.
23. M. Hatano, T. Nishimura, H. Yorimitsu, *Org. Lett.* **2016,** *18*, 3674–3677.
24. H. Yi, L. Niu, S. Wang, T. Liu, A. K. Singh and A. Lei, *Org. Lett.*, 2017, *19*, 122–125.
